# Integrated analysis of differential intra-chromosomal community interactions: A study of breast cancer

**DOI:** 10.1016/j.artmed.2025.103180

**Published:** 2025-05-24

**Authors:** Zhihao Yao, Kun Fang, Gege Liu, Magnar Bjørås, Victor X. Jin, Junbai Wang

**Affiliations:** a Department of Clinical Molecular Biology (EpiGen), Akershus University Hospital and University of Oslo, Lørenskog, Norway; b Department of Microbiology, Oslo University Hospital and University of Oslo, Oslo, Norway; c Division of Biostatistics, Data Science Institute, Medical College of Wisconsin, Milwaukee, WI 53226, USA; d MCW Cancer Center, Medical College of Wisconsin, Milwaukee, WI 53226, USA; e Mellowes Center for Genomic Sciences and Precision Medicine, Medical College of Wisconsin, Milwaukee, WI 53226, USA; f Department of Pathology, Oslo University Hospital - Norwegian Radium Hospital, Oslo, Norway; g Department of Clinical and Molecular Medicine, Norwegian University of Science and Technology, Trondheim, Norway

**Keywords:** 3D genome, Differential interaction, Breast cancer, Network community, Epigenetics

## Abstract

It is challenging to analyze the dynamics of intra-chromosomal interactions when considering multiple high-dimensional epigenetic datasets. A computational approach, differential network analysis in intrachromosomal community interaction (DNAICI), was proposed here to elucidate these dynamics by integrating Hi-C data with other epigenetic data. DNAICI utilized a novel hyperparameter tuning method, for optimizing the network clustering, to identify valid intra-chromosomal community interactions at different resolutions. The approach was first trained on Hi-C data and other epigenetic data in an untreated and one hour estrogen (E2)-treated breast cancer cell line, MCF7, and uncovered two major types of valid intra-chromosomal community interactions (active/repressive) that resembles the properties of A/B compartments (or open/closed chromatin domains). It was further tested on the breast cancer cell line MCF7 and its corresponding tamoxifen-resistant (TR) derivative, MCF7TR, and identified 515 differentially interacting and expressed genes (DIEGs) within intra-chromosomal community interactions. In silico analysis of these DIEGs revealed that endocrine resistance is among the top biological pathways, suggesting an interacting/looping-mediated mechanism in regulating breast cancer tamoxifen resistance. This novel integrated network analysis approach offers a broad application in diverse biological systems for identifying a biological-context-specific differential community interaction.

## 1. Introduction

Over the past decade, the advancement of both genomic profiling and computational analysis has revolutionized the field of three-dimensional (3D) chromatin architecture [1–7]. The human genome is now known to function as a complex hierarchy of folded high-order chromatin units, such as chromosomal territories, compartments, topologically associating domains (TADs), and chromatin loops [7,8]. Chromatin or DNA looping is recognized as one of the fundamental principles of gene regulation, with enhancers interacting with target promoters in the nucleus [9,10]. An increasing amount of evidence has demonstrated that chromosomal interactions between genomic regions, spanning tens of kilobases (Kb) to megabases (Mb), are crucial for transcription. These interactions are particularly significant in the context of enhancer-promoter connections and other functional elements [11–13]. Chromatin modifications further enhanced the diversity of gene regulation, underscoring the need to integrate chromatin interactions and epigenetic data to better decipher the complexity of chromatin architecture.

High-throughput chromosome conformation capture (Hi-C) is a widely used technique to study chromatin 3D interactions involving DNA regions of interest [1,5,14–16]. The complex physical contacts in chromosome space can be simplified into a Hi-C contact matrix, in which genomic loci represent nodes and chromosome-chromosome interactions can be regarded as links in this network [17,18]. We and others have demonstrated that although chromatin interaction domains are conserved at one Mb size among different cell types, hundreds of Kb-size subdomains are usually diverse and dynamically changed, especially at different time points upon an environmental or cellular stimulus [19–25]. This indicates the functional modules at different levels in a network could be affected by changes in node contacts, especially when genetic/epigenetic changes occur simultaneously. Therefore, applying differential analysis on dynamically changing structures is a biologically meaningful task, which could help locate potential key transcriptional elements [26–28].

Computational tools have been sprouting for differential analysis from Hi-C contact matrices towards multiple cell types and temporal cellular stimuli [29–32]. High quality differential compartments, TADs, and chromatin loops have become available with unprecedented high-resolution datasets. For instance, dcHiC identifies differential compartments by measuring Mahalanobis distance [32]; DiffGR can robustly discover differential genomic regions at TAD level by measuring similarity of local TAD regions [33]; And diffHic performs biological meaningful interactions detection based on the statistical framework of edgeR [34,35]. However, current methods are mainly focused on comparisons between chromatin structures at a fixed scale (either compartments, TADs, or loops), overlooking the multiscale topology of chromatin folding. In addition, inferences solely from Hi-C contact maps without incorporation of epigenetic modifications, are insufficient to draw functional chromosome-chromosome interaction changes and to comprehensively understand 3D genome regulation. Therefore, a computational approach that systematically integrates Hi-C data with other omics data is crucial for revealing multi-level chromatin dynamics. This integration is essential for deepening our understanding of the normal and disease development, improving treatment strategies across various cell types and at different time points.

**Fig. 1.**
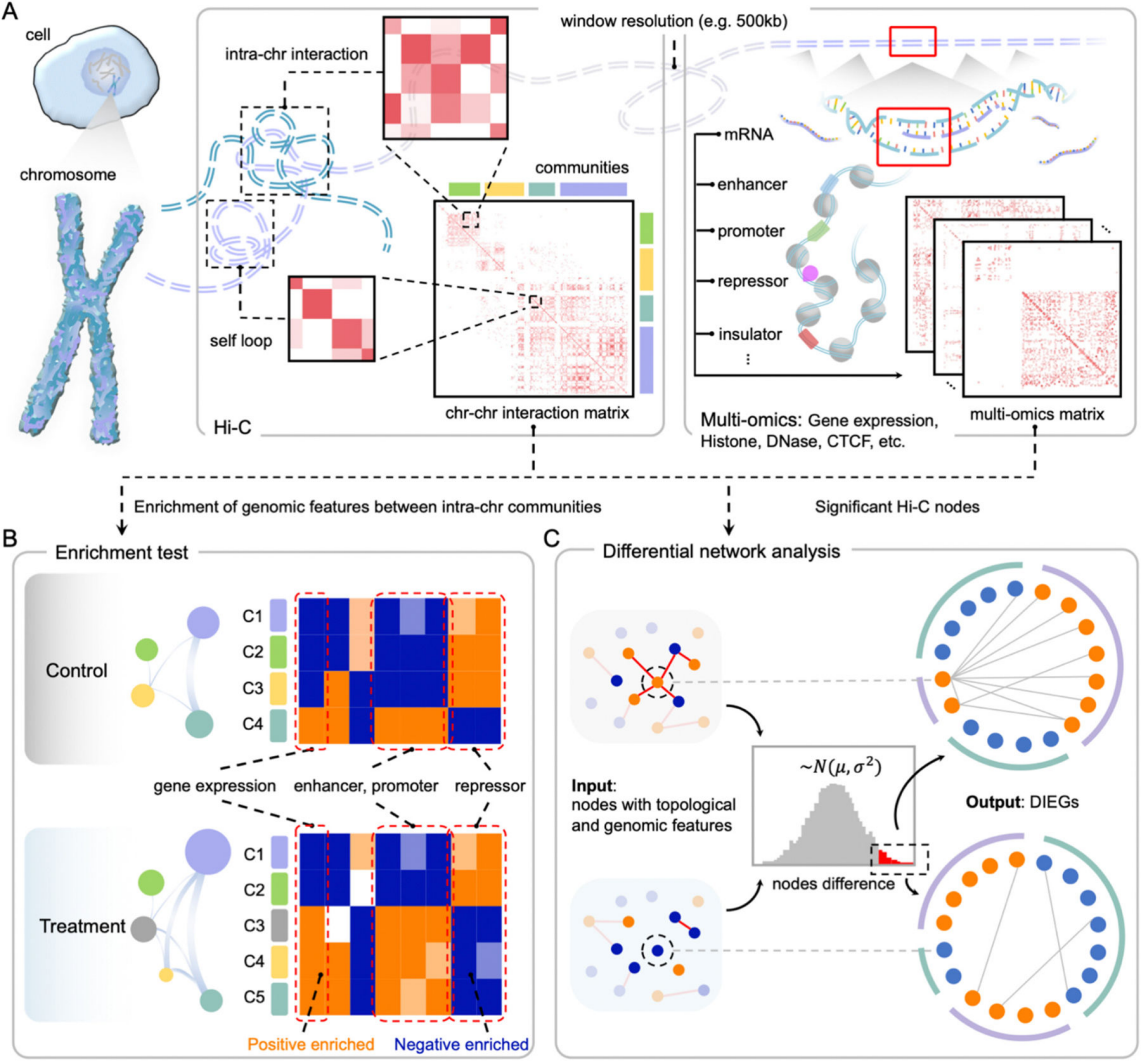
A graphical illustration of integrative network analysis with both the Hi-C interactions and the multi-omics data. (A) For each chromosome, intra-chromosomal interactions under a particular condition/cell line obtained from a Hi-C sequencing experiment, which were mapped to a binary adjacency matrix A with a predefined resolution such as window bin size = 500 kb. Meanwhile, multi-omics data such as histone modifications (enhancer mark, repressive mark), nucleosome density, and gene expressions are mapped to genomic feature matrices F, based on the Hi-C interactions (or adjacency matrix A*)* with the same window bin size. (B) For E2 treated MCF7 cells and MCF7TR cells, intra-chromosomal communities (C_1_, C_2_, ….) were identified by applying SLM algorithm on the unweighted adjacency matrix *A*. Then, a random permutation *t*-test was used to evaluate the enrichment of genomic features in the communities, where the log10 transformed expected *P*-value with the sign of expected T-value were converted to color coded heatmap, for assessing the significant positive or negative enrichment of genomic features in intra-chromosomal communities. (C) Differential network analysis was applied on the Hi-C intra-chromosomal interactions by combining both topological features and genomic features. Euclidean distance was used to assess the difference between nodes from E2 treated MCF7 cells and MCF7TR cells, respectively. Significant differential interacting expressed nodes, genes, and sub-networks were identified by setting thresholds of fitted Gaussian distribution and relative ratio (P-value <0.05, Absolute (relative ratio) > 0.66).

To address the aforementioned limitations, we developed a novel integrated network analysis approach using Hi-C data and epigenetic modifications, DNAICI (Differential Network Analysis in Intra-chromosomal Community Interaction), for identifying significant chromatin variations from Mb-size intra-chromosomal community structures to Kb-size differential interacting and differentially expressed genes. The framework of DNAICI (Fig. 1) is composed of 1) constructing genomic feature matrices by mapping multi-omics datasets to adjacency matrices obtained from Hi-C data, 2) identifying valid community structures based on both the network clustering and the optimized parameter determination methods, 3) evaluating the enrichment of genomic features in intra-chromosomal community interactions with a permutation test, and 4) screening for significant gene loci and corresponding sub-network by comparing genomic and network topological features through analyzing differential intra-chromosomal community interaction. We applied our computational approach on an ERα + breast cancer model system, including ERα + tamoxifen-sensitive cell line MCF7 at untreated and estrogen (E2)-treated conditions, as well as tamoxifen-resistant cell line MCF7TR. The reasons for choosing this model system to evaluate DNAICI are: 1) our previous studies found that E2-mediated highly dynamic chromatin domains showed higher alteration in tamoxifen-resistant breast cancer cells [19], thus, it is critical to further assess chromatin structure reorganization by an integrated computational approach; 2) this breast cancer model system has been studied by us and many other people for many years [26,36–40], there are plenty of published genome-wide datasets that can be used for integrated analysis; and 3) MCF7 and MCF7TR cells share strong similarities with clinical characteristics of endocrine sensitive and resistant breast cancer [9,19]. Our goal is to gain insights into the functional role of these interactions in contributing to breast cancer tamoxifen resistance from a 3D multi-omics perspective. Moreover, we anticipate that applying and validating our novel computational tool in an ERα + breast cancer model system will facilitate its broader application in many other biological systems, for identifying a biological-system-specific differential community interaction.

## 2. Material and methods

### 2.1. Epigenetic data used in this study

Hi-C data in untreated and one hour of E2-treated MCF7 cells, and MCF7TR cells were obtained from our previous publication [19]. Preprocessing of raw Hi-C data was performed by HiCUP [41] such as mapping reads to HG19 human reference genome and so on. Aligned BAM files of Hi-C data were further processed by either in-house python scripts (e.g., normalization [42] and transformation of Hi-C interaction data to *Z*-scores [26]) or HOMER [43] for selecting significant chromosomal interactions. Then, intra-chromosomal interactions were studied in 500 kb, 100 kb, and 50 kb resolution, respectively. Microarray expression profiles of genes in zero and one hour of E2 treated MCF7 cells, as well as RNA-Seq data in MCF7TR cells were retrieved from earlier studies [44,45], which were quantile normalized [46] before transforming them to *Z*-scores. ChIP-Seq data of marks for enhancer (H3K27ac and H3K4me1), promoter (H3K4me3), repressor (H3K27me3 and H3K9me3), nucleosome density (DNase-seq and ATAC-Seq), and insulator (CTCF) in the same MCF7 cells were downloaded from published work [19,45] and ENCODE project [47]. Preprocessing of these ChIP-Seq and ATAC-Seq data was done similarly as published works [19,26,48]: for example, raw reads were mapped to HG19 human reference genome by bowtie2 [49], narrow (CTCF, H3K27ac and H3K4me3) and broad (DNase, H3K27me3, H3K4me1 and H3K9me3) peaks were called by MACS2 [50], and problematic regions for high throughput sequencing in human genome were removed according to ENCODE Black List [51]. Then, BEDTools [52] were used to estimate the read counts in a 200 bp window size for called peaks which were quantile normalized across all marks [53] before log-transforming and converting them to *Z*-scores. The full datasets (Hi-C data, epigenetic modifications from MCF7 cells with untreated, one hour of E2 treated and MCF7TR cells), the Supplementary Files from this study, as well as the Python package – DNAICI with the step-by-step tutorials for both preprocessing multi-omics data and downstream differential network analysis, are available on the project web page https://differential-network-analysis.github.io/dnaici-webpage/.

### 2.2. Identifying significant intra-chromosomal interactions from Hi-C interactions

For removing unreliable or biased intra-chromosomal interactions from Hi-C data, two approaches were used to screen significant chromosomal interactions. One was to filter the weakest 0, 5, 10, 20, or 60 percentages of intra-chromosomal interactions from observed Hi-C interactions, while the other one was to apply HOMER [43] directly on the aligned BAM files of Hi-C data, to obtain significant interacting fragments [27] such as using “makeTagDirectory” and “analyzeHiC” with significant *P*-value <0.1. This chosen P-value cutoff may remove the most unreliable interactions from the experiments, but retain some of the weak interactions that may be biologically meaningful [26]. The screened significant intra-chromosomal interactions were converted to adjacency matrices A (or intra-chromosomal interaction matrices) with a predefined window bin size (see Supplementary Methods: Building adjacency matrices with predefined resolution for Hi-C interactions). Both filtering approaches were applied for screening observed intra-chromosomal interactions (window bin size = 500 kb) in untreated and one hours E2-treated MCF7 cells, respectively. The aim is to evaluate the robustness of predicted intra-chromosomal community interactions in different filtering criteria such as the network modularity scores, the number of network edges, and the number of valid communities or network clusters.

Since identified significant chromosomal interactions (adjacency matrices of intra-chromosomal interactions) are frequently affected by the program parameters (e.g., in HOMER), it is essential to find optimal parameters at different interaction resolutions such as window bin size equals 500 kb, 100 kb, or 50 kb. Though a higher interaction resolution (or a smaller window bin size) may yield more accurate network structure, it cannot be increased infinitely. For instance, the significant interactions identified by HOMER are dependent on two major parameters: window resolution – bin size, and its super resolution – range of signal averaging. To find optimal parameters for identifying significant intra-chromosomal interactions by HOMER, a new approach was designed to evaluate the associations between the number of identified interactions and the distance of interactions, when different window bin sizes were used in analysis. This new statistical method can minimize the impact of window bin sizes on building adjacency matrices, when applying HOMER on Hi-C interactions. In the end, the significant intra-chromosomal interactions identified by HOMER with optimized parameters were used further in differential network analysis.

### 2.3. Applying modularity function to find communities in intra-chromosomal interactions

An intra-chromosomal interaction matrix from Hi-C data is represented by an adjacency matrix in graph theory, where intra-chromosomal interactions are treated as an undirected network. It is interesting to see whether there are intra-chromosomal community interactions in the network or not. For example, a smart local moving (SLM) algorithm [54] can be applied on the Hi-C intra-chromosomal interactions (or an adjacency matrix), to find community structure of intra-chromosomal interactions. The SLM method is based on a modularity function [55] for detecting communities in a large network. The modularity function was proposed by Newman and Girvan [55] for evaluating whether the division of a network into communities is good or bad. Let $A_{ij}$ be a node in an adjacency matrix A of the network, equivalent to a window bin in a Hi-C intra-chromosomal interaction matrix, if $A_{ij}=1$ then two nodes (or window bins $w_{i}$ and $w_{j}$) are connected, otherwise $A_{ij}=0$. Currently, only unweighted networks are considered in building the network communities. The fraction of edges that are assigned to communities (or a node i belong to community $c_{i}$) is

(1) $$FE=\frac{\sum_{ij}\;\;A_{ij}\delta \left(c_{i},c_{j}\right)}{\sum_{ij}\;\;A_{ij}}=\frac{1}{2m}\sum_{ij}\;A_{ij}\delta \left(c_{i},c_{j}\right),$$

where $\delta \left(c_{i},c_{j}\right)=1$ if $c_{i}=c_{j}$ and 0 otherwise, and m is the total number of edges in the graph. The expected fraction of edges assigned to communities in a randomized network is

(2) $$E=\frac{1}{2m}\sum_{ij}\;\frac{k_{i}k_{j}}{2m}\delta \left(c_{i},c_{j}\right),$$

where $k_{i}=\sum_{j}\;A_{ij}$ is the degree of node i in a network. Subsequently, a modularity function Q=FE-E [54] to evaluate the division of the network can be obtained. If the FE is close to E, except in the case of randomized network, then the modularity score Q will be close to zero. In order words, a higher value from modularity function Q indicates a better division of community structure in a network. Usually, a modularity score Q above 0.3 may suggest a significant community structure in a network [55]. To search for optimized community structures of a big network based on the modularity function, SLM algorithm [54] was used to repeatedly move one node from one community to another that increasing the modularity score Q. After many iterations, the final assignment of nodes to communities was reached if no nodes can be moved further to increase the value of Q. More detailed descriptions of SLM algorithms in large network structure studies were provided in previous publications [54–56].

### 2.4. Determining the minimum size of valid communities

Here, a new method was developed to estimate the minimum size of valid communities following a specific window bin size or interaction resolution, after finding communities in complex networks. For example, a minimum size of valid communities (or network clusters) is determined by the predicted communities of intra-chromosomal interactions at a predefined resolution such as bin size equals 500 kb. First, the communities with only one node are removed, and the number of identified communities in a chromosome r is defined as $C_{r}$. Then, the number of edges in a community (or network cluster) j for chromosome r is defined as $e_{rj}$, where r∈{1,2,⋯,23}, and $j\in \left\{1,2,\cdots ,C_{r}\right\}$. The value of $e_{rj}$ is closely related to the number of nodes in a network. If a network is built with different resolutions such as window bin size equals to 500 kb or 50 kb, then the value of $e_{rj}$ will be changed simultaneously. Thus, $e_{rj}$ has to be normalized against a specific network resolution (or window bin size) by equation

(3) $$p_{rj}=\frac{e_{rj}}{\bar{e_{j}}},$$

where $\bar{e_{j}}=\frac{\sum_{r.j}\;\;e_{rj}}{\sum_{r}\;\;C_{r}}$ is the average number of edges of all chromosomes at the same network resolution. Subsequently, a predefined percentage value p can be set to find communities with at least p percentage (e.g., p=2% in this work) of the average number of edges, based on equation

(4) $$p^{*}={argmin}_{p_{rj}}\left|p_{rj}-p\right|,$$

where the closest value $p^{*}$ to p is identified and the corresponding number of expected edges $e^{*}$ is calculated by

(5) $$e^{*}=p^{*}\bar{e_{j}},$$

which is the expected minimum size (or number of edges) of valid communities in a network with a chosen interaction resolution. Based on the predefined threshold value p, the expected minimum size of valid communities in a community interaction network can be determined by $e^{*}$ (e.g., the number of edges for valid communities $>e^{*}$) and estimated automatically for different Hi-C interaction networks at different interaction resolutions (or bin sizes). Please note that the edges of intercommunity interactions were not considered in the calculation. Thus, the number of identified total communities of chromosome r were reduced from $C_{r}$ to $V_{r}$ after filtering communities that do not reach the minimum size (or the number of edges) of valid ones.

### 2.5. Inferring communities and genomic feature matrices based on intra-chromosomal interactions

First, an intra-chromosomal interaction matrix H (a normalized Hi-C contact count or *Z*-score matrix) is converted to an adjacency matrix A, based on a predefined resolution such as bin size equals 500Kb. Please refer to Supplementary Methods - Building adjacency matrices with predefined resolution for Hi-C interactions. Then, multi-omics datasets $H^{\left(1\right)},\cdots ,H^{\left(G\right)}$ from various genomic features were used to build genomic feature matrices $F^{\left(1\right)},\cdots ,F^{\left(G\right)}$ follow the adjacency matrix A, where G is the number of genomic features (e.g., gene expressions, nucleosome density/DNase-seq, histone modifications for enhancer “H3K4me1 and H3K27ac”, promoter “H3K4me3”, repressor “H3K9me3 and H3K27me3”, or insulator “CTCF”). Please refer to Supplementary Methods - Generating genomic feature matrices based on multi-omics data for Hi-C interactions. The weights of genomic features are assigned to the edges of feature matrix F based on intra-chromosomal interactions in adjacency matrix A. Subsequently, SLM algorithm is applied on the unweighted adjacency matrix A, with a window bin size equals 500 kb, to predict intra-chromosomal community interactions. After determining the minimum size (or number of edges) of valid communities, the valid communities V (or network clusters) were used in further data analysis such as the genomic feature enrichment and the differential network analysis.

### 2.6. Evaluating genomic feature enrichment in intra-chromosomal interaction communities

Based on the community network, an enrichment of various genomic features in valid intra-chromosomal communities can be evaluated [57,58]. For instance, to evaluate the difference of genomic feature *l* between the inside community ($F_{v}^{\left(l\right)}$) and the outside community ($F_{v^{'}}^{\left(l\right)}$), a two-sample *t*-test is applied on each valid community (or a network cluster), such as the T-value of feature l is

(6) $$T_{i}^{\left(l\right)}=T_{\text{test}}\left(F_{v}^{\left(l\right)},F_{v^{'}}^{\left(l\right)}\right),$$

where $F_{v}^{\left(l\right)}$ represents the observed genomic feature l within the valid community v, while $F_{v^{'}}^{\left(l\right)}$ is the expected genomic feature from randomly selected intra-chromosomal interactions (drawn from outside community v but with the same size as $F_{v}^{\left(l\right)}$). The *P*-value $P_{i}^{\left(l\right)}$ corresponding to $T_{i}^{\left(l\right)}$ indicates whether there is a significant enrichment of genomic feature l at the community in sampling i or not. After resampling N times, an expected P-value ($P^{\left(l\right)}$) for the enrichment of genomic feature l in a community can be inferred from permutation test

(7) $$P^{\left(l\right)}=\frac{\sum_{i=1}^{N}\;\;I_{\left\{P_{i}^{\left(l\right)}<\alpha \right\}}}{N},$$

where α is a predefined significance level (e.g., α=0.05). Meanwhile, an expected T-value for the enrichment of genomic feature l in N samplings is

(8) $$T^{\left(l\right)}=\frac{\sum_{i=1}^{N}\;\;T_{i}^{\left(l\right)}}{N}.$$

Here, the smaller the expected P-value $P^{\left(l\right)}$ the more significant the genomic feature l in the community. The sign of $T^{\left(l\right)}$ indicates a positive or negative enrichment of the feature in the community. By combining information of both $P^{\left(l\right)}$ and $T^{\left(l\right)}$, a score of feature enrichment $E^{\left(l\right)}$ that similar to previous works [58,59] is proposed

(9) $$E^{\left(l\right)}=sgn\left(T^{\left(l\right)}\right)*\left|\log_{10}P^{\left(l\right)}\right|,$$

where sgn(x) is the sign function that equals 1, −1, and 0 when the sign of x is positive, negative, and x is zero, respectively. Taken together, $E^{\left(l\right)}$ tells both the significance and the direction (positive or negative) of genomic feature enrichment in intra-chromosomal communities. A parallel computation was implemented in DNAICI to perform random permutation tests of each genomic feature in a community (or network cluster), for accessing the enrichment of genomic features in the communities.

### 2.7. Differential network analysis - identifying significant intra-chromosomal interaction changes

Information of both the intra-chromosomal interaction network from Hi-C data and the genomic feature enrichment in the communities are considered in differential network analysis. Firstly, the network structure information (node centrality) of intra-chromosomal interactions were obtained in different conditions from the adjacency matrices *A*, respectively, which follows a specific resolution such as Hi-C interactions with a window bin size 500 kb. The degree centrality of a node *w* in a network can determine the importance of the node within the network based on its position [60], which is

(10) $$Centrality(w)=\frac{\text{degree}(w)}{n-1}$$

where w is a given node in an intra-chromosomal interaction network, n is the total number of nodes, and degree(w) is the number of interactions connected to node w. A significant change of a node centrality between the conditions, may indicate its importance in perturbating network structure following condition change. Secondly, nodes located in the same community share the same genomic feature l enrichment in the community, such as the expected T-value of genomic feature l enrichment at a community is shared by nodes within the same community. Subsequently, both the node centrality and the expected T-values of genomic feature l enrichment at the node w were converted to *Z*-score and built into a vector $Z_{w}\left(z_{\text{Centrality}},\cdots ,z_{T^{\left(l\right)}},\cdots \right)$ for further differential network analysis.

To identify significant intra-chromosomal interaction changes, the aforementioned procedure was repeated in two intra-chromosomal interaction networks derived from Hi-C data of MCF7 cells and MCF7TR cells, respectively, where Z-score vectors ($Z_{w}$ and $Z_{w^{'}}$) of nodes were obtained. The Z-score vectors contained both the node centrality in a network and the genomic feature enrichments at the node, which can be used directly for differential network analysis, such as to compute a Euclidean distance between $Z_{w}$ and $Z_{w^{'}}$

(11) $$Dist\left(w,w^{'}\right)=\left\|Z_{w}-Z_{w^{'}}\right\|,$$

where ‖•‖ represents the $\ell_{2}$ norm. Consequently, nodes between the conditions (e.g., MCF7 cells versus MCF7TR cells) can be quantified by Euclidean distance. Subsequently, all Euclidean distances were mapped to a Gaussian distribution for finding a cutoff value $d^{*}$, where nodes with Euclidean distance greater than the $d^{*}$ are

(12) $$S_{\text{node}}=\left\{w|Dist\left(w,w^{'}\right)>d^{*}\right\},$$

and $S_{\text{node}}$ are nodes with significant changes between the two conditions in the networks. For example, a cutoff value $d^{*}$ corresponding to the top 5 % of $Dist\left(w,w^{'}\right)$ (or the expected *P*-value =0.05) that fits a Gaussian distribution. Thus, a set of nodes with significant Euclidean distance changes (or intra-chromosomal interaction changes) between the conditions can be identified. An evaluation of robustness of *Z*-score calculations in differential network analysis was also carried out. Please refer to Supplementary Methods – A comparison of genomic feature enrichments between the mean and the median mapping of Z-scores to Hi-C interaction matrix. Finally, differential interacting and differentially expressed genes (DIEGs) were detected from these $S_{\text{node}}$ and visualized in a network. Based on DIEGs, gene ontology (GO) analysis and biological pathway enrichment tests were performed by DAVID tool [61] for further biological investigation.

## 3. Results

### 3.1. Generating genomic feature matrices based on intra-chromosomal interactions

The proposed integrative data analysis pipeline (Fig. 1A) was utilized to investigate Hi-C intra-chromosomal interactions in untreated and one hours E2-treated MCF7 cells, respectively. First, *Z*-scores transformed intra-chromosomal interaction matrices were built based on the normalized Hi-C contact count matrices [42] with previously published procedures [26]. Then, significant intra-chromosomal interactions were identified by HOMER [43] in 500 kb resolution. The two results were converted to an adjacency matrix A with window bin size 500 kb, respectively. In order to minimize the impact of random (or bias) interactions on the analysis [62], the first result was further filtered by removing the weakest 5, 10, 20, 40, and 60 percentages of intra-chromosomal interactions before building adjacency matrices, respectively. Based on these intra-chromosomal interaction matrices (or adjacency matrices), the contribution of genomic features in interactions (a pair of window bins or nodes with an edge in a graph) were estimated by a weighted edge approach [63]. Subsequently, genomic feature matrices F based on the adjacency matrices were built for each feature in every chromosome, respectively. For example, in Supplementary Figs. (S1, S2 and S3), heatmaps of both intra-chromosomal interaction matrices (or weighted adjacency matrix A) and the corresponding genomic feature matrices F (e.g., gene expression, nucleosome density/DNase, insulator/CTCF, and histone modifications for enhancer, promoter and repressor) before filtering weakest interactions, were shown for chromosomes 3, 17, and 20 in zero hours E2 treated MCF7 cells, respectively. Here, it is not easy to perform biological interpretation of these heatmaps due to the large size of networks, though all information comes from the same cell line or condition. Thus, a network clustering or community prediction approach is needed.

### 3.2. Identifying intra-chromosomal community interactions

**Fig. 2.**
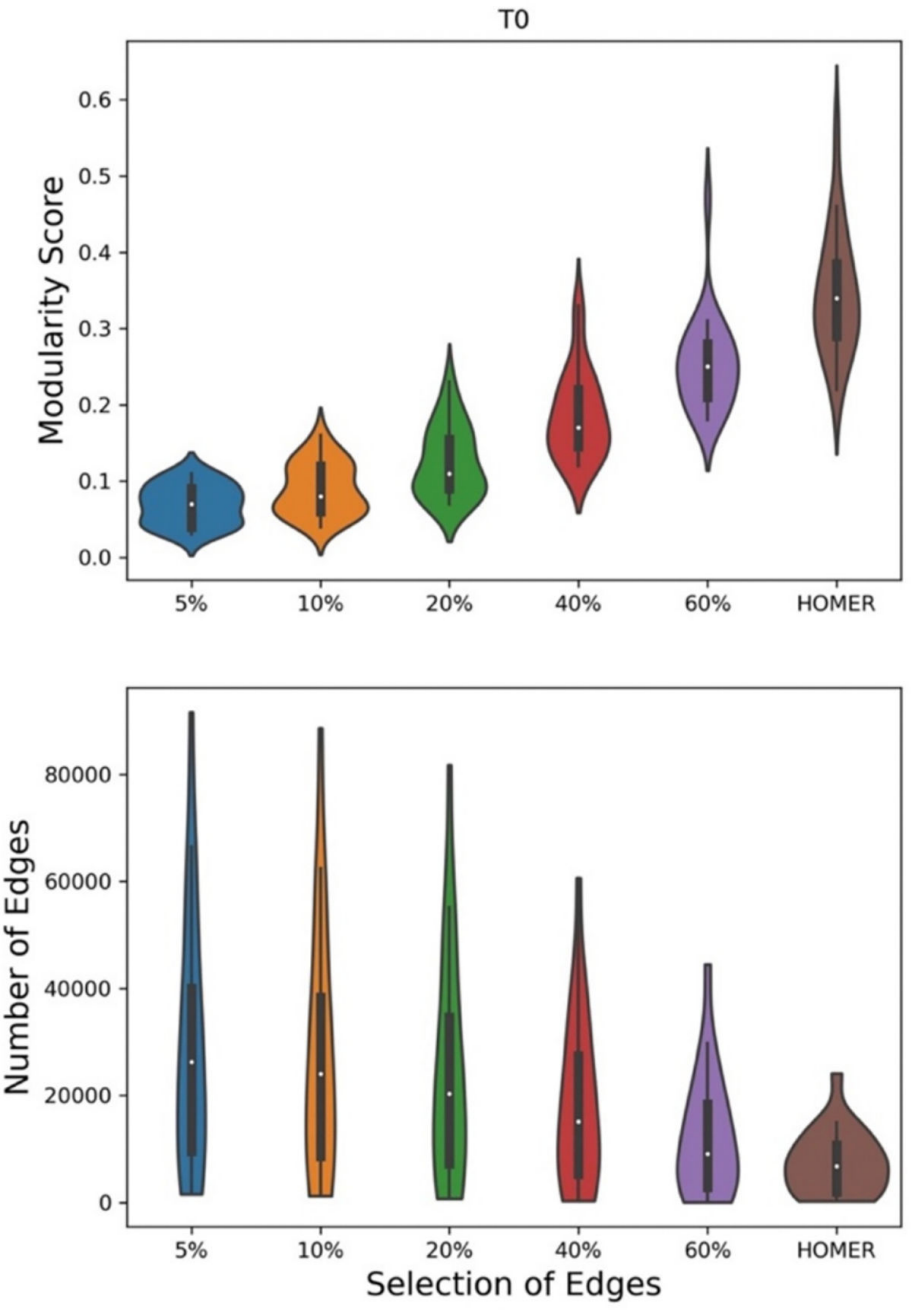
Violin plot of modularity scores and number of edges based on different selection of intra-chromosomal interactions in Hi-C data at 500 kb resolution for untreated MCF7 cells. The upper panel of figure showing violin plots of modularity scores [55] for intra-chromosomal community interactions in 23 chromosomes that were calculated based on the filtering of 5, 10, 20, 40, 60 percentages of the weakest intra-chromosomal interactions genome-widely, respectively. “HOMER” represented the significant intra-chromosomal interactions predicted by the HOMER [43] program. The lower panel of figure displayed violin plots of the number of edges (or intra-chromosomal interactions) in 23 chromosomes after filtering 5, 10, 20, 40, 60 percentages of the weakest intra-chromosomal interaction genome-widely or selecting significant interactions by HOMER. A white dot in the violin plot represented the median value of the distribution.

Here, a modularity function and a smart local moving (SLM) algorithm [54] were applied to predict intra-chromosomal community interactions, based on unweighted adjacency matrices A derived from either the significant intra-chromosomal interactions provided by HOMER or the Hi-C intra-chromosomal interactions after filtering the weakest 5, 10, 20, 40, and 60 percentages of interactions, respectively. Fig. 2 and Supplementary Fig. S4 show that the median number of edges (or interactions) in A is between 8000 and 25,000 for untreated and one hours E2-treated MCF7 cells, respectively. When filtering the weakest interactions, the modality scores of identified intra-chromosomal community interactions increase in both conditions as the percentage of removed weakest interactions rises. The score reaches 0.3 (a median of modularity score < 0.3) when 60 % of weakest intra-chromosomal interactions are filtered, which indicates there is a community structure in the network [55]. Nevertheless, the score is still smaller than that based on the significant interactions (a median of modularity scores>0.3) identified by HOMER.

**Fig. 3.**
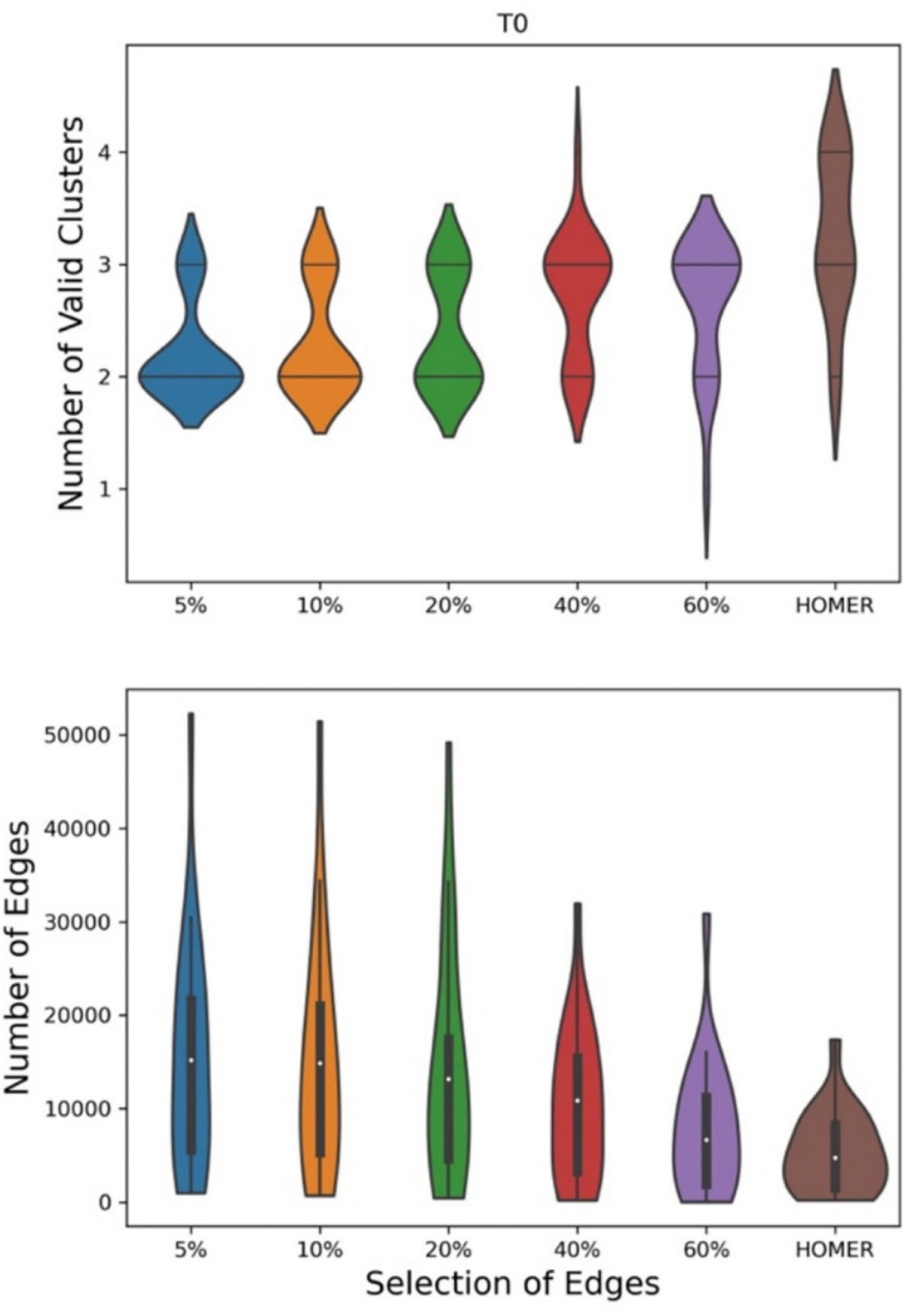
Violin plot of number of valid clusters and number of edges in valid clusters based on different selection of intra-chromosomal interactions in Hi-C data at 500 kb resolution for untreated MCF7 cells. The upper panel of figure shows violin plots of the number of valid network clusters such as communities with the number of edges >20 in intra-chromosomal interactions among 23 chromosomes, which were predicted by filtering of 5, 10, 20, 40, 60 percentages of the weakest intra-chromosomal interaction genome-widely, respectively. “HOMER” represents the selection of significant intra-chromosomal interactions by the HOMER [43] program. Here, a black line within each violin plot represents every observation inside the distribution. The lower panel of figure displayed violin plots of the number of edges (or intra-chromosomal interactions) in valid clusters of intra-chromosomal interactions among 23 chromosomes after filtering of 5, 10, 20, 40, 60 percentages of the weakest intra-chromosomal interaction genome-widely or selecting significant interactions by HOMER. A white dot in the violin plot represented the median value of the distribution.

Fig. 3 and Supplementary Fig. S5 were violin plots of the number of valid communities (or network clusters) and the number of edges (or interactions) in valid communities, for Hi-C data of untreated and one hours E2-treated MCF7 cells, respectively. The results indicated that there were either two or three valid communities in intra-chromosomal interaction networks, regardless of the filtering criteria for weak interactions. The median number of edges in valid communities was between 5000 and 15,000 (Fig. 3 and SFigure 5), where more than half of intra-chromosomal interactions were assigned to valid communities (or clusters) in each chromosome. Therefore, the filtering of weak (or identification of significant) intra-chromosomal interactions was essential for revealing the true community structure of intra-chromosomal interactions. For the simplicity of current analysis, a manually selected cutoff value (minimum size of edges $e^{*}>20$) was used to define the valid intra-chromosomal communities (or network clusters) at 500 kb resolution. More information of the intra-chromosomal community interactions, such as the modularity scores and the number of valid clusters in a chromosome, based on the significant interactions from HOMER and the filtering of weakest interactions are shown in Supplementary Tables S1 to S12, respectively.

### 3.3. Enrichment of genomic features in intra-chromosomal community interactions

**Fig. 4.**
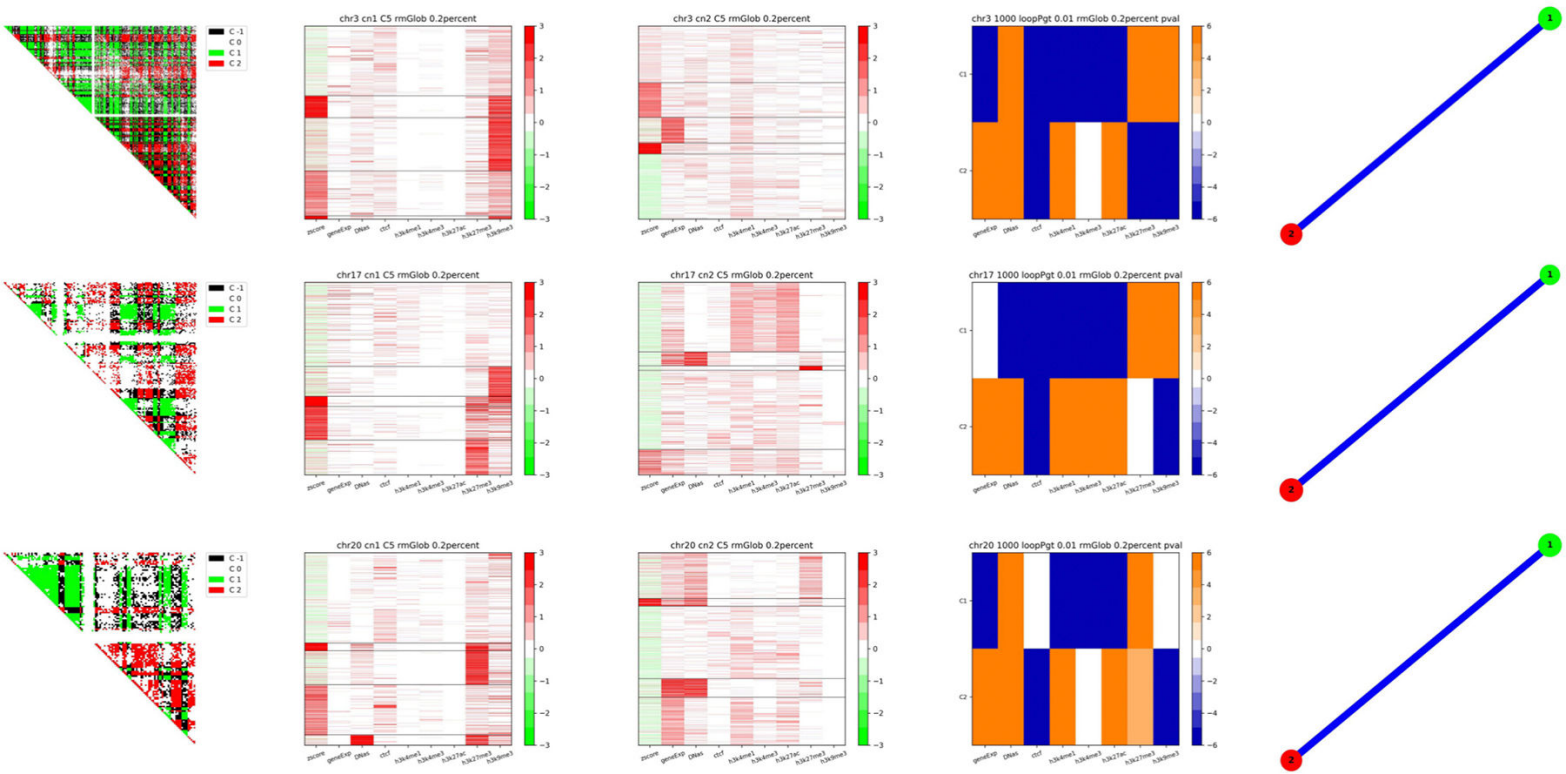
Heatmaps of intra-chromosomal community interactions for chromosomes 3, 17 and 20 after removing the lowest 20 percentages of intra-chromosomal interactions at 500 kb resolution in untreated MCF7 cells. In each panel, *the left most heatmaps* were intra-chromosomal interaction matrices where interactions were colored by their community (or network cluster) labels. For example, black indicates two nodes of an edge (or interaction) do not belong to the same community, but the other colors represent the community label of an interaction. *The right most figures* were network plots of predicted intra-chromosomal community interactions (only show communities with >20 edges) in a chromosome, where the size of nodes and the width of edges indicates the number of edges (interactions) in the community and the number communications between the communities, respectively. The color of nodes indicates the community label. In a panel, *the second right most figures* were heatmaps of log10 transformed *P*-values for enrichment of a genomic feature in a community (or network cluster) against that from randomly generated (the number of P-value <0.01 in 1000 times random permutation *t*-tests) intra-chromosomal interactions, where the orange and blue colors represented positive and negative enrichment of the feature, respectively. This sign of the enrichment was inferred from the expected T-values of random permutation t-test. The rest of red and green coded heatmaps were genomic features in intra-chromosomal community interactions: “Zscore” was the *Z*-score of intra-chromosomal interactions; “geneExp”, “DNas” and “CTCF” were average of gene expressions, nucleosome densities, and insulator in the interactions, respectively; “H3K4me1” and “H3K27ac” were enhancer markers; “H3K4me3” is a promoter maker; “H3K27me3” and “H3K9me3” were repressor markers. Here, “cn1” and “cn2” were community number 1 and 2 in a chromosome, respectively, which were the same numbers as the node labels in the network plots. (For interpretation of the references to color in this figure legend, the reader is referred to the web version of this article.)

**Fig. 5.**
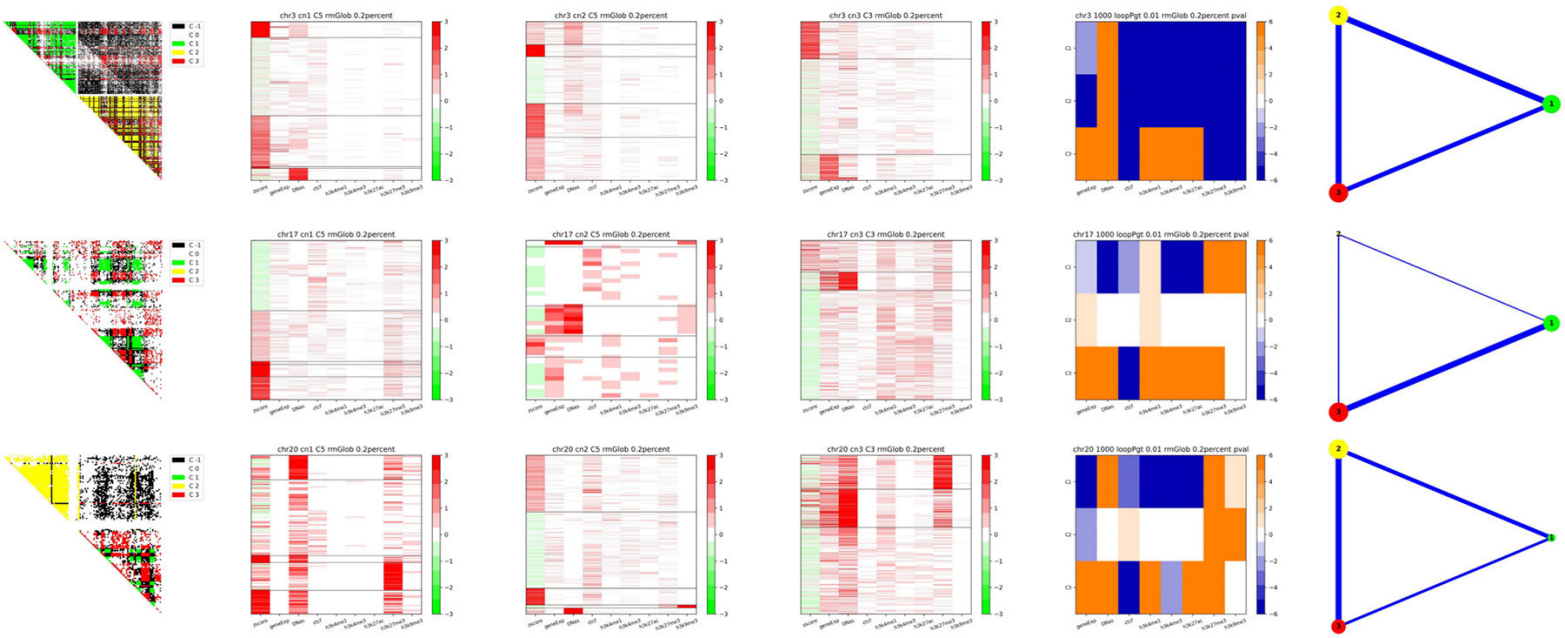
Heatmaps of intra-chromosomal community interactions for chromosomes 3, 17 and 20 after removing the lowest 20 percentages of intra-chromosomal interactions at 500 kb resolution in one-hours E2-treated MCF7 cells. In each panel, *the left most heatmaps* were intra-chromosomal interaction matrices where interactions were colored by their community (or network cluster) labels. For example, black indicated two nodes of an edge (or interaction) did not belong to the same community, but the other colors represent the community label of an interaction. *The right most figures* were network plots of predicted intra-chromosomal community interactions (only show communities with >20 edges) in a chromosome, where the size of nodes and the width of edges indicated the number of edges (interactions) in the community and the number communications between the communities, respectively. The color of nodes indicated the community label. In a panel, *the second right most figures* were heatmaps of log10 transformed *P*-values for enrichment of a genomic feature in a community (or network cluster) against that from randomly generated (the number of P-value <0.01 in 1000 times random permutation t-tests) intra-chromosomal interactions, where the orange and blue colors represent positive and negative enrichment of the feature, respectively. This sign of the enrichment was inferred from the expected T-values of random permutation t-test. The rest of red and green coded heatmaps were genomic features in intra-chromosomal community interactions: “Zscore” was the Z-score of intra-chromosomal interactions; “geneExp”, “DNas” and “CTCF” were average of gene expressions, nucleosome densities, and insulator in the interactions, respectively; “H3K4me1” and “H3K27ac” were enhancer markers; “H3K4me3” is a promoter maker; “H3K27me3” and “H3K9me3” were repressor markers. Here, “cn1”, “cn2”, and “cn3” were community numbers 1, 2, and 3 in a chromosome, respectively, which were the same numbers as the node labels in the network plots. (For interpretation of the references to color in this figure legend, the reader is referred to the web version of this article.)

After intra-chromosomal interactions were summarized by community interactions or network clusters, it became easy to evaluate the enrichment of genomic features in communities. Figs. 4 and 5 were heatmaps of identified intra-chromosomal community interactions (chromosomes 3, 17, and 20) in untreated and one hours E2-treated MCF7 cells, respectively, after filtering the weakest 20 % of intra-chromosomal interactions. The first column of the heatmaps were intra-chromosomal interaction matrices (or weighted adjacency matrices A from Hi-C data), where valid communities (or network clusters) were color coded such as the same community visualized by the same color. The plots revealed that the interactions from the same community (or network cluster) were physically close to each other, which shared similarity with topologically associating domains (TAD). The second last column of Figs. 4 and 5 were the heatmaps of absolute log10 transformed *P*-values with the sign of the T-values from genomic features enrichment test (e.g., gene expression, nucleosome density – DNase, enhancer, promoter, insulator, and repressor markers) in valid communities. These results indicated that there were two major communities in the intra-chromosomal interactions: repressive marks (H3K27me3 and H3K9me3) enriched or active marks (e.g., enhancer and promoter makers H3K4me1, H3K4me3, and H3K27ac) enriched communities. This is analogous to the A/B compartments in the genome that are associated with either open or closed chromatin [64]. This analogy of A/B compartments was observed for all results, regardless of the filtering criteria for intra-chromosomal interactions. Please refer to Supplementary Figs. S6-S11 for results of filtering the weakest 40 % and 60 % interactions, as well as the significant interactions from HOMER, respectively.

In Figs. 4, 5 and Figs. S6–11, the intra-chromosomal community interactions (the last column of the figures) were similar in both conditions, but the enrichment of genomic features in the communities (the second last column of the figures) were different from each other. For example, in chromosome 3 (Fig. 4), the community C1 is enriched by repressor makers H3K27me3 and H3K9me3 (the second last column of Fig. 4) at untreated MCF7 cell, but it is disappeared at one hours E2 treated condition (the second last column of Fig. 5). In chromosome 17, a community C2 is only enriched by enhancer markers (H3K4me1 and H3K27ac) at untreated MCF7 cell (the second last column of Fig. 4), but a similar community C3 is enriched by both the enhancer and the repressor markers (H3K27me3) at one hours E2 treated MCF7 cell (Fig. 5). These results were consistent when different filtering conditions were applied on intra-chromosomal interactions, such as the weakest 20 %, 40 %, 60 % of interactions were removed, or the significant interactions identified by HOMER.

### 3.4. Optimizing the parameters for valid communities at different resolutions

So far, the proposed analysis pipeline was only evaluated at a single resolution (window bin size = 500 kb) with a fixed number of edges for valid communities ($e^{*}>20$). The results indicate that the quality of inferred community structures is better by using significant chromosomal interactions identified by HOMER than that by an ad hoc filtering of the weakest interactions (Figs. 2 and 3). For studying intra-chromosomal interactions in different resolutions, our newly proposed methods were used to fine-tune HOMER parameters and to estimate a minimum size of valid communities. For example, if the window bin resolution is 50 kb, 100 kb and 500 kb, then the super resolution in HOMER for identifying significant chromosomal interactions is 500 kb, 100 kb and 50 kb, respectively. For studying the intra-chromosomal interactions at different resolutions (or window bin size), the minimum size (or the number of edges) of valid communities is ~20, ~100 and ~ 200 when window bin size equals to 500 kb, 100 kb and 50 kb, respectively. For a detailed description of optimizing the aforementioned parameters in different interaction resolutions please refer to Supplementary Results – Finding parameters for HOMER and Optimizing the parameters for valid communities at different resolutions, respectively, Supplementary Figs. (S12, S13, S14 and S15), and Supplementary Tables (S13).

### 3.5. Robustness of genomic feature enrichments in communities at different resolutions

**Fig. 6.**
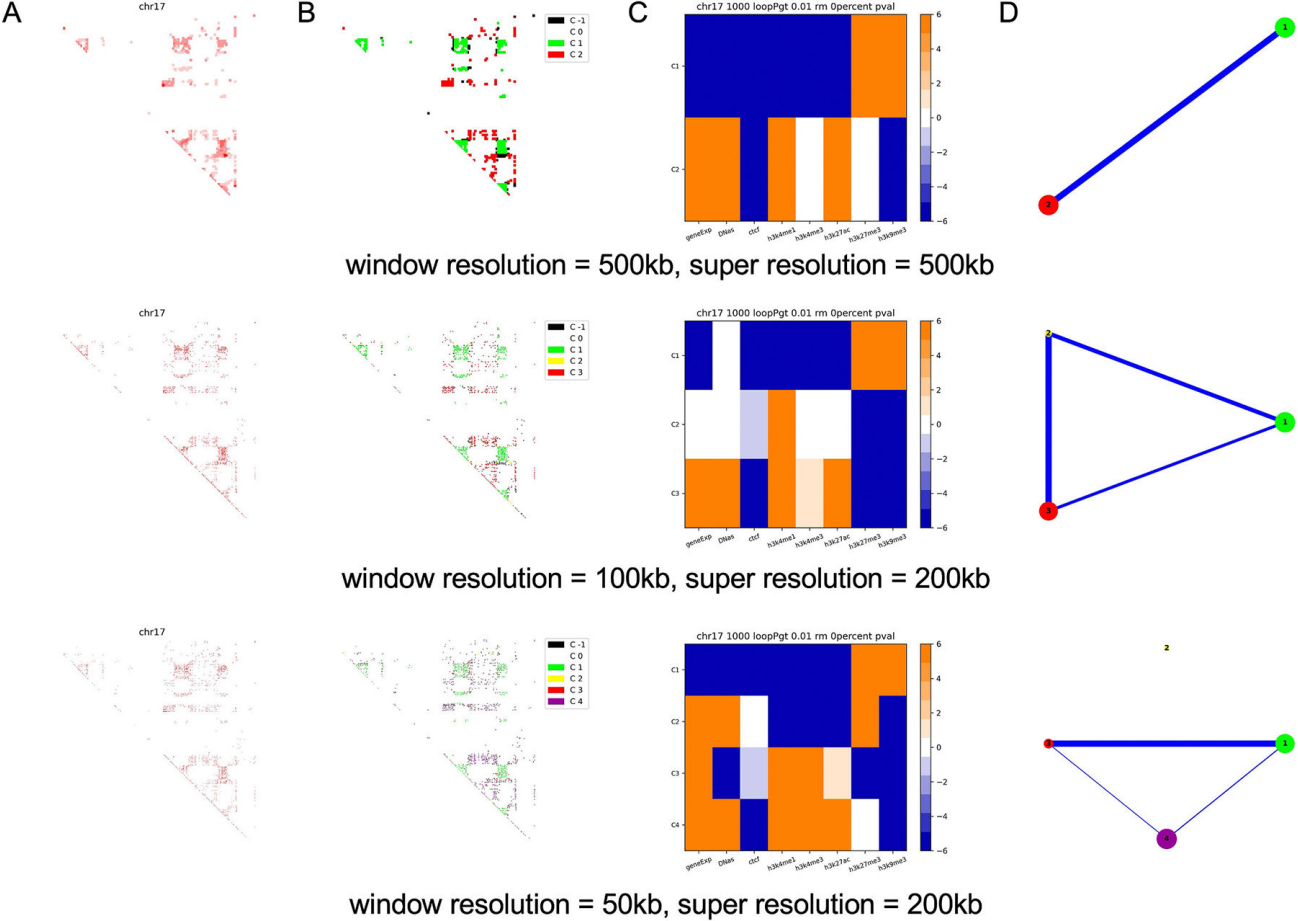
Heatmaps of intra-chromosomal community interactions for chromosomes 17 in three different resolutions based on significant intra-chromosomal interactions identified by HOMER in untreated MCF7 cells. The heatmaps of significant intra-chromosomal interactions identified by HOMER. (A) The heatmaps of color-coded community labels for intra-chromosomal interactions predicted by SLM algorithm, where the black and the other colors were a pair of interactions (the two nodes with an edge) classified in the different and the same community, respectively. (B) The heatmaps of genomic feature enrichments in valid communities, where the minimum size of valid communities was 23, 153, and 480 for window bin size equals 500 kb, 100 kb, and 50 kb, respectively. (C) Interactions of valid communities were represented by the super networks.

To identify significant interactions and to remove invalid communities in different interaction resolutions, new methods have been developed for fine-tuning HOMER parameters such as window resolution and super-resolution, as well as for estimating the minimum size of valid communities. Thus, the proposed analysis pipeline was repeated in different resolutions, by using only the predicted significant intra-chromosomal interactions with HOMER optimal parameters at window bin size equals 500 kb, 100 kb, 50 kb, respectively. For instance, to identify significant interactions at bin size 50 kb, the minimum size of valid communities was 480 (p=0.02; Supplementary Table S13) and the optimal HOMER window resolution and super resolution were 50 kb and 200 kb (Supplementary Fig. S12B), respectively. Fig. 6 and Supplementary Figs. S16 and S17 were the results of chromosomes 17, 3, and 20 in untreated MCF7 cells, respectively, where the significant intra-chromosomal interactions obtained from HOMER were consistent to each other in different resolutions such as heatmaps of Fig. 6A with bin size equals 500 kb, 100 kb, and 50 kb. Subsequently, communities of these significant interactions were predicted by SLM algorithm, and a common percentage cutoff value (p in Supplementary Table S13) was applied to remove invalid communities. For instance, if p=0.02 then the minimum size of valid communities was 23, 153, and 480 for 500 kb, 100 kb, and 50 kb interaction resolutions, respectively.

In Fig. 6B, the black and the other colors representing a pair of interactions (the two nodes with one edge) were clustered in different communities and the same community, respectively. In Fig. 6C, heatmaps of absolute log10 transformed *P*-values with sign of T-values were results of genomic feature enrichment tests in communities, which were obtained by a random permutation *t*-test (1000 times random sampling at a cutoff of expected P-value <0.01) for evaluating the significance of genomic features inside a community versus that outside the community. In the heatmaps, the orange and blue colors represented the positive and negative enrichment of genomic features in the communities, respectively. These results were similar to each other in different interaction resolutions such as bin size equals 500 kb, 100 kb, and 50 kb. For example, in chromosome 17, the intra-chromosomal interactions are mainly divided into two classes of communities at 500 kb resolution Fig. 6: one with low gene expression but high enrichment of repressive makers (H3K27me3 and H3K9me3), and the other with both high gene expression and high enrichment of activate markers (H3K4me1, H3K4me3, and H3K27ac). In the high resolutions (100 kb and 50 kb), there were more than two communities but they are also mainly divided into two clusters (the repressed or the activated genomic feature enrichment). This is consistent with known A/B compartments in the genome, which are associated with either euchromatin or heterochromatin [64]. Often, more detailed information of intra-chromosomal community interactions is revealed in the high resolution. For instance, an activated community C2 in 500 kb resolution (Fig. 6C) was divided into two communities in 100 kb resolution, and were further divided into three communities in 50 kb resolution. Though the two activated communities in 100 kb resolution (C2 and C3) are enriched differently by DNase and active markers (H3K4me3 and H3K27ac), they are similar in gene expression enrichment. The network graphs of these valid community interactions were shown in Fig. 6D, where the node size and the edge width indicate the community size and the number of community interactions, respectively. In summary, both the heatmaps of genomic feature enrichments and the network graphs of valid intra-chromosomal community interactions were consistent in different interaction resolutions (or window bin sizes).

For the other chromosomes in untreated, one-hour E2-treated MCF7 cells, and MCF7TR cells, similar results were obtained (Supplementary Figs. S16–23) by applying the newly developed parameter tuning methods on Hi-C data at different resolutions. In this way, both the predicted intra-chromosomal community interactions and the identified genomic feature enrichments in valid communities were consistent in different window bin sizes (e.g., 500 kb, 100 kb and 50 kb; Fig. 6). Thus, the results of high-resolution intra-chromosomal interactions (50 kb) were used further, for exploring the associations of dynamical changes in intra-chromosomal interactions with specific biological mechanisms.

### 3.6. Annotating genes involved in significant intra-chromosomal interaction changes

**Fig. 7.**
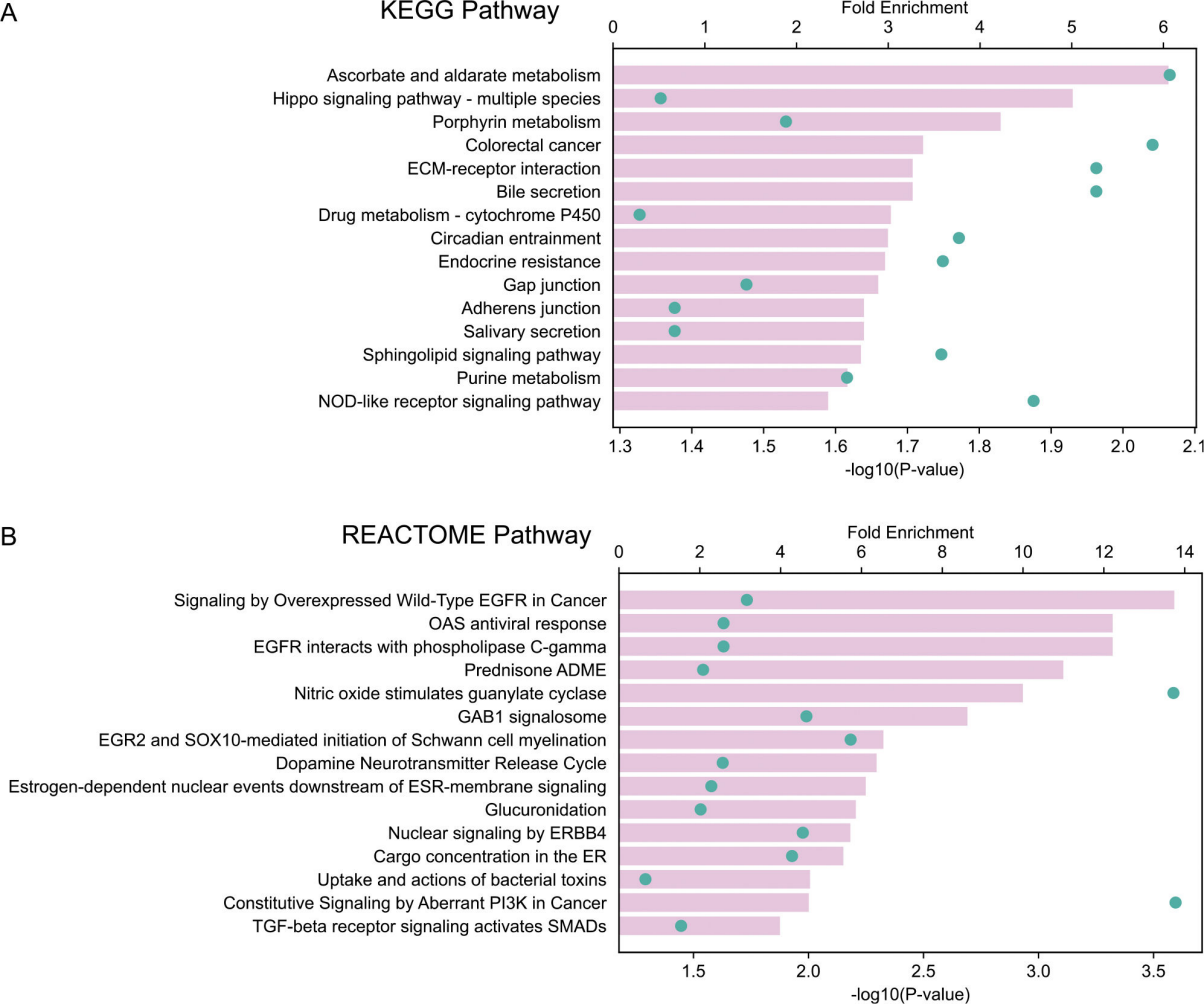
Top 15 enriched functional annotations of biological pathways on 515 DIEGs. Enrichment analysis of KEGG pathway and REACTOME pathway on 515 DIEGs obtained by DAVID online tool. The pink bar represents the Fold Enrichment (top axis), and the green scatters denote the −log10 transformed P-value (bottom axis). The top 15 functional annotations were selected under the thresholds of Fold Enrichment ≥ 2 and *p*-value ≤ 0.05, and ranked by Fold Enrichment. The 515 DIEGs were filtered through both differential network analysis and Absolute (relative ratio) > 0.66 from intra-chromosomal interaction networks between MCF7 cells and tamoxifen-resistant MCF7TR cells. (For interpretation of the references to color in this figure legend, the reader is referred to the web version of this article.)

We previously reported that differentially expressed looping genes, between MCF7 and MCF7TR, were associated with the oncogenesis pathway [27]. Here, a differential network analysis was performed, to identify significant Hi-C intra-chromosomal interaction changes between MCF7 cells and MCF7TR cells, at a resolution of 50 kb (window bin size = 50 kb). A total of 2681 nodes passed filtering criteria for significant Euclidean distance changes between the two conditions, such as the top 5 % (or an expected *p*-value<0.05 in a fitted Gaussian distribution for Euclidean distances; Supplementary Fig. S24) between the two conditions. In these nodes, there were 1378 genes (web Supplementary Files 1 and 2 at DNAICI webpage https://differential-network-analysis.github.io/dnaici-webpage/). After considering only differentially expressed genes (DEGs) (MCF7 vs. MCF7TR) with absolute(relative ratio) > 0.66 [26], the total number of selected genes were reduced to 515 that were involved in both the differential intra-chromosomal interaction and the differential expression (or DIEGs; web Supplementary File 3 at DNAICI webpage). Subsequently, significantly enriched gene ontology (GO) and biological pathways were identified by applying DAVID on these 515 DIEGs (Fig. 7, Supplementary Fig. S25, and web Supplementary File 4). Here, we identified several pathways with a notable fold enrichment from the analysis, which may contribute to tamoxifen-resistance. For example, metabolism pathways, e.g., ascorbate and aldarate metabolism, porphyrin metabolism, as well as sphingolipid signaling pathway, are enriched, which indicated altered cellular metabolism in resistant cells, aligning with findings from previous studies. [65,66]. Besides, the PI3K-Akt signaling pathway and the EGFR signaling pathway, recognized for promoting breast cancer cell survival and could provide alternative growth signals that negate tamoxifen’s effectiveness, exhibit enrichment. Furthermore, the mechanically gated and transmitter-gated ion channel activities were also found to be among the most significantly enriched GO terms, implicating that alteration of ion channels and transporters might play a role in contributing to endocrine-resistant breast cancer. Not surprisingly, we also detected endocrine resistance among the top pathways in KEGG, which further validated our method. Overall, the pathway enrichment test for DIEGs demonstrated looping-mediated mechanisms [19,28] in regulating tamoxifen resistant breast cancer.

### 3.7. Investigation of nodes involved in significant intra-chromosomal interaction changes

**Fig. 8.**
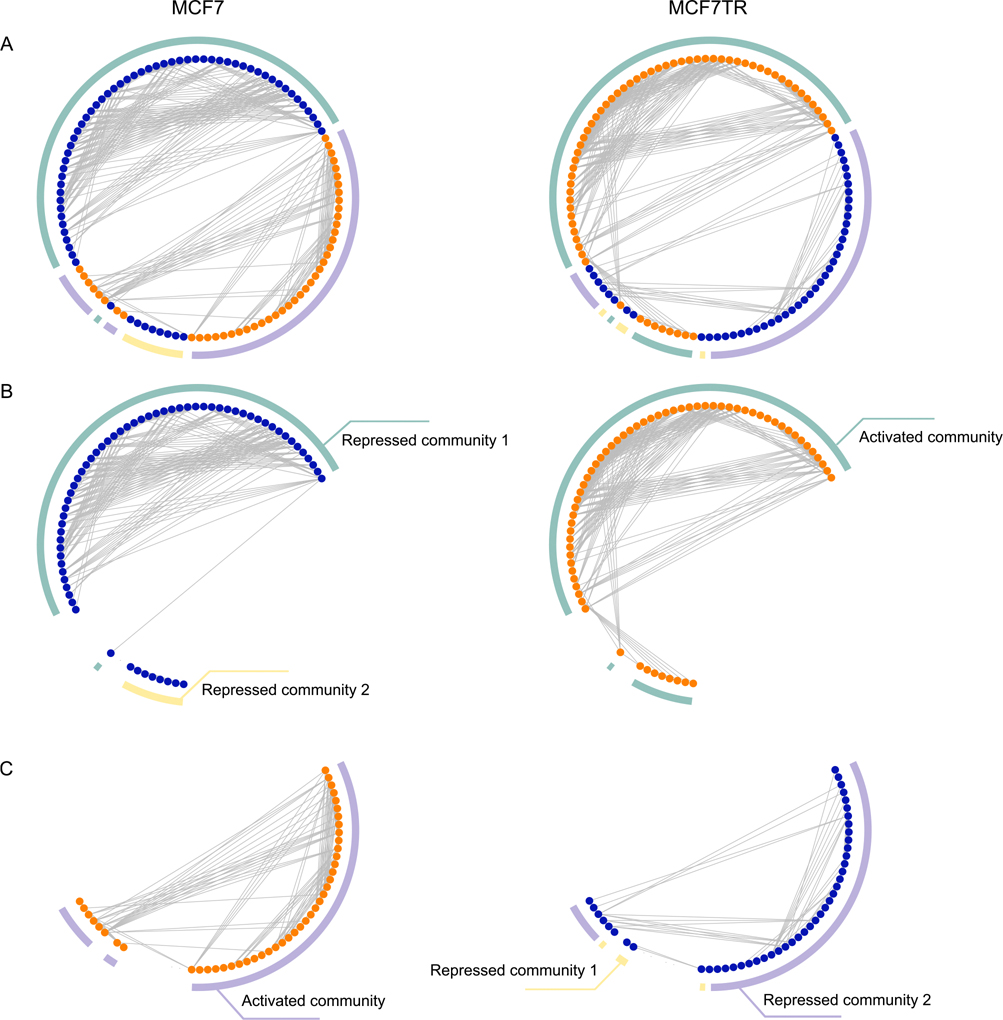
Differential sub-networks in chromosome 4 between MCF7 cells and MCF7TR cells. Sub-networks in chromosome 4 obtained by differential network analysis. Nodes in the network were selected based on DIEGs between MCF7 cells and MCF7TR cells, with each node representing a predefined window bin in chromosome (i.e., resolution = 50 kb). The orange and blue colors in the nodes represented whether the histone marks of the community were active or not. The colors of the outer rings represented previously identified intra-chromosomal communities. Gray lines between nodes represented significant Hi-C intra-chromosomal interactions. (A) Overview of differential networks in chromosome 4. (B) Subregion 1 of the chromosome shows different genomic features and intra-chromosomal interactions. (C) Subregion 2 of the chromosome shows different genomic features and intra-chromosomal interactions. (For interpretation of the references to color in this figure legend, the reader is referred to the web version of this article.)

For the selected 515 DIEGs, chromosome positions of the corresponding nodes (e.g., a node with a window bin size 50 kb) were extracted (web Supplementary Files 5 to 8), and the interactions of nodes in each chromosome under MCF7 cells (Fig. 8) and tamoxifen-resistant MCF7TR cells (web Supplementary File 9) were visualized by Cytoscape [67], respectively. In Fig. 8, the orange and blue node color represented active (enriched by H3K4me1 H3K4me3, and H3K27ac) and repressive communities (enriched by H3K27me3 and H3K9me3), respectively. If the nodes belong to the same community, then they are marked by the same color at the outer ring of the network. Also, if there are edges between the nodes, then they interact to each other at the corresponding condition such as the significant Hi-C intra-chromosomal interactions identified by HOMER. The differential network analysis between MCF7 and MCF7TR cells (Fig. 8 and web Supplementary File 9) indicated that not only the genomic feature enrichments in nodes were changed (Fig. 8A; from repressed to activated community), but also the nodes interactions were rewired (or intra-chromosomal interactions were changed) between the conditions. Thus, these predicted nodes from differential network analysis were involved in significant intra-chromosomal interaction changes and were potentially important in breast cancer gene regulation.

Especially, in chromosome 4, the genomic feature enrichment in selected nodes changed completely between MCF7 and MCF7TR cells: for example, two repressed communities (with green and yellow outer rings; left panel of Fig. 8B) in MCF7 become active communities in MCF7TR (right panel of Fig. 8B), and the interactions of nodes in repressed community 2 at MCF7 (yellow outer ring in left panel of Fig. 8B) were far less than that at MCF7TR (green outer ring in right panel of Fig. 8B). This was also true for the other communities, such as, the only activated community in MCF7 was split to two repressed communities in MCF7TR, while the node interactions were also changed significantly (Fig. 8C). Thus, genes in these selected nodes (chromosome 4; Fig. 8) were not only involved in significant intra-chromosomal interaction changes, but also might be responsible for the activation/repression of the region under different conditions. For example, CXXC4, PITX2, SLC16A10, TRAM1L1, NDST3, PRSS12, FAM184A, which normally play roles in maintaining cell proliferation, estrogen response and metabolism, were identified from the repressed community too (left panel of Fig. 8B). For instance, the downregulation of CXXC4 was reported as a contributing factor in tamoxifen resistance through the activation of Wnt/β-catenin pathway [68]. Conversely, 25 genes (e.g., CXCL2, CXCL3, CXCL8, detailed in web Supplementary File 10) were identified from the activated community (left panel of Fig. 8C) in the MCF7 condition. Specifically, the upregulation of CXCL2, CXCL3, and CXCL8 is positively associated with cancer metastasis and chemo-resistance. In contrast, downregulating these chemokines can significantly suppress cancer cell motility [69]. Besides, several studies reported that CXCL3 and CXCL8 played multiple roles within tumor microenvironment, including recruiting immunosuppressive cells to the tumor, increasing tumor angiogenesis, and promoting epithelial-to-mesenchymal transition (EMT), which are critical processes in tumor progression and metastasis [70,71].

### 3.8. Robustness of genomic feature enrichments in different mapping of Z-scores to Hi-C interaction matrix

**Fig. 9.**
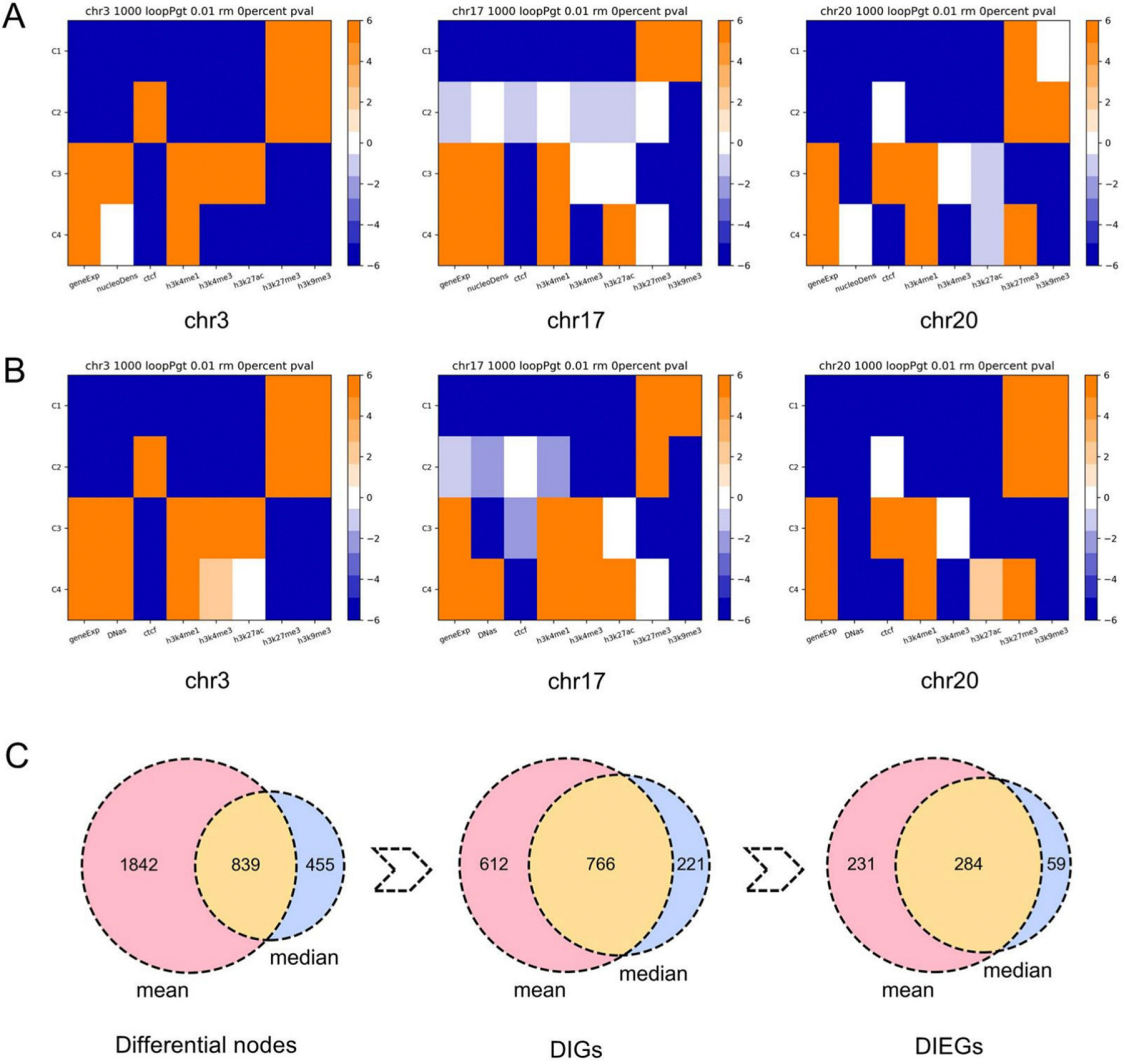
Comparisons of genomic feature enrichment and differential interaction between the median and the mean mapping of features to intra-chromosomal interaction matrix - window bin size 50 kb. (A) Heatmaps of genomics feature enrichments in valid communities from chromosomes 3, 17, and 20 in untreated MCF7 breast cancer cell lines. The genomic features were mapped to an intra-chromosomal interaction matrix with a median function. (B) Heatmaps of genomics feature enrichments in valid communities from chromosomes 3, 17, and 20 in untreated MCF7 breast cancer cell lines. The genomic features are mapped to an intra-chromosomal interaction matrix with a mean function. (C) Venn diagrams of differential nodes, differential interacting genes (DIGs), and differential interacting plus differentially expressed genes (DIEGs) when compared between the mean function and the median mapping of genomic features. All results were obtained at 50 kb resolution.

In the proposed integrated network analysis, genomic features are key to both the feature enrichment test and the differential network analysis. However, different calculations can be used to map genomic features to an adjacency matrix derived from Hi-C data, please refer to Supplementary Methods - Generating genomic feature matrices based on multi-omics data for Hi-C interactions and Supplementary Equations (7) and (8). To evaluate the robustness of results due to the *Z*-score calculations in interaction matrix, DNAICI was applied on the same datasets based on two types of calculations (the mean or the median of *Z*-scores in a window bin) in adjacency matrices with 50 kb and 500 kb resolutions, respectively. Fig. 9A and B showed the results of genomic enrichment heatmaps in intra-chromosomal interaction communities (chr3, chr17, and chr20; window bin size = 50 kb) based on the median and the mean of *Z*-scores, respectively. Overall, the heatmaps of genomic feature enrichments in communities were similar between the median and the mean of *Z*-scores (Fig. 9A and B). For example, in the left panel of Fig. 9A and B (chr3), four communities were classified into two major clusters based on the genomic feature enrichment: 1) activated communities (C3 and C4) with positive enrichment of gene expression and enhancer/promoter makers (H3K4me1, H3K4me3, and H3K27ac); 2) repressed communities (C1 and C2) with negative enrichment of enhancer/promoter makers but have positive enrichment of repression markers (H3K27me3 and H3K9me3). It is also true for genomic enrichment heatmaps of chr17 and chr20 such as the middle and the right panels of Fig. 9A and B for the median and the mean of *Z*-scores, respectively.

Additionally, RV-coefficient [72] was used to assess the similarity between the heatmaps (Fig. 9A vs. Fig. 9B). Please refer to Supplementary Table S14 for pair-wise comparisons of heatmaps between the two calculations in all chromosomes. An average of RV-coefficients equals ~0.8 indicated that the two genomic feature enrichment heatmaps/matrices were very similar, please refer to Supplementary Table S14 for results at 50 kb interaction resolution. Furthermore, the nodes of intra-chromosomal interactions and the genes obtained from differential network analysis (the top 5 % of Euclidean distance changes), between the two calculations of Z-scores in Hi-C interactions (or adjacency matrices), were also compared. The left most Venn diagram of Fig. 9C shows ~839 of 1294 differential nodes (~60 %) from the median Z-scores calculation are overlapping to that from the mean ones. After considering only genes located in the differential nodes (DIGs), the middle Venn diagram of Fig. 9C indicated that ~766 of 987 genes (~80 %) obtained from the median Z-scores calculation were overlapping with those from the mean ones. When the comparison was further narrowed down to the differentially expressed genes (DIEGs), the right most Venn diagram of Fig. 9C showed that ~284 of 343 genes (~85 %) were common in both results. Taken together, the genomic enrichment heatmaps and the DIEGs, obtained from the two different Z-scores calculations in the Hi-C interaction matrix, were similar. In particular, the genes obtained by DNAICI from differential network analysis were robust, regardless of using the mean or the median of Z-scores for a window bin.

### 3.9. Biological relevance of diagonal elements in intra-chromosomal interaction matrix

**Fig. 10.**
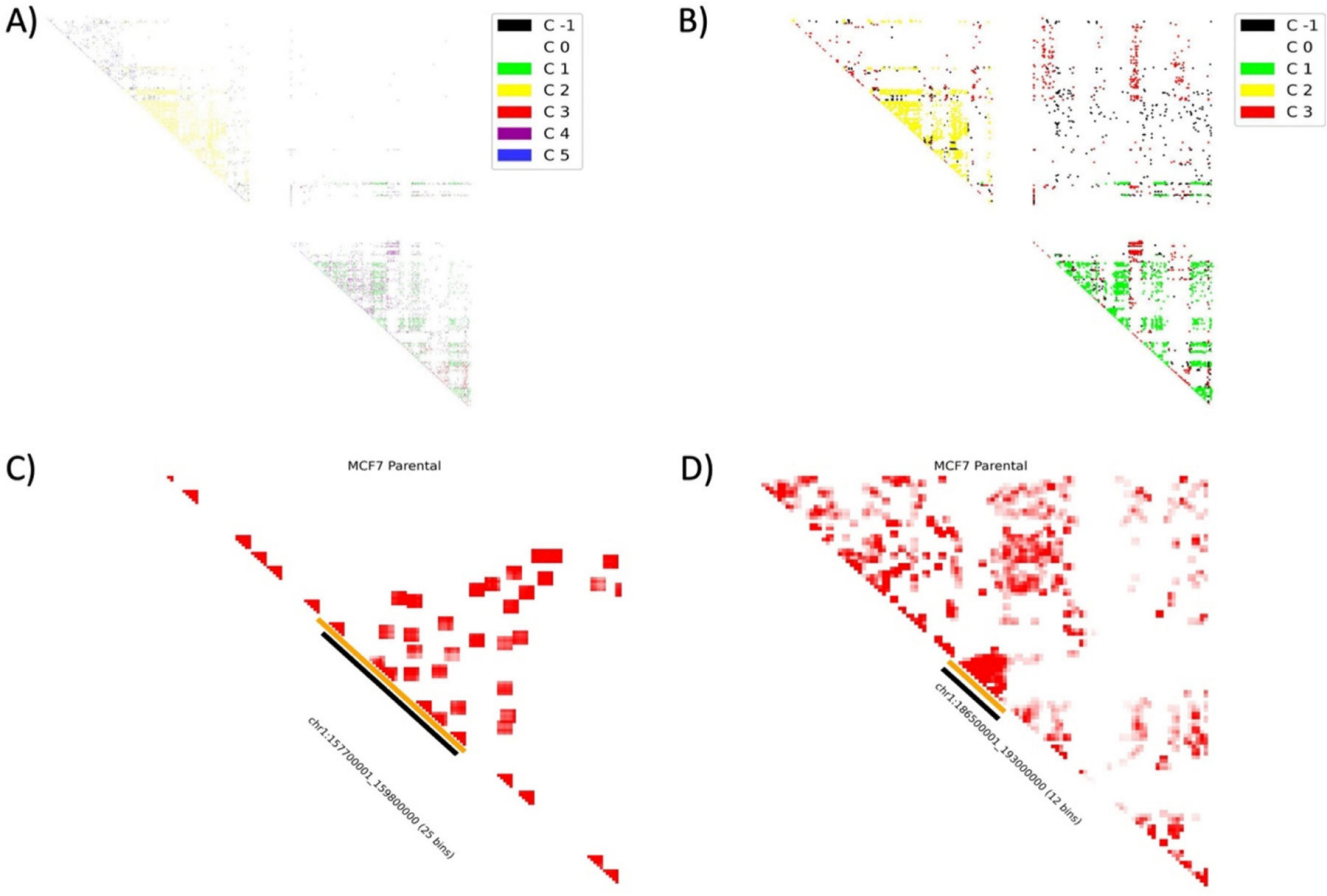
Heatmaps of intra-chromosomal community interactions for chromosome 1 at 50 kb and 500 kb resolution in untreated MCF7 cells. Fig. A) and B) show heatmaps of identified valid network communities (or clusters) of intra-chromosomal interactions at chromosome 1 with window bin size 50 kb and 500 kb, respectively. The results obtained by applying DNAICI on Hi-C datasets from untreated MCF7 cells. Fig. C) and D) display heatmaps of selected topologically associating domain (TAD) from chromosome 1 with window bin size 50 kb and 500 kb, respectively. The TAD was obtained by applying TopDom on the same intra-chromosomal interaction matrices in Figs. A) and B), respectively. In Figs. C) and D), the orange and black color bars indicated the position of the selected TAD and the diagonal window bins at valid network communities, respectively.

To investigate potential biological relevance of diagonal elements in Hi-C intra-chromosomal interaction matrix, DNAICI Python package with default parameters and window bin size 500 kb was applied on Hi-C data of MCF7 and MCF7TR cell lines, respectively, to identify significant Hi-C intra-chromosomal interactions (HOMER) and to predict network communities. The same analysis was repeated in the window bin size 50 kb. The minimum community size (or the number of nodes) of a network cluster (or a valid community) is 23 and 480 for intra-chromosomal interaction matrix (Supplementary Table 13) with window bin size 500 kb and 50 kb, respectively. Then, TopDom [73] was applied on the intra-chromosomal interaction matrices, for predicting both the topologically associating domain (TAD) and the Gap in MCF7 and MCF7TR cell lines, respectively. Here, the intra-chromosomal interaction matrices were normalized by Hi-Corrector [74] before using TopDom. Subsequently, window bins (or nodes) of valid network communities (the number of window bins > the minimum size of a valid community) from the main diagonal of the intra-chromosomal interaction matrix are compared with the TADs. In Fig. 10A and B, network clusters (or valid communities) of chr1 in MCF7 with window bin size (or interaction resolution) 50 kb and 500 kb were illustrated in heatmaps, respectively. The corresponding network clusters of chr1 in MCF7TR with window bin size 50 kb and 500 kb were displayed in Supplementary Figs. S26A and S26B, respectively. Examples of identified TADs of chr1 in MCF7 with window bin size 50 kb and 500 kb were illustrated in Fig. 10C and D, respectively. A TAD of chr1 in MCF7TR with window bin size 50 kb and 500 kb were shown in Supplementary Figs. S26C and S26D, respectively.

In the aforementioned TADs figures, the orange and black color bars represent the TAD position and the corresponding diagonal elements (or window bins in valid network communities/clusters) of the intra-chromosomal interaction matrix, respectively. By counting the number of TADs in each chromosome predicted by TopDom, we found >90 % of TADs (50 kb and 500 kb; Supplementary Figs. S27A and S27B) are overlapping with the diagonal elements of interaction matrices, which are also located in valid network communities (or clusters; MCF7 and MCF7TR in Supplementary Fig. S27). In the other words, the majority of TADs are overlapping with the diagonal elements of the intra-chromosomal interaction matrix that are positioned in valid communities. However, the number of diagonal elements (or window bins) from valid communities that overlap with the Gap is much smaller, and it is highly dependent on the interaction resolution (or window bin size). For example, in Supplementary Fig. S27, ~30 % and 60 % of the predicted Gaps were intersected with the diagonal elements from valid network communities at window bins size 50 kb and 500 kb, respectively. Thus, the higher the interaction resolution the smaller the number of diagonal elements in the Gap. On the contrary, there was a stable number of TADs overlapping with the diagonal elements from valid network clusters (>90 %; Supplementary Fig. S27) regardless of the interaction resolution. In summary, the diagonal elements of the intra-chromosomal interaction matrix were often in the same TAD, if they were in the same network cluster (or valid community) of 3D-genome regulation.

### 3.10. Comparison of inferred network clusters (or valid communities) with A/B compartments

Though intra-chromosomal interactions obtained from Hi-C data are dense and complicated to perform biological interpretation, high dimensional intra-chromosomal interactions can be summarized to low dimensional subgroups such as A/B compartments for a close study [19]. To identify A/B compartments from Hi-C data, it often converts intra-chromosomal interaction matrices to correlation coefficient matrices before applying the principal component analysis (PCA). The first PC contains the most informative values, which can be used to classify chromosome regions based on intra-chromosomal interactions from Hi-C data [43]. A classification of chromosome regions to A/B compartments is in analogy to the network clustering (or the identification of valid community interactions) of intra-chromosomal interactions. That is because both approaches are trying to reduce the high dimensional interaction space to the low dimensional subgroup space such as compartments or communities. It is worthy to compare A/B compartments with valid communities because the former ones are already well recognized in 3D genome regulation as the active/repressive chromosome regions [19]. By using the same intra-chromosomal interaction matrices obtained from the earlier TAD analysis in MCF7 and MCF7TR cells, both HOMER [43] and FAN-C [75] were used to predict A/B compartments in two different interaction resolutions such as 50 kb and 500 kb window bin size, respectively. Here, HOMER was applied on the HOMER identified significant intra-chromosomal interactions (*P*-value <0.1), but FAN-C was applied on the full intra-chromosomal interactions derived from the Hi-C data. Then, DNAICI was used to identify valid intra-chromosomal community interactions in 50 kb and 500 kb interaction resolution, respectively, based on the significant interactions used by HOMER. Subsequently, a comparison between the predicted A/B compartments and the detected network clusters (or valid intra-chromosomal interaction communities) was performed.

**Fig. 11.**
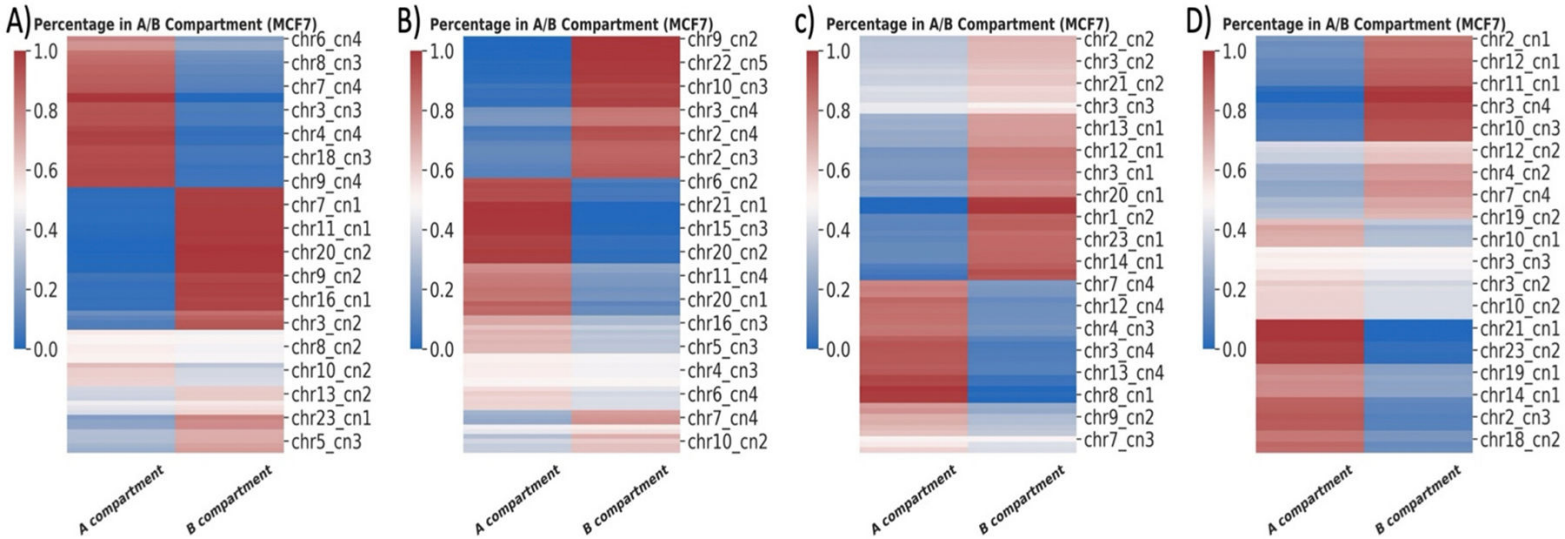
A comparison of window bins in intra-chromosomal communities and A/B compartments in untreated MCF7 cells. Figs. A) and B) showed heatmaps of percentages of window bins (50 kb resolution) in valid intra-chromosomal communities (MCF7 cells) belonged to predicted A/B compartments by HOMER [43] and FAN-C [75], respectively. Figs. C) and D) were heatmaps of percentages of window bins (500 kb resolution) in valid intra-chromosomal communities (MCF7 cells) belonging to predicted A/B compartments by HOMER and FAN-C, respectively. The valid intra-chromosomal community interactions were identified by applying DNAICI on Hi-C data from untreated MCF7 cells at 50 kb and 500 kb resolution, respectively.

In Fig. 11A and B, the percentages of window bins in a valid intra-chromosomal interaction community overlap with A/B compartment are illustrated by heatmaps at 50 kb interaction resolution. Here, the A/B compartments were predicted by HOMER and FAN-C in untreated MCF7 cells, respectively. In Fig. 11C and D, heatmaps showed the corresponding results at 500 kb interaction resolution in untreated MCF7 cells. These results suggested that there were three major types of intra-chromosome interaction communities: 1) network clusters (or valid communities) with a high percentage (>80 %) of window bins (or chromosome regions) in A compartment – Active Genomic Zones; 2) network clusters (or communities) with a high percentage of window bins (>80 %) in B compartment – Inactive Genomic Zones; 3) network clusters (or communities) with a marginal percentage of window bins in either A or B compartments (~50 %) - Poised Genomic Zones. These findings are consistent in results obtained from both 50 kb (Supplementary Figs. S28A, S28B) and 500 kb interaction resolutions (Supplementary Figs. S28C, S28D) in MCF7TR. Though the current finding of three types of genomic zones (Active, Inactive, Poised) in intra-chromosomal interactions are solely dependent on the network structure of Hi-C data in breast cancer cell lines, it is consistent with previous results (three types of functional regulatory regions in IMR90, K562 and GM12878 cell lines) [48,76] obtained by integrative analysis of multiple-omics datasets (e.g., gene expression, CTCF binding, histone modifications of active and repressive marks, nucleosome density et al) in the other human cell lines. Thus, the intra-chromosomal interactions (or the topology of network structures) from Hi-C data were not randomly distributed, but closely following their potential functions (active, inactive, or poised) in regulatory regions.

## 4. Discussion and conclusions

With emerging sequencing technologies [16,77,78], a systematical multi-modal approach to integrate epigenetic data is demanded. In this work, an epigenetic data integration approach (Fig. 1) was developed for differential network analysis in intra-chromosomal interactions. Our work optimized and solved three difficulties in the field. First, there is neither a proper method to find optimal interaction resolution nor a good solution to estimate optimal parameters. In this case, chromosomal interaction networks are frequently affected by the sequencing depth of Hi-C data that sequentially affect the degree of Hi-C interaction resolution [79]. In DNAICI, we developed a novel approach (Supplementary Fig. S12) for fine-tuning key parameters—namely window resolution and super-resolution—across varying interaction scales, to robustly identify significant intra-chromosomal interactions. This approach ensures consistency in the inferred interaction networks across different resolutions (e.g., bin sizes of 500 kb, 100 kb, and 50 kb; Supplementary Fig. S6 and Fig. 6). Consequently, a high-resolution intra-chromosomal interaction network (e.g., 50 kb bin size) can be reliably constructed from Hi-C data to enable more detailed network analyses.

Secondly, intra-chromosomal interaction networks inferred from Hi-C data often contain a huge number of interactions such as tens of thousands of interactions with a window bin size 500 kb (Figs. 2 and 3). This hinders the data analysis and biological interpretation of chromosomal interactions. To tackle this problem, we applied a dimensionality reduction strategy by leveraging network clustering—specifically, community detection using the SLM algorithm with a local moving heuristic—to condense high-dimensional intra-chromosomal interactions into low-dimensional community interactions (Figs. 4 and 5). In this framework, each predicted community (or super node) comprises multiple nodes from the original interaction network. We applied SLM, which has been proven for successfully clustering on a network, with adaptation for finding optimal size of valid communities in a specific interaction resolution in DNAICI. Usually, the SLM algorithm has more freedom than the other methods (e.g., Louvain algorithm [56]) in searching for high quality community structure because of an optimization of modularity score function. Particularly, if a modularity score reaches 0.3 then there is a significant community structure in the interaction network [55]. Nevertheless, the SLM algorithm frequently generates many small communities (or network clusters) when clustering a large network, due to either experimental error or computational bias. Thus, we designed a new statistical method (minimum size of valid communities) in DNAICI to handle such problems in the network clustering.

Thirdly, we performed differential network analysis by bridging between the intra-chromosomal community interactions and the genomic feature enrichment in the communities, where network nodes involved in significant intra-chromosomal interaction changes (e.g., MCF7 vs. MCF7TR) are identified. Genes associated with these significantly changed nodes were not only enriched in GO and pathways consistent with existing biological perspectives, such as the presence of looping-mediated mechanisms in endocrine-resistant breast cancer (Fig. 7), but also potentially related to conditions that caused the physical and functional changes of intra-chromosomal interactions (Fig. 8). Additionally, we found that the diagonal elements (or window bins) of an intra-chromosomal interaction matrix were often located at the same TAD, when they were clustered in the same network community. The distribution of intra-chromosomal interactions (or the network structure of valid community interactions) is not random, but follows a functional specific (active, inactive or poised) network topology in 3D genome regulation, after comparing the identified valid communities with the predicted A/B compartments. The current finding of three types of genomic regions in the breast cancer cell lines from Hi-C data only, is being consistent to the previous results based on an integrative multi-omics data analysis (e.g., gene expression, nucleosome density, and histone modifications et al.) in the other human cell lines [48,76]. In other words, if chromosome regions share a similar biologically functional state (e.g., active, inactive, or poised genomic zones), then they are often being grouped together in the same intra-chromosomal interaction community (or network cluster). These new results suggest that the proposed integrated analysis of differential networks, in intra-chromosomal community interactions (Fig. 1), facilitates understanding of both mechanism and structure of long-distance gene regulation based on chromatin loops [7]. Our approach (the community interaction network) not only significantly reduces the size of intra-chromosomal interactions, but also helps tremendously for understanding the property of network structures in a large intra-chromosomal interaction network [55,80] when considering diverse genomic features.

Though our method has successfully addressed several issues in Hi-C data analysis, there are a few areas that require further improvement in the future. In the current research, we focused solely on differentially expressed genes from significantly altered nodes in the differential network analysis (intra-chromosomal interaction). This approach may overlook some other interesting aspects of 3D gene regulation (e.g., inter-chromosomal interactions). A second limitation is the substantial computational demand for the proposed integrative network analysis, especially when analyzing high-resolution interactions such as window bin size <50 kb. This requirement can impede the study of differential interactions influenced by a range of genomic features such as histone modifications, nucleosome density, and gene expression. Although the DNAICI Python package incorporates parallel computation, it still requires a considerable amount of CPU time to perform random permutation tests for evaluating genomic feature enrichments in interaction communities. Consequently, there is a need to speed up the calculation in high-resolution interactions, such as bin size <20 kb that is currently impractical, for handling genomic feature enrichment tests. Lastly, the success of computational predictions largely depends on the quality and quantity of the input datasets that are used for training the method. For breast cancer cell lines like MCF7, numerous Hi-C data and epigenetic data are publicly available, and this ERα + breast cancer model system has been extensively studied in many biomedical research projects. However, for other biological systems and human diseases, including cancer, multi-omics datasets are not only costly to produce and scarce in the public domain, but high-quality data are also challenging to obtain from limited biopsy samples [81–84]. These challenges could significantly impact the application of the proposed integrated network analysis in future studies of differential interactions.

Breast cancer is the most common cancer in women in the United States [85] and around half of the patients relapse after initial endocrine treatment [86]. In this study, we used two breast cancer cell lines, including breast cancer parental MCF7 and corresponded tamoxifen-resistant MCF7TR, as the example study case for DNAICI. By applying DNAICI, we discovered active-repressive transition regions between MCF7 and MCF7TR. These changes in chromatin interaction landscapes were correlated with the gene expression profiles observed, where genes within these dynamically altered regions display differential expression linked to tamoxifen resistance. For instance, the analysis identified specific pathways such as PI3K-Akt and EGFR signaling, known for promoting cancer cell survival and recapitulated previous reports [87,88]. Furthermore, the analysis highlighted changes in metabolic pathways and ion channel activities, which were increasingly recognized as critical players in cancer progression and therapy resistance [89,90]. These findings underscore the potential of our DNAICI approach not only to map the complex network of chromosomal interactions but also to link these structural changes to functional outcomes, offering a powerful tool for dissecting the molecular underpinnings of disease states like endocrine-resistant breast cancer. This integration of high-resolution chromatin interaction data with gene expression and other genomic features, opens new avenues for targeted therapeutic strategies and enhances our ability to predict disease progression and treatment response.

In conclusion, an integrated network analysis approach was developed for identifying significant intra-chromosomal interaction changes, by combining Hi-C data with other genomic features such as active/repressive histone modifications, gene expression, and nucleosome densities etc. And novel parameter tuning methods were designed to find a proper intra-chromosomal interaction resolution, and to estimate an optimal community size under a specific Hi-C experiment. The proposed network analysis framework - DNAICI was evaluated in breast cancer cell lines such as untreated or one-hours-E2-treated MCF7 cells and MCF7TR cells, from which a comprehensive picture of chromatin interactions from Mb-sized intra-chromosomal communities to Kb-sized foci of key interactions was provided. The genes of nodes associated with significant intra-chromosomal interaction changes (MCF7 vs. MCF7TR), are not only enriched in GO and pathways consistent with existing biological perspectives, but also providing methodological and mechanistic insights for future research, such as to explore significant genomic dynamics in cancer and beyond by integrating multi-omics datasets.

## Supplementary Material

1

## Appendix A. Supplementary data

Supplementary data to this article can be found online at https://doi.org/10.1016/j.artmed.2025.103180.

## References

[1] Lieberman-Aiden E, van Berkum NL, Williams L, Imakaev M, Ragoczy T, Telling A, Comprehensive mapping of long-range interactions reveals folding principles of the human genome. Science 2009;326(5950):289–93. 19815776 10.1126/science.1181369 PMC2858594

[2] Dixon JR, Selvaraj S, Yue F, Kim A, Li Y, Shen Y, Topological domains in mammalian genomes identified by analysis of chromatin interactions. Nature 2012;485(7398):376–80. 22495300 10.1038/nature11082 PMC3356448

[3] Fullwood MJ, Liu MH, Pan YF, Liu J, Xu H, Mohamed YB, An oestrogen-receptor-alpha-bound human chromatin interactome. Nature 2009;462(7269):58–64. 19890323 10.1038/nature08497 PMC2774924

[4] Jin F, Li Y, Dixon JR, Selvaraj S, Ye Z, Lee AY, A high-resolution map of the three-dimensional chromatin interactome in human cells. Nature 2013;503(7475):290–4. 24141950 10.1038/nature12644 PMC3838900

[5] Kalhor R, Tjong H, Jayathilaka N, Alber F, Chen L. Genome architectures revealed by tethered chromosome conformation capture and population-based modeling. Nat Biotechnol 2011;30(1):90–8. 22198700 10.1038/nbt.2057 PMC3782096

[6] Yaffe E, Tanay A. Probabilistic modeling of Hi-C contact maps eliminates systematic biases to characterize global chromosomal architecture. Nat Genet 2011;43(11):1059–65. 22001755 10.1038/ng.947

[7] Rao SSP, Huntley MH, Durand NC, Stamenova EK, Bochkov ID, Robinson JT, A 3D map of the human genome at kilobase resolution reveals principles of chromatin looping. Cell 2014;159(7):1665–80. 25497547 10.1016/j.cell.2014.11.021 PMC5635824

[8] Ling JQ, Li T, Hu JF, Vu TH, Chen HL, Qiu XW, CTCF mediates interchromosomal colocalization between Igf2/H19 and Wsb1/Nf1. Science 2006;312(5771):269–72. 16614224 10.1126/science.1123191

[9] Hsu PY, Hsu HK, Lan X, Juan L, Yan PS, Labanowska J, Amplification of distant estrogen response elements deregulates target genes associated with tamoxifen resistance in breast cancer. Cancer Cell 2013;24(2):197–212. 23948299 10.1016/j.ccr.2013.07.007 PMC3890247

[10] Nora EP, Goloborodko A, Valton AL, Gibcus JH, Uebersohn A, Abdennur N, Targeted degradation of CTCF decouples local insulation of chromosome domains from genomic compartmentalization. Cell 2017;169(5):930–44. e922. 28525758 10.1016/j.cell.2017.05.004 PMC5538188

[11] Bulger M, Groudine M. Functional and mechanistic diversity of distal transcription enhancers. Cell 2011;144(3):327–39. 21295696 10.1016/j.cell.2011.01.024 PMC3742076

[12] Dekker J, Mirny L. The 3D genome as moderator of chromosomal communication. Cell 2016;164(6):1110–21. 26967279 10.1016/j.cell.2016.02.007 PMC4788811

[13] Schoenfelder S, Fraser P. Long-range enhancer-promoter contacts in gene expression control. Nat Rev Genet 2019;20(8):437–55. 31086298 10.1038/s41576-019-0128-0

[14] Duan Z, Andronescu M, Schutz K, McIlwain S, Kim YJ, Lee C, A three-dimensional model of the yeast genome. Nature 2010;465(7296):363–7. 20436457 10.1038/nature08973 PMC2874121

[15] Belton JM, McCord RP, Gibcus JH, Naumova N, Zhan Y, Dekker J. Hi-C: a comprehensive technique to capture the conformation of genomes. Methods 2012;58(3):268–76. 22652625 10.1016/j.ymeth.2012.05.001 PMC3874846

[16] van Berkum NL, Lieberman-Aiden E, Williams L, Imakaev M, Gnirke A, Mirny LA, Hi-c: a method to study the three-dimensional architecture of genomes. J Vis Exp 2010;39. 10.3791/1869 PMC3149993 20461051

[17] Boulos RE, Arneodo A, Jensen P, Audit B. Revealing long-range interconnected hubs in human chromatin interaction data using graph theory. Phys Rev Lett 2013;111(11):118102. 24074120 10.1103/PhysRevLett.111.118102

[18] Sandhu KS, Li G, Poh HM, Quek YL, Sia YY, Peh SQ, Large-scale functional organization of long-range chromatin interaction networks. Cell Rep 2012;2(5):1207–19. 23103170 10.1016/j.celrep.2012.09.022 PMC4181841

[19] Zhou Y, Gerrard DL, Wang J, Li T, Yang Y, Fritz AJ, Temporal dynamic reorganization of 3D chromatin architecture in hormone-induced breast cancer and endocrine resistance. Nat Commun 2019;10(1):1522. 30944316 10.1038/s41467-019-09320-9 PMC6447566

[20] Franke M, Ibrahim DM, Andrey G, Schwarzer W, Heinrich V, Schopflin R, Formation of new chromatin domains determines pathogenicity of genomic duplications. Nature 2016;538(7624):265–9. 27706140 10.1038/nature19800

[21] Ji X, Dadon DB, Powell BE, Fan ZP, Borges-Rivera D, Shachar S, 3D chromosome regulatory landscape of human pluripotent cells. Cell Stem Cell 2016;18(2):262–75. 26686465 10.1016/j.stem.2015.11.007 PMC4848748

[22] Bonev B, Cavalli G. Organization and function of the 3D genome. Nat Rev Genet 2016;17(12):772. 28704353 10.1038/nrg.2016.147

[23] Dekker J, Marti-Renom MA, Mirny LA. Exploring the three-dimensional organization of genomes: interpreting chromatin interaction data. Nat Rev Genet 2013;14(6):390–403. 23657480 10.1038/nrg3454 PMC3874835

[24] Zhou Y, Cheng X, Yang Y, Li T, Li J, Huang TH, Modeling and analysis of Hi-C data by HiSIF identifies characteristic promoter-distal loops. Genome Med 2020;12(1):69. 32787954 10.1186/s13073-020-00769-8 PMC7425017

[25] Lan X, Witt H, Katsumura K, Ye Z, Wang Q, Bresnick EH, Integration of Hi-C and ChIP-seq data reveals distinct types of chromatin linkages. Nucleic Acids Res 2012;40(16):7690–704. 22675074 10.1093/nar/gks501 PMC3439894

[26] Wang J, Lan X, Hsu PY, Hsu HK, Huang K, Parvin J, Genome-wide analysis uncovers high frequency, strong differential chromosomal interactions and their associated epigenetic patterns in E2-mediated gene regulation. BMC Genomics 2013;14(70). 10.1186/1471-2164-14-70 PMC3599885 23368971

[27] Yang YN, Choppavarapu L, Fang K, Naeini AS, Nosirov B, Li JW, The 3D genomic landscape of differential response to EGFR/HER2 inhibition in endocrine-resistant breast cancer cells. Biochim Biophys Acta 2020;1863(11). 10.1016/j.bbagrm.2020.194631 PMC7686120 32956836

[28] Li J, Fang K, Choppavarapu L, Yang K, Yang Y, Wang J, Hi-C profiling of cancer spheroids identifies 3D-growth-specific chromatin interactions in breast cancer endocrine resistance. Clin Epigenetics 2021;13(1):175. 34535185 10.1186/s13148-021-01167-6 PMC8447690

[29] Nicoletti C Methods for the differential analysis of Hi-C data. Methods Mol Biol 2022;2301:61–95. 34415531 10.1007/978-1-0716-1390-0_4

[30] Hua D, Gu M, Zhang X, Du Y, Xie H, Qi L, DiffDomain enables identification of structurally reorganized topologically associating domains. Nat Commun 2024;15(1):502. 38218905 10.1038/s41467-024-44782-6 PMC10787792

[31] Stansfield JC, Cresswell KG, Vladimirov VI, Dozmorov MG. HiCcompare: an R-package for joint normalization and comparison of HI-C datasets. BMC Bioinformatics 2018;19(1):279. 30064362 10.1186/s12859-018-2288-x PMC6069782

[32] Chakraborty A, Wang JG, Ay F. dcHiC detects differential compartments across multiple Hi-C datasets. Nat Commun 2022;13(1):6827. 36369226 10.1038/s41467-022-34626-6 PMC9652325

[33] Liu H, Ma W. DiffGR: detecting differentially interacting genomic regions from Hi-C contact maps. Genomics Proteomics Bioinformatics 2024;22(2):qzae028. 39222712 10.1093/gpbjnl/qzae028 PMC12016564

[34] Robinson MD, McCarthy DJ, Smyth GK. edgeR: a Bioconductor package for differential expression analysis of digital gene expression data. Bioinformatics 2010;26(1):139–40. 19910308 10.1093/bioinformatics/btp616 PMC2796818

[35] Lun AT, Smyth GK. diffHic: a Bioconductor package to detect differential genomic interactions in hi-C data. BMC Bioinformatics 2015;16(258). 10.1186/s12859-015-0683-0 PMC4539688 26283514

[36] Jin VX, Leu YW, Liyanarachchi S, Sun H, Fan M, Nephew KP, Identifying estrogen receptor alpha target genes using integrated computational genomics and chromatin immunoprecipitation microarray. Nucleic Acids Res 2004;32(22):6627–35. 15608294 10.1093/nar/gkh1005 PMC545447

[37] Cheng ASL, Jin VX, Fan MY, Smith LT, Liyanarachchi S, Yan PS, Combinatorial analysis of transcription factor partners reveals recruitment of c-MYC to estrogen receptor-α responsive promoters. Mol Cell 2006;21(3):393–404. 16455494 10.1016/j.molcel.2005.12.016

[38] Gu F, Hsu HK, Hsu PY, Wu JJ, Ma YL, Parvin J, Inference of hierarchical regulatory network of estrogen-dependent breast cancer through ChIP-based data. BMC Syst Biol 2010;4. 10.1186/1752-0509-4-170 PMC3012048 21167036

[39] Jin VX, Sun H, Pohar TT, Liyanarachchi S, Palaniswarny SK, Huang THM, ERtargetDB: an integral information resource of transcription regulation of estrogen receptor target genes. J Mol Endocrinol 2005;35(2):225–30. 16216904 10.1677/jme.1.01839

[40] Hsu PY, Hsu HK, Hsiao TH, Ye Z, Wang E, Profit AL, Spatiotemporal control of estrogen-responsive transcription in ERα-positive breast cancer cells. Oncogene 2016;35(18):2379–89. 26300005 10.1038/onc.2015.298 PMC4865474

[41] Wingett S, Ewels P, Furlan-Magaril M, Nagano T, Schoenfelder S, Fraser P, HiCUP: pipeline for mapping and processing Hi-C data. F1000Res 2015;4:1310. 26835000 10.12688/f1000research.7334.1 PMC4706059

[42] Bau D, Sanyal A, Lajoie BR, Capriotti E, Byron M, Lawrence JB, The three-dimensional folding of the alpha-globin gene domain reveals formation of chromatin globules. Nat Struct Mol Biol 2011;18(1):107. 21131981 10.1038/nsmb.1936 PMC3056208

[43] Heinz S, Benner C, Spann N, Bertolino E, Lin YC, Laslo P, Simple combinations of lineage-determining transcription factors prime cis-regulatory elements required for macrophage and B cell identities. Mol Cell 2010;38(4):576–89. 20513432 10.1016/j.molcel.2010.05.004 PMC2898526

[44] Cicatiello L, Mutarelli M, Grober OMV, Paris O, Ferraro L, Ravo M, Estrogen receptor alpha controls a gene network in luminal-like breast cancer cells comprising multiple transcription factors and MicroRNAs. Am J Pathol 2010;176(5):2113–30. 20348243 10.2353/ajpath.2010.090837 PMC2861078

[45] Bi M, Zhang Z, Jiang YZ, Xue P, Wang H, Lai Z, Enhancer reprogramming driven by high-order assemblies of transcription factors promotes phenotypic plasticity and breast cancer endocrine resistance. Nat Cell Biol 2020;22(6):701–15. 32424275 10.1038/s41556-020-0514-z PMC7737911

[46] Zhao YX, Wong, Goh WWB. How to do quantile normalization correctly for gene expression data analyses. Sci Rep 2020;10(1). 10.1038/s41598-020-72664-6 PMC7511327 32968196

[47] Dunham I, Kundaje A, Aldred SF, Collins PJ, Davis C, Doyle F, An integrated encyclopedia of DNA elements in the human genome. Nature 2012;489(7414):57–74. 22955616 10.1038/nature11247 PMC3439153

[48] Naeini AS, Farooq A, Bjoras M, Wang JB. IGAP-integrative genome analysis pipeline reveals new gene regulatory model associated with nonspecific TF-DNA binding affinity. Comput Struct Biotechnol J 2020;18:1270–86. 32612751 10.1016/j.csbj.2020.05.024 PMC7303559

[49] Langmead B, Salzberg SL. Fast gapped-read alignment with bowtie 2. Nat Methods 2012;9(4):357–U354. 22388286 10.1038/nmeth.1923 PMC3322381

[50] Zhang Y, Liu T, Meyer CA, Eeckhoute J, Johnson DS, Bernstein BE, Model-based analysis of ChIP-Seq (MACS). Genome Biol 2008;9(9). 10.1186/gb-2008-9-9-r137 PMC2592715 18798982

[51] Amemiya HM, Kundaje A, Boyle AP. The ENCODE blacklist: identification of problematic regions of the genome. Sci Rep 2019;9. 10.1038/s41598-019-45839-z PMC6597582 31249361

[52] Quinlan AR, Hall IM. BEDTools: a flexible suite of utilities for comparing genomic features. Bioinformatics 2010;26(6):841–2. 20110278 10.1093/bioinformatics/btq033 PMC2832824

[53] Nair NU, Das Sahu A, Bucher P, Moret BME. Chipnorm: a statistical method for normalizing and identifying differential regions in histone modification ChIP-seq libraries. PLoS One 2012;7(8). 10.1371/journal.pone.0039573 PMC3411705 22870189

[54] Waltman L, van Eck NJ. A smart local moving algorithm for large-scale modularity-based community detection. Eur Phys J B 2013;86(11).

[55] Newman MEJ, Girvan M. Finding and evaluating community structure in networks. Phys Rev E 2004;69(2). 10.1103/PhysRevE.69.026113 14995526

[56] Blondel VD, Guillaume JL, Lambiotte R, Lefebvre E. Fast unfolding of communities in large networks. Journal of Statistical Mechanics-Theory and Experiment 2008;10:P10008.

[57] Boorsma A, Foat BC, Vis D, Klis F, Bussemaker HJ. T-profiler: scoring the activity of predefined groups of genes using gene expression data. Nucleic Acids Res 2005;33(Web Server issue):W592–5. 15980543 10.1093/nar/gki484 PMC1160244

[58] Farooq A, Troen G, Delabie J, Wang J. Integrating whole genome sequencing, methylation, gene expression, topological associated domain information in regulatory mutation prediction: a study of follicular lymphoma. Comput Struct Biotechnol J 2022;20:1726–42. 35495111 10.1016/j.csbj.2022.03.023 PMC9024376

[59] Wang J Computational study of associations between histone modification and protein-DNA binding in yeast genome by integrating diverse information. BMC Genomics 2011;12:172. 21457549 10.1186/1471-2164-12-172 PMC3082246

[60] van den Heuvel MP, Sporns O. Network hubs in the human brain. Trends Cogn Sci 2013;17(12):683–96. 24231140 10.1016/j.tics.2013.09.012

[61] Sherman BT, Hao M, Qiu J, Jiao X, Baseler MW, Lane HC, DAVID: a web server for functional enrichment analysis and functional annotation of gene lists (2021 update). Nucleic Acids Res 2022;50(W1):W216–21. 35325185 10.1093/nar/gkac194 PMC9252805

[62] Liu N, Low WY, Alinejad-Rokny H, Pederson S, Sadlon T, Barry S, Seeing the forest through the trees: prioritising potentially functional interactions from Hi-C. Epigenetics Chromatin 2021;14(1). 10.1186/s13072-021-00417-4 PMC8399707 34454581

[63] Wang XW, Qiao D, Cho MH, DeMeo DL, Silverman EK, Liu YY. A statistical physics approach for disease module detection. Genome Res 2022;32(10):1918–29. 36220609 10.1101/gr.276690.122 PMC9712625

[64] Fang K, Wang JB, Liu L, Jin VX. Mapping nucleosome and chromatin architectures: a survey of computational methods. Comput Struct Biotec 2022;20:3955–62. 10.1016/j.csbj.2022.07.037 PMC9340519 35950186

[65] Huang C, Su L, Chen Y, Wu S, Sun R, Xu Q, Ceramide kinase confers tamoxifen resistance in estrogen receptor-positive breast cancer by altering sphingolipid metabolism. Pharmacol Res 2023;187:106558. 36410675 10.1016/j.phrs.2022.106558

[66] Subramani T, Yeap SK, Ho WY, Ho CL, Omar AR, Aziz SA, Vitamin C suppresses cell death in MCF-7 human breast cancer cells induced by tamoxifen. J Cell Mol Med 2014;18(2):305–13. 24266867 10.1111/jcmm.12188 PMC3930417

[67] Shannon P, Markiel A, Ozier O, Baliga NS, Wang JT, Ramage D, Cytoscape: a software environment for integrated models of biomolecular interaction networks. Genome Res 2003;13(11):2498–504. 14597658 10.1101/gr.1239303 PMC403769

[68] Fu YJ, Wang Z, Luo CX, Wang Y, Wang YP, Zhong XR, Downregulation of CXXC finger protein 4 leads to a tamoxifen-resistant phenotype in breast cancer cells through activation of the Wnt/β-catenin pathway. Transl Oncol 2020;13(2):423–40. 31911277 10.1016/j.tranon.2019.12.005 PMC6948370

[69] Wu TT, Yang WN, Sun AQ, Wei ZX, Lin Q. The role of CXC chemokines in cancer progression. Cancers 2023;15(1). 10.3390/cancers15010167 PMC9818145 36612163

[70] Han ZJ, Li YB, Yang LX, Cheng HJ, Liu X, Chen H. Roles of the CXCL8-CXCR1/2 axis in the tumor microenvironment and immunotherapy. Molecules 2021;27(1):137. 35011369 10.3390/molecules27010137 PMC8746913

[71] Reyes N, Figueroa S, Tiwari R, Geliebter J. CXCL3 signaling in the tumor microenvironment. Adv Exp Med Biol 2021;1302:15–24. 34286438 10.1007/978-3-030-62658-7_2

[72] Smilde AK, Kiers HA, Bijlsma S, Rubingh CM, van Erk MJ. Matrix correlations for high-dimensional data: the modified RV-coefficient. Bioinformatics 2009;25(3):401–5. 19073588 10.1093/bioinformatics/btn634

[73] Shin H, Shi Y, Dai C, Tjong H, Gong K, Alber F, TopDom: an efficient and deterministic method for identifying topological domains in genomes. Nucleic Acids Res 2016;44(7):e70. 26704975 10.1093/nar/gkv1505 PMC4838359

[74] Li W, Gong K, Li Q, Alber F, Zhou XJ. Hi-corrector: a fast, scalable and memory-efficient package for normalizing large-scale Hi-C data. Bioinformatics 2015;31(6):960–2. 25391400 10.1093/bioinformatics/btu747 PMC4380031

[75] Kruse K, Hug CB, Vaquerizas JM. FAN-C: a feature-rich framework for the analysis and visualisation of chromosome conformation capture data. Genome Biol 2020;21(1):303. 33334380 10.1186/s13059-020-02215-9 PMC7745377

[76] Wu C, Huang J. Enhancer selectivity across cell types delineates three functionally distinct enhancer-promoter regulation patterns. BMC Genomics 2024;25(1):483. 38750461 10.1186/s12864-024-10408-w PMC11097474

[77] Park PJ. ChIP-seq: advantages and challenges of a maturing technology. Nat Rev Genet 2009;10(10):669–80. 19736561 10.1038/nrg2641 PMC3191340

[78] Marguerat S, Bahler J. RNA-seq: from technology to biology. Cell Mol Life Sci 2010;67(4):569–79. 19859660 10.1007/s00018-009-0180-6 PMC2809939

[79] Zhang Y, An L, Xu J, Zhang B, Zheng WJ, Hu M, Enhancing Hi-C data resolution with deep convolutional neural network HiCPlus. Nat Commun 2018;9 (1):750. 29467363 10.1038/s41467-018-03113-2 PMC5821732

[80] Girvan M, Newman MEJ. Community structure in social and biological networks. Proc Natl Acad Sci USA 2002;99(12):7821–6. 12060727 10.1073/pnas.122653799 PMC122977

[81] Barski A, Cuddapah S, Cui KR, Roh TY, Schones DE, Wang ZB, High-resolution profiling of histone methylations in the human genome. Cell 2007;129 (4):823–37. 17512414 10.1016/j.cell.2007.05.009

[82] Imakaev M, Fudenberg G, McCord RP, Naumova N, Goloborodko A, Lajoie BR, Iterative correction of Hi-C data reveals hallmarks of chromosome organization. Nat Methods 2012;9(10):999–1003. 22941365 10.1038/nmeth.2148 PMC3816492

[83] Wolff J, Rabbani L, Gilsbach R, Richard G, Manke T, Backofen R, Galaxy HiCExplorer 3: a web server for reproducible Hi-C, capture Hi-C and single-cell Hi-C data analysis, quality control and visualization. Nucleic Acids Res 2020;48(W1):W177–84. 32301980 10.1093/nar/gkaa220 PMC7319437

[84] Yardimci GG, Ozadam H, Sauria MEG, Ursu O, Yan KK, Yang T, Measuring the reproducibility and quality of Hi-C data. Genome Biol 2019;20(1):57. 30890172 10.1186/s13059-019-1658-7 PMC6423771

[85] DeSantis CE, Ma JM, Gaudet MM, Newman LA, Miller KD, Sauer AG, Breast cancer statistics, 2019. Ca-Cancer J Clin 2019;69(6):438–51. 31577379 10.3322/caac.21583

[86] Wang X, Wang S. Identification of key genes involved in tamoxifen-resistant breast cancer using bioinformatics analysis. Transl Cancer Res 2021;10(12):5246–57. 35116374 10.21037/tcr-21-1276 PMC8798269

[87] Hartkopf AD, Grischke EM, Brucker SY. Endocrine-resistant breast cancer: mechanisms and treatment. Breast Care (Basel) 2020;15(4):347–54. 32982644 10.1159/000508675 PMC7490658

[88] Guerrero-Zotano A, Mayer IA, Arteaga CL. PI3K/AKT/mTOR: role in breast cancer progression, drug resistance, and treatment. Cancer Metastasis Rev 2016;35(4):515–24. 27896521 10.1007/s10555-016-9637-x

[89] Mishra A, Srivastava A, Pateriya A, Tomar MS, Mishra AK, Shrivastava A. Metabolic reprograming confers tamoxifen resistance in breast cancer. Chem Biol Interact 2021;347:109602. 34331906 10.1016/j.cbi.2021.109602

[90] Steifensand F, Gallwas J, Bauerschmitz G, Gründker C. Inhibition of metabolism as a therapeutic option for tamoxifen-resistant breast cancer cells. Cells 2021;10(9). 10.3390/cells10092398 PMC8467413 34572047

