# Supplementary material for: Integrated analysis of differential intra-chromosomal community interactions: A study of breast cancer
Source: Artif Intell Med. Author manuscript; Available in PMC 2026 Jul 16. (PMC13373883; doi:10.1016/j.artmed.2025.103180)
Supplement: 1 [file NIHMS2176752-supplement-1.docx]

**Supplementary for “Integrated Analysis of Differential Intra-chromosomal Community Interactions: A Study of Breast Cancer”**

**Supplementary Methods**

**Building adjacency matrices with predefined resolution for Hi-C interactions**

If a chromosome length is *L* (e.g., 22 human chromosomes and an X chromosome), then the number of window bins $n_{bin}$ of this chromosome based on a predefined window bin size or interaction resolution (e.g., bin=500kb) is

|  | $n_{bin}=\left\lceil\frac{L}{bin} \right\rceil,$ | (1) |
| --- | --- | --- |

where $\left\lceil x \right\rceil$ is a ceiling function, returning the smallest integer greater than or equal to $x$. Then, the *i*-th interval (or window bin) of a chromosome is

|  | $w_{i}: \left[ \left( i-1 \right)\cdot bin, \min\left( i\cdot bin,L \right) \right), i\in\left\{ 1,2,\cdots,n_{bin} \right\}.$ | (2) |
| --- | --- | --- |

For a set of ${H(h_{1},\cdots,h_{K})}^{T}$ with *K* Hi-C intra-chromosomal interactions (e.g., significant interactions identified by HOMER), each pair of interactions$h_{k}\left( x_{k},y_{k} \right)$will be mapped to a pair of window bins

|  | $h_{k}\left( x_{k},y_{k} \right)\to w_{ij}\left( w_{i},w_{j} \right),$ | (3) |
| --- | --- | --- |

where $x_{k}$ and $y_{k}$ are the positions of a pair of Hi-C interactions in intervals (or window bins) $w_{i}$ and $w_{j}$, respectively, and $w_{ij}$ is an interaction between window $w_{i}$ and $w_{j}$. Thus, some $w_{ij}$ might contain several pairs of Hi-C interactions while others might have none. Subsequently, an adjacency matrix *A* for intra-chromosomal interactions can be obtained

|  | $A_{ij}=I_{\left\{ w_{ij} \cup w_{ji}\neq\emptyset\right\}}, i,j\in\left\{ 1,2,\cdots,n_{bin} \right\},$ | (4) |
| --- | --- | --- |

where $A_{ij}=1$ if the union ($\cup$) of $w_{ij}$ and $w_{ji}$ is not empty ($\emptyset$), otherwise $A_{ij}=0$. In this way, intra-chromosomal Hi-C interactions are transformed to a binary adjacency matrix *A* with $A_{ij}=A_{ji}$. Here, if different window bin sizes (e.g., bin size = 500kb, 100kb, or 50kb) are used to map Hi-C interactions, then intra-chromosomal interaction networks with different resolutions will be obtained.

**Generating genomic feature matrices based on multi-omics data for Hi-C interactions**

First, Z-scores of intra-chromosomal interactions obtained from Hi-C data are mapped to the aforementioned adjacency matrix (e.g., bin size=500kb), as the edge weights of intra-chromosomal interactions (e.g., 22 human chromosomes and an X chromosome) for untreated, one hours E2-treated MCF7 cells, and MCF7TR cells, respectively. Then, genomic features (e.g., nucleosome density, histone modifications, and gene expressions) are mapped to the same adjacency matrix for generating genomic feature matrices, respectively. For example, ${H^{(g)}(h_{1}^{(g)},\cdots,h_{M}^{(g)})}^{T}$ is genomic feature *g* with *M* data points, where each $h_{m}^{(g)}(x_{m},z_{m})$ contains position information $x_{m}$ and Z-score $z_{m}$ of feature *g* in the loci. For a set of genomic features $W_{i}$ in a window bin $w_{i}$

|  | $W_{i}=\left\{ \left. z_{m} \right\vert x_{m}\cap w_{i}\neq\emptyset,m\in\left\{ 1,2,\cdots,M \right\} \right\},$ | (5) |
| --- | --- | --- |

if loci $x_{m}$ intersects with bin $w_{i}$ (e.g., $x_{m}\cap w_{i}\neq\emptyset$), then feature $z_{m}$ will be included in this bin. Otherwise, the feature $z_{m}$ will not be considered because loci $x_{m}$ is not in bin $w_{i}$ (e.g., $x_{m}\cap w_{i}=\emptyset$). Since there may be multiple (or none) $z_{m}$ in the same bin $w_{i}$, a genomic feature Z-score for the bin $w_{i}$ is

|  | $Z_{i}=\left\{ \begin{aligned} \bar{W_{i}}, W_{i}\neq\emptyset\\ 0, W_{i}=\emptyset\end{aligned}, \right.$ | (6) |
| --- | --- | --- |

where $\bar{W_{i}}$ is the mean of these $z_{m}$. Subsequently, a feature matrix $F^{(g)}$ can be built for each genomic feature *g* based on the adjacency matrix *A* of Hi-C intra-chromosomal interactions. For instance, if there is an interaction between the window bins $w_{i}$ and $w_{j}$ (e.g., $A_{ij}=1$), then an edge weight $F_{ij}^{(g)}$ of the interaction $w_{ij}$ at feature matrix *F* is assigned by equation

|  | $F_{ij}^{(g)}=\max\left( 0,Z_{i}+Z_{j} \right).$ | (7) |
| --- | --- | --- |

It indicates the edge weight of feature matrix $F^{(g)}$ is determined by the Z-scores of a pair of interacting bins (e.g., $w_{i}$ and $w_{j}$). Such edge weight assignment (e.g., a weighted edge approach) for a network graph is adopted from a previous work [1], which is used here to build genomic feature matrices $F^{(g)}$ for adjacency matrix *A*. In order words, if there is an interaction in *A*, then the available genomic features will be considered correspondingly for building genomic features matrices $F^{(g)}$. After converting multi-omics datasets $H^{(g)}$ to genomic feature matrices $F^{(g)}$, based on the intra-chromosomal interactions *A* derived from Hi-C experiments (e.g., in untreated, one hours E2-treated MCF7 cells, and MCF7TR cells, respectively), information from diverse sources is comparable and integrable through the intra-chromosomal interactions.

**A comparison of genomic feature enrichments between the mean and the median mapping of Z-scores to Hi-C interaction matrix**

There are two types of approaches to compute genomic features (e.g., nucleosome density, histone modifications, and gene expressions) in a Hi-C adjacency matrix: one is the mean of Z-scores in a window bin (e.g., value $\bar{W_{i}}$ ) which represent the average signal of a genomic feature in window bin $w_{i}$ as shown in the Equation (6), and the other is the median of Z-scores such as shown in the following equation

|  | $Z_{i}=\left\{ \begin{aligned} med(W_{i}), W_{i}\neq\emptyset\\ 0, W_{i}=\emptyset\end{aligned}, \right.$ | (8) |
| --- | --- | --- |

where $med(W_{i})$ represents the median value of genomic feature set $W_{i}$ in a window bin. Based on these mapped genomic features in the Hi-C interaction matrix (e.g., an adjacency matrix of intra-chromosomal interactions), the same genomic feature enrichment test as described in the main text can be applied to obtain heatmaps/matrices of various genomic feature enrichments. For example, if a genomic feature enrichment heatmap or matrix derived from the mean calculation (Equation (6)) is *A* and a corresponding matrix obtained from median function (Equation (8)) is *B*, then a similarity between the *A* and the *B* is measured by a multivariate index (RV-coefficient)[2, 3] as shown in below function

|  | $RV(A,B)=\frac{tr(AA^{T}BB^{T})}{\sqrt{tr[{(AA^{T})}^{2}]tr[{(BB^{T})}^{2}]}},$ | (9) |
| --- | --- | --- |

where $tr$ denotes the trace of a square matrix. Here, the larger the $RV(A,B)$ the more similar the two matrices. Thus, RV-coefficient can be used to evaluate the similarity between the two enriched genomic feature matrices (e.g., $RV\in[0,1]$). In addition, differential interacting and expressed genes (DIEGs) obtained from differential network analysis (e.g., untreated MCF7 cell vs. tamoxifen-resistant MCF7TR cell) based on either mean or median mapping of Z-scores to Hi-C adjacency matrix are also compared to access the robust of proposed network analysis.

**Supplementary Results**

**Finding parameters for HOMER at different resolutions**

So far, the proposed analysis pipeline was only evaluated at a single resolution (e.g., window bin size = 500kb) with a fixed number of edges for valid communities (e.g., $e^{*}>20$). The results indicate that the quality of inferred community structures is better by using significant chromosomal interactions identified by HOMER than that by an a*d hoc* filtering of the weakest interactions (Figures 2 and 3). For studying chromosomal interactions in different resolutions (e.g., window bin size = 50kb, 100kb, and 500kb), new methods are needed to fine-tunning HOMER parameters and to estimate a minimum size of valid communities. Firstly, there are two essential parameters (the window bin resolution and super resolution) in HOMER for identifying significant chromosomal interactions. In order to evaluate the impact of these parameters on HOMER, they are initially set to equal as recommended by the HOMER manual, then they are changed dynamically. For instance, in SFigure 12A, the mean number of significant interactions found by HOMER (e.g., untreated and one hours E2 treated MCF7 cells) reaches a peak when the window bin size =50kb (e.g., the dashed lines in SFigure 12A), but it drops significantly after the bin size =10kb. At the same time, the median distance of identified significant Hi-C interactions drops sharply after the bin size reaches 100kb (e.g., the box plot in SFigure 12A), which indicates many long distance Hi-C interactions were excluded at here. These plots motivated us to find a way to estimate the optimal window bin size (or resolution) and the super resolution in HOMER: first, to fix the window resolution at 50kb but increase the super resolution from 50kb to 500kb; then, the higher the super resolution the larger the median distance for significant interactions (e.g., the box plot in SFigure 12B), while the number of significant interactions is gradually decreasing such as a significant drop at the super resolution 200kb (e.g., the dash line plot in SFigure 12B). To have a tradeoff between the median distance of interactions and the number of interactions, the optimal window bin resolution and super resolution for HOMER at a specific interaction resolution can be estimated (e.g., window bin size = 50k and super resolution = 200kb; SFigure 12B). In other words, optimal HOMER parameters allow for the identification of as many significant interactions as possible, while remaining a reasonable median interaction distance. Based on SFigure 12, if the window bin size is 50kb, 100kb, and 500kb, then the HOMER super resolution is 200kb, 200kb, and 500kb, respectively. Thus, this strategy can be used by HOMER to identify significant chromosomal interactions, while maintaining similar chromosomal interaction structures in different interaction resolutions.

**Optimizing the parameters for valid communities at different resolutions**

The second essential parameter, for studying intra-chromosomal interactions at different resolutions (or window bin size), is the size of valid communities - the minimum size (or number of interactions) of valid communities. As described in the Method section, $p_{rj}$ is the normalized number of edges in a community (or network cluster), which has similar distributions and scales at different network resolutions (e.g., the light green bars in SFigure 13A; bin size=500kb, 100kb, or 50kb). By setting a unified percentage cutoff value (e.g., $=0.02$ ; the black dashed line in bar plots of SFigures 13A and 13B) for the minimum number of edges in a valid community (e.g., the light pink bars in SFigure 13B and the bold row in Supplementary Table S13), the majority of small communities are excluded (e.g., have very small number of edges; a peak of light pink bars at the left most X-axis in SFigure 13B). A list of expected minimum size of valid communities at different interaction resolutions is shown in Supplementary Table S13, which follow a real-world scale of window bin sizes. For example, if a window bin size is 500kb, then the minimum size of valid communities is ~20 edges (or interactions). Nevertheless, if the bin size is 100kb then the minimum size becomes ~100 edges, where both the resolution and the minimum size are increased five times. In other words, if the intra-chromosomal interaction resolution reaches 50kb, then the minimum size of valid communities is at least 200 edges. In Supplementary Table S13, different percentage cutoff values *p* (e.g., $p=0.01 or 0.05$) were used to estimate the minimum size of valid communities at different resolutions, where $p=0.02$ gives the best results for identifying the minimum size of valid communities. That is because the other thresholds remove either too few (Supplementary Figure S14; $p=0.01$) or too many (Supplementary Figure S15; $p=0.05$) invalid communities.

**Supplementary Figures**

**SFigure 1. Heatmaps of an intra-chromosomal interaction matrix at 500kb resolution for chromosome 3 and the corresponding genomic feature matrices in untreated MCF7 cells.**

**
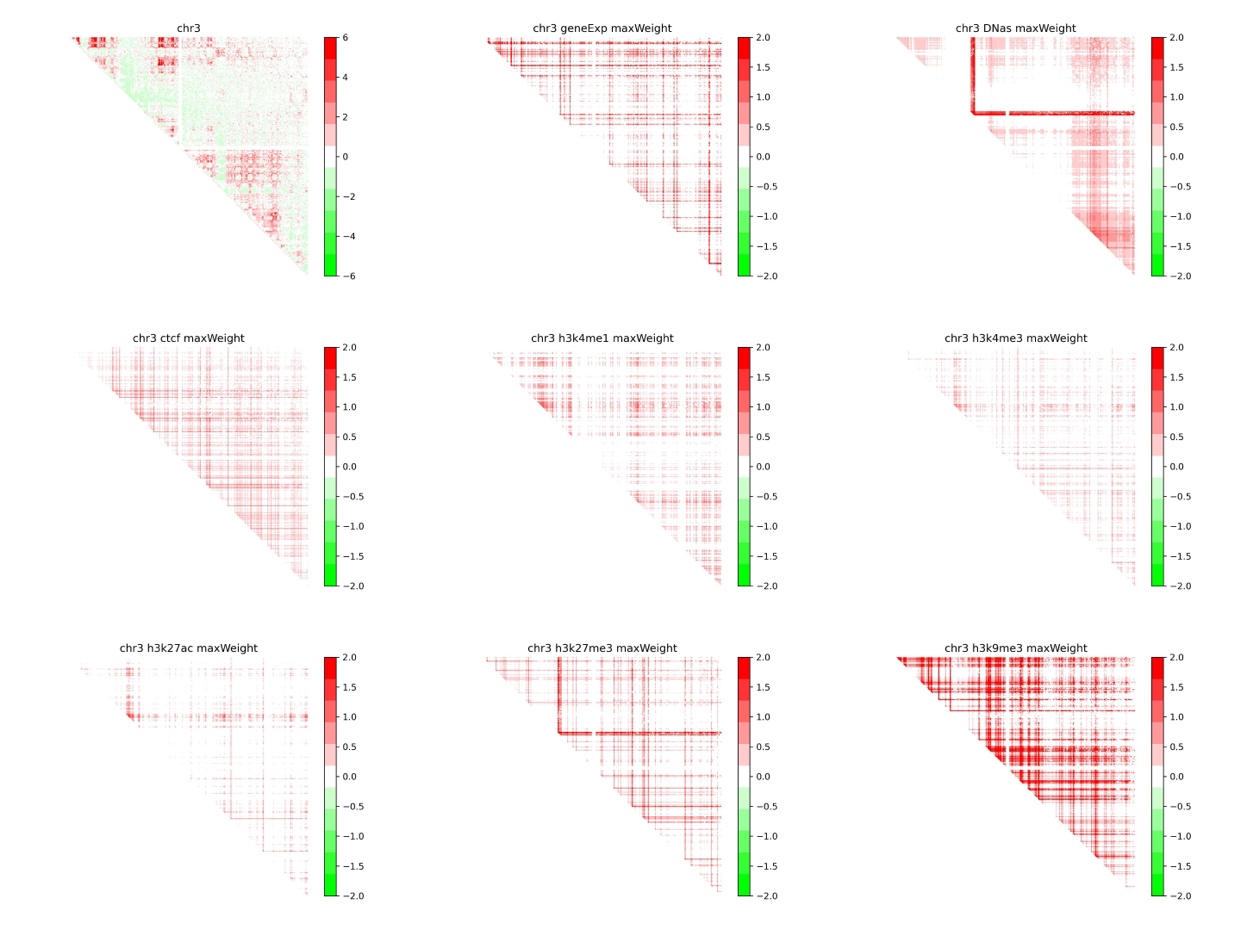
**

The first heatmap is an intra-chromosomal interaction matrix of chr3, where Z-scores are scaled to red and green color for positive and negative values, respectively. This intra-chromosomal interaction matrix will be used to predict intra-chromosomal community interactions. The rest of eight heatmaps are the corresponding genomic features in the same chromosome follows intra-chromosomal interactions. A weight of genomic feature $F_{ij}$in an edge (or an intra-chromosomal interaction) is calculated by $F_{ij}=max(0, W_{i}+W_{j})$, where *W_i_* and *W_j_* are genomic feature in the corresponding two nodes (or window bins). This calculation is similar to a previous published network study[1]. The eight genomic features are: “geneExp” and “DNas” represent gene expression profiles and nucleosome densities in an intra-chromosomal interaction, respectively; “CTCF” indicates an average binding of insulators; “H3K27ac” and “H3K4me1” are enhancer markers; “H3K4me3” is promoter marker; “H3K27me3” and “H3K9me3” represent repressors. All values in heatmaps are scaled to red and green colors for representing positive and negative values.

**SFigure 2. Heatmaps of an intra-chromosomal interaction matrix at 500kb resolution for chromosome 17 and the corresponding genomic feature matrices in untreated MCF7 cells.**

**
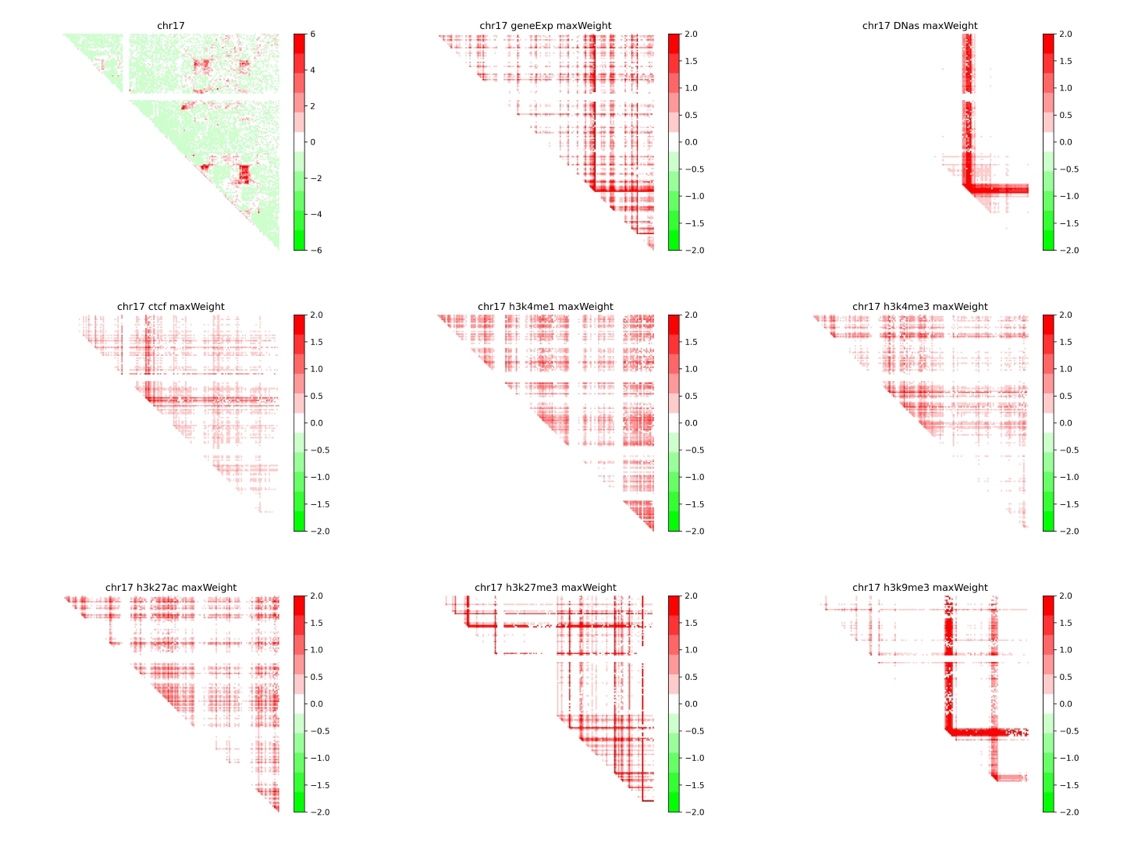
**

The first heatmap is an intra-chromosomal interaction matrix of chr17, where Z-scores are scaled to red and green color for positive and negative values, respectively. This intra-chromosomal interaction matrix will be used to predict intra-chromosomal community interactions. The rest of eight heatmaps are the corresponding genomic features in the same chromosome follows intra-chromosomal interactions. A weight of genomic feature $F_{ij}$in an edge (or an intra-chromosomal interaction) is calculated by $F_{ij}=max(0, W_{i}+W_{j})$, where *W_i_* and *W_j_* are genomic feature in the corresponding two nodes (or window bins). This calculation is similar to a previous published network study[1]. The eight genomic features are: “geneExp” and “DNas” represent gene expression profiles and nucleosome densities in an intra-chromosomal interaction, respectively; “CTCF” indicates an average binding of insulators; “H3K27ac” and “H3K4me1” are enhancer markers; “H3K4me3” is promoter marker; “H3K27me3” and “H3K9me3” represent repressors. All values in heatmaps are scaled to red and green colors for representing positive and negative values.

**SFigure 3.** **Heatmaps of an intra-chromosomal interaction matrix at 500kb resolution for chromosome 20 and the corresponding genomic feature matrices in untreated MCF7 cells.**


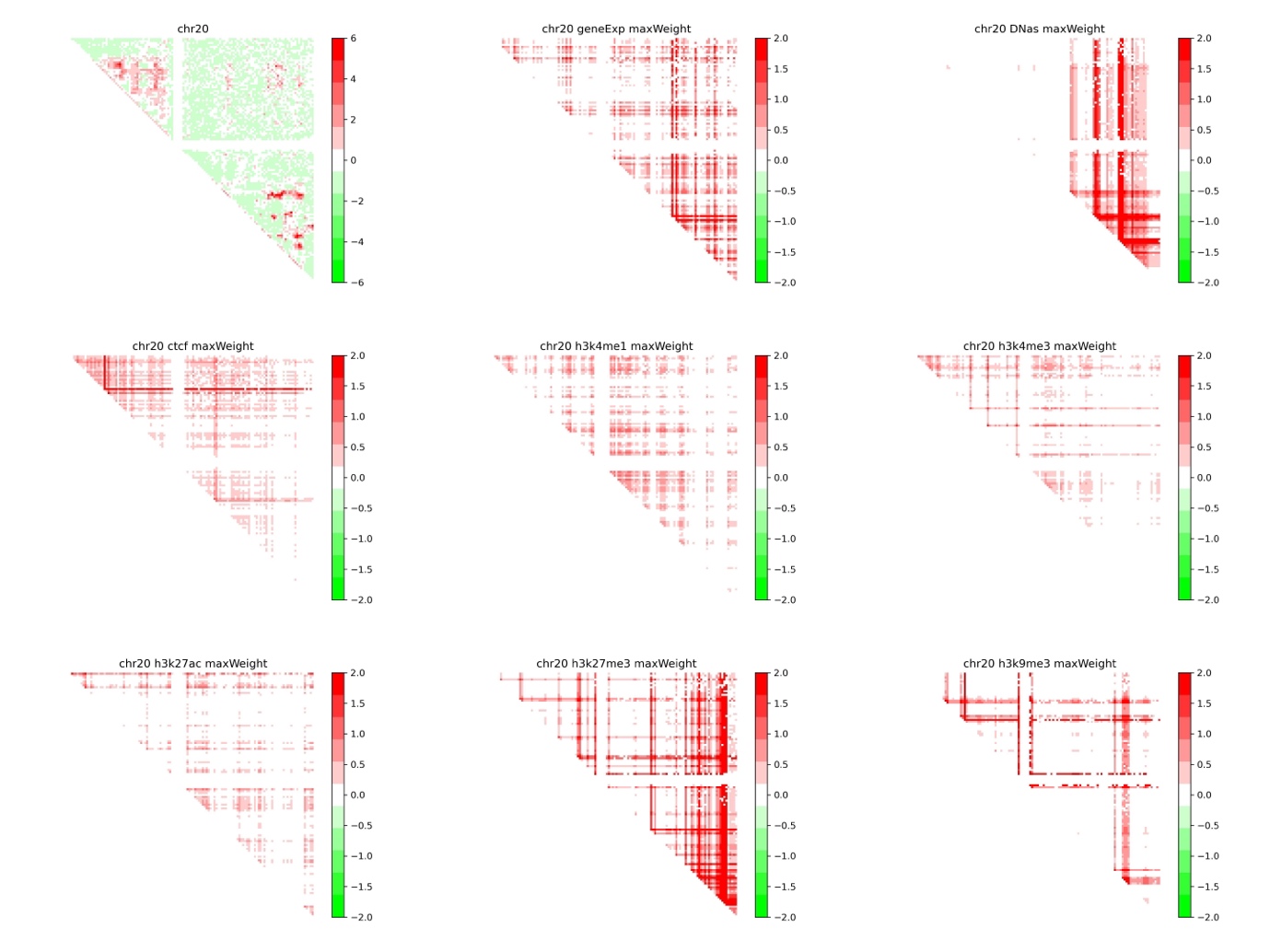


The first heatmap is an intra-chromosomal interaction matrix of chr20, where Z-scores are scaled to red and green color for positive and negative values, respectively. This intra-chromosomal interaction matrix will be used to predict intra-chromosomal community interactions. The rest of eight heatmaps are the corresponding genomic features in the same chromosome follows intra-chromosomal interactions. A weight of genomic feature $F_{ij}$in an edge (or an intra-chromosomal interaction) is calculated by $F_{ij}=max(0, W_{i}+W_{j})$, where *W_i_* and *W_j_* are genomic feature in the corresponding two nodes (or window bins). This calculation is similar to a previous published network study[1]. The eight genomic features are: “geneExp” and “DNas” represent gene expression profiles and nucleosome densities in an intra-chromosomal interaction, respectively; “CTCF” indicates an average binding of insulators; “H3K27ac” and “H3K4me1” are enhancer markers; “H3K4me3” is promoter marker; “H3K27me3” and “H3K9me3” represent repressors. All values in heatmaps are scaled to red and green colors for representing positive and negative values.

**SFigure 4. Violin plot of modularity scores and number of edges based on different selection of intra-chromosomal interactions in Hi-C data at 500kb resolution for one hours E2 treated MCF7 cells.**

**
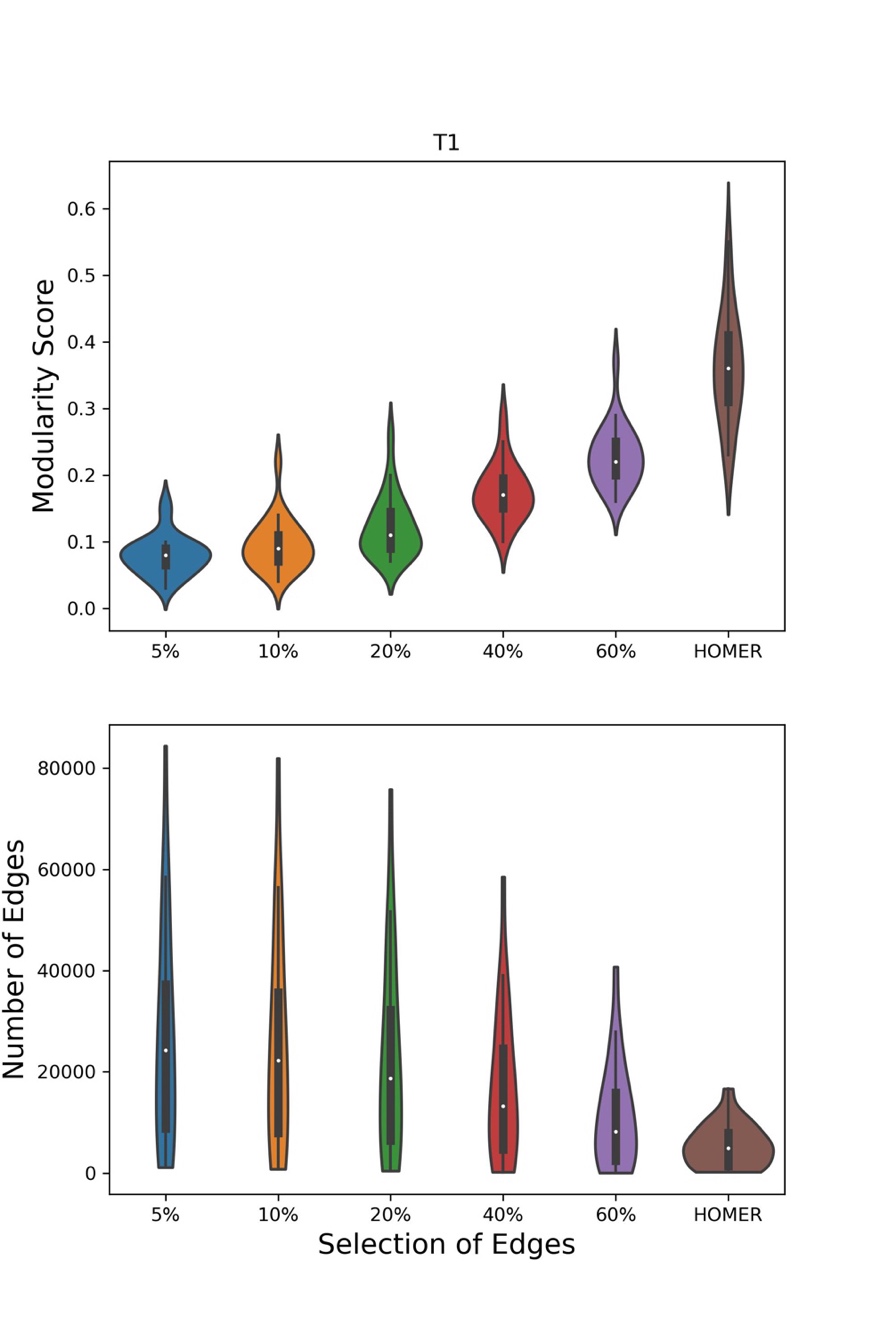
**

The upper panel of figure shows violin plots of modularity scores[4] for intra-chromosomal community interactions in 23 chromosomes that were calculated based on the filtering of 5, 10, 20, 40, 60 percentages of the weakest intra-chromosomal interactions genome-widely, respectively. “HOMER” represent the significant intra-chromosomal interactions predicted by HOMER[5] program. The lower panel of figure displays violine plots of the number of edges (or intra-chromosomal interactions) in 23 chromosomes after filtering 5, 10, 20, 40, 60 percentages of the weakest intra-chromosomal interactions genome-widely or selecting significant interactions by HOMER. A white dot in the violin plot represents the median value of the distribution.

**SFigure 5. Violin plot of number of valid clusters and number of edges in valid clusters based on different selection of intra-chromosomal interactions in Hi-C data at 500kb resolution for one hours E2 treated MCF7 cells.**

**
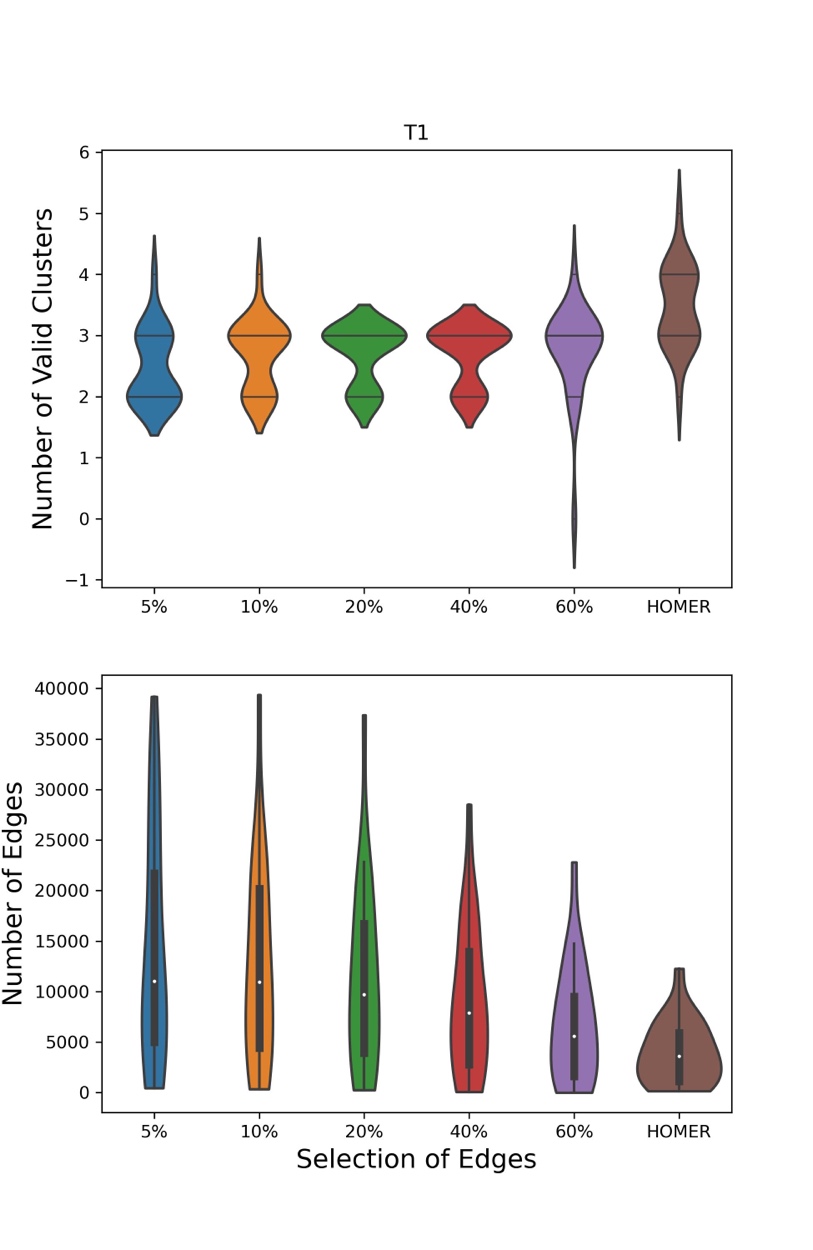
**

The upper panel of figure shows violin plots of the number of valid network clusters (e.g., communities with the number of edges > 20) in intra-chromosomal interactions among 23 chromosomes, which were predicted by filtering of 5, 10, 20, 40, 60 percentages of the weakest intra-chromosomal interactions genome-widely, respectively. “HOMER” represent the selection of significant intra-chromosomal interactions by HOMER[5] program. Here, a black line within each violin plot represents every observation inside the distribution. The lower panel of figure displays violine plots of the number of edges (or intra-chromosomal interactions) in valid clusters of intra-chromosomal interactions among 23 chromosomes after filtering of 5, 10, 20, 40, 60 percentages of the weakest intra-chromosomal interactions genome-widely or selecting significant interactions by HOMER. A white dot in the violin plot represents the median value of the distribution.

**SFigure 6.** **Heatmaps of intra-chromosomal community interactions for chromosomes 3, 17 and 20 after removing the lowest 40 percentages of intra-chromosomal interactions at 500kb resolution in untreated MCF7 cells**

**
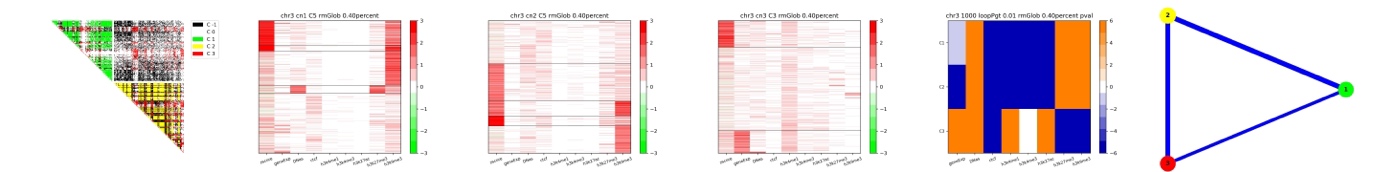
**

**
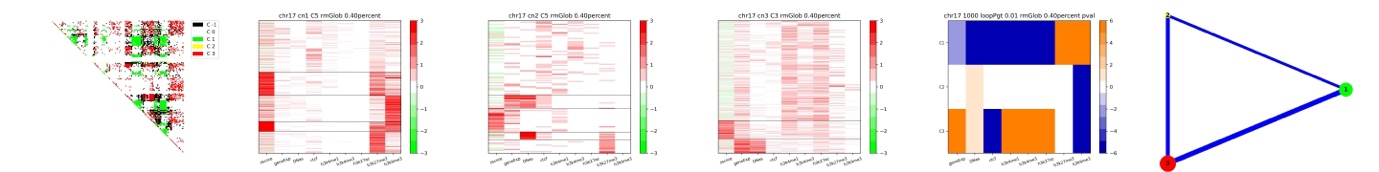
**

**
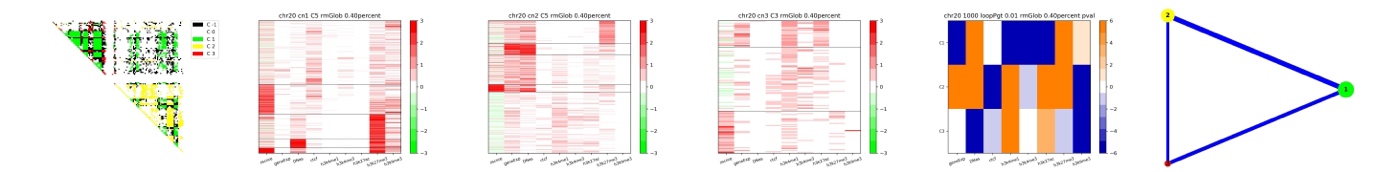
**

In each panel, *the left most heatmaps* are intra-chromosomal interaction matrices where interactions are colored by their community (or network cluster) labels. For example, black indicates two nodes of an edge (or interaction) do not belong to the same community, but the other colors represent the community label of an interaction. *The right most figures* are network plots of predicted intra-chromosomal community interactions (only show communities with more than 20 edges) in a chromosome, where the size of nodes and the width of edges indicates the number of edges (interactions) in the community and the number communications between the communities, respectively. The color of nodes indicates the community label. In a panel, *the second right most figures* are heatmaps of log10 transformed P-values for enrichment of a genomic feature in a community (or network cluster) against that from randomly generated (e.g., the number of P-value < 0.01 in 1000 times random permutation T-tests) intra-chromosomal interactions, where the orange and blue colors represent positive and negative enrichment of the feature, respectively. This sign of the enrichment is inferred from the expected T-values of random permutation T-test. The rest of red and green coded heatmaps are genomic features in intra-chromosomal community interactions: “Zscore” is the Z-score of intra-chromosomal interactions; “geneExp”, “DNas” and “CTCF” are average of gene expressions, nucleosome densities, and insulator in the interactions, respectively; “H3K4me1” and “H3K27ac” are enhancer markers; “H3K4me3” is a promoter maker; “H3K27me3” and “H3K9me3” are repressor markers. Here, “cn1”, “cn2”, and “cn3” are community number 1, 2, and 3 in a chromosome, respectively, which are the same numbers as the node labels in the network plots.

**SFigure 7.** **Heatmaps of intra-chromosomal community interactions for chromosomes 3, 17 and 20 after removing the lowest 40 percentages of intra-chromosomal interactions at 500kb resolution in one hours E2 treated MCF7 cells**

**
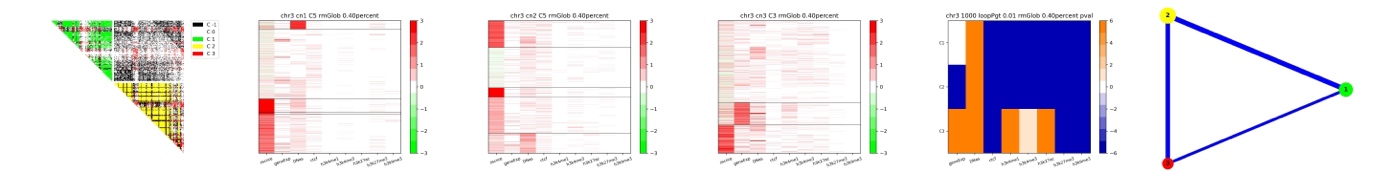
**

**
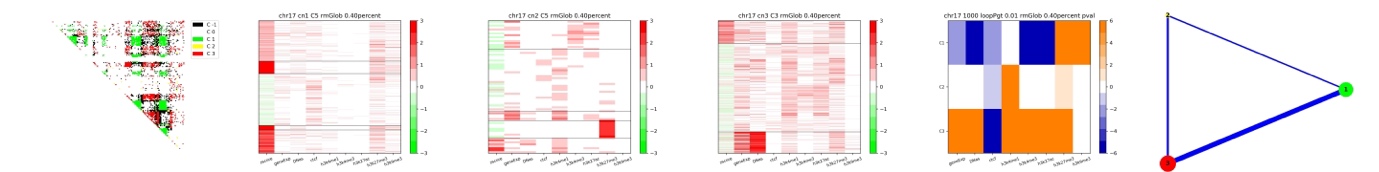
**

**
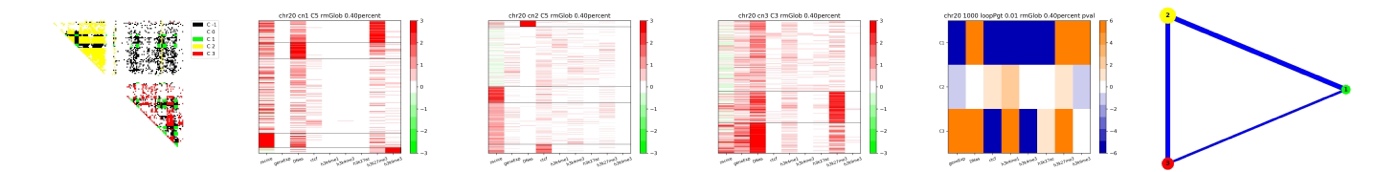
**

In each panel, *the left most heatmaps* are intra-chromosomal interaction matrices where interactions are colored by their community (or network cluster) labels. For example, black indicates two nodes of an edge (or interaction) do not belong to the same community, but the other colors represent the community label of an interaction. *The right most figures* are network plots of predicted intra-chromosomal community interactions (only show communities with more than 20 edges) in a chromosome, where the size of nodes and the width of edges indicates the number of edges (interactions) in the community and the number communications between the communities, respectively. The color of nodes indicates the community label. In a panel, *the second right most figures* are heatmaps of log10 transformed P-values for enrichment of a genomic feature in a community (or network cluster) against that from randomly generated (e.g., the number of P-value < 0.01 in 1000 times random permutation T-tests) intra-chromosomal interactions, where the orange and blue colors represent positive and negative enrichment of the feature, respectively. This sign of the enrichment is inferred from the expected T-values of random permutation T-test. The rest of red and green coded heatmaps are genomic features in intra-chromosomal community interactions: “Zscore” is the Z-score of intra-chromosomal interactions; “geneExp”, “DNas” and “CTCF” are average of gene expressions, nucleosome densities, and insulator in the interactions, respectively; “H3K4me1” and “H3K27ac” are enhancer markers; “H3K4me3” is a promoter maker; “H3K27me3” and “H3K9me3” are repressor markers. Here, “cn1”, “cn2”, and “cn3” are community number 1, 2, and 3 in a chromosome, respectively, which are the same numbers as the node labels in the network plots.

**SFigure 8.** **Heatmaps of intra-chromosomal community interactions for chromosomes 3, 17 and 20 after removing the lowest 60 percentages of intra-chromosomal interactions at 500kb resolution in untreated MCF7 cells.**

**
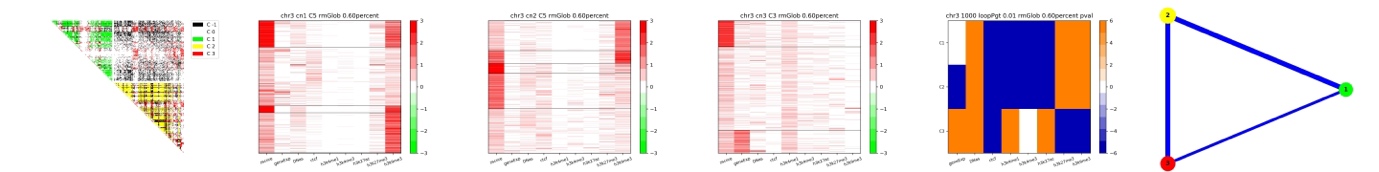
**

**
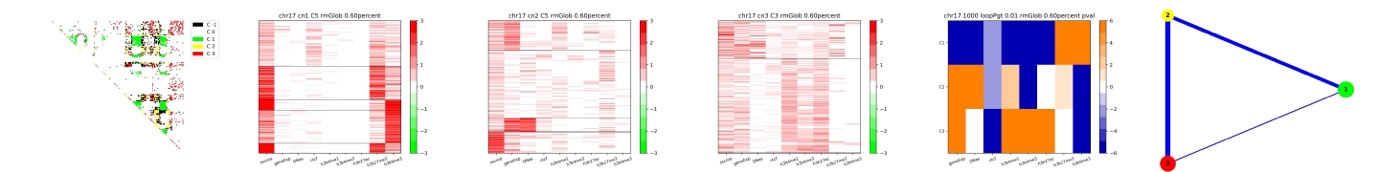
**

**
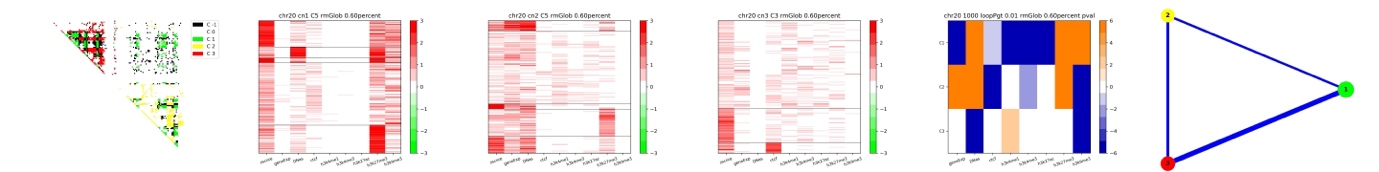
**

In each panel, *the left most heatmaps* are intra-chromosomal interaction matrices where interactions are colored by their community (or network cluster) labels. For example, black indicates two nodes of an edge (or interaction) do not belong to the same community, but the other colors represent the community label of an interaction. *The right most figures* are network plots of predicted intra-chromosomal community interactions (only show communities with more than 20 edges) in a chromosome, where the size of nodes and the width of edges indicates the number of edges (interactions) in the community and the number communications between the communities, respectively. The color of nodes indicates the community label. In a panel, *the second right most figures* are heatmaps of log10 transformed P-values for enrichment of a genomic feature in a community (or network cluster) against that from randomly generated (e.g., the number of P-value < 0.01 in 1000 times random permutation T-tests) intra-chromosomal interactions, where the orange and blue colors represent positive and negative enrichment of the feature, respectively. This sign of the enrichment is inferred from the expected T-values of random permutation T-test. The rest of red and green coded heatmaps are genomic features in intra-chromosomal community interactions: “Zscore” is the Z-score of intra-chromosomal interactions; “geneExp”, “DNas” and “CTCF” are average of gene expressions, nucleosome densities, and insulator in the interactions, respectively; “H3K4me1” and “H3K27ac” are enhancer markers; “H3K4me3” is a promoter maker; “H3K27me3” and “H3K9me3” are repressor markers. Here, “cn1”, “cn2”, and “cn3” are community number 1, 2, and 3 in a chromosome, respectively, which are the same numbers as the node labels in the network plots.

**SFigure 9.** **Heatmaps of intra-chromosomal community interactions for chromosomes 3, 17 and 20 after removing the lowest 60 percentages of intra-chromosomal interactions at 500kb resolution in one hours E2 treated MCF7 cells.**

**
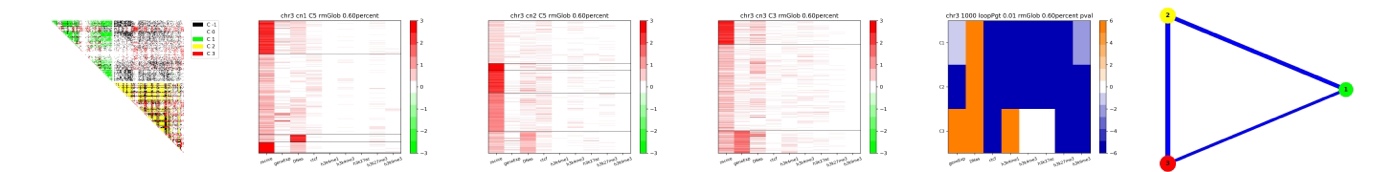
**

**
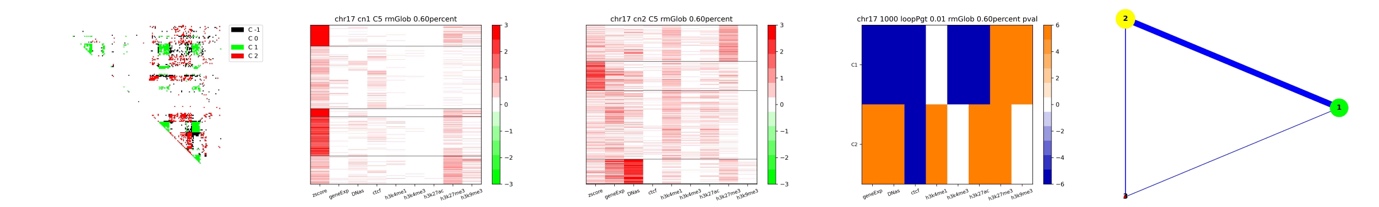
**

**
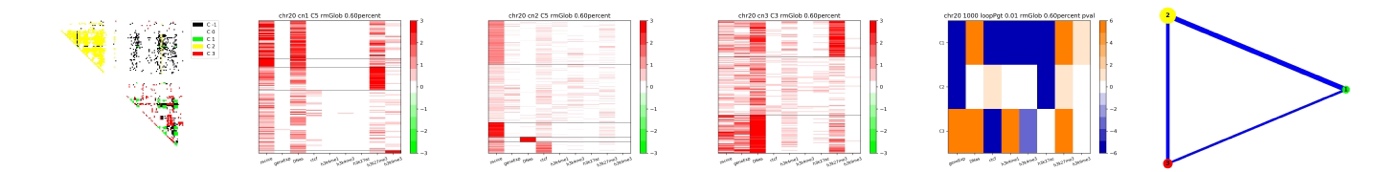
**

In each panel, *the left most heatmaps* are intra-chromosomal interaction matrices where interactions are colored by their community (or network cluster) labels. For example, black indicates two nodes of an edge (or interaction) do not belong to the same community, but the other colors represent the community label of an interaction. *The right most figures* are network plots of predicted intra-chromosomal community interactions (only show communities with more than 20 edges) in a chromosome, where the size of nodes and the width of edges indicates the number of edges (interactions) in the community and the number communications between the communities, respectively. The color of nodes indicates the community label. In a panel, *the second right most figures* are heatmaps of log10 transformed P-values for enrichment of a genomic feature in a community (or network cluster) against that from randomly generated (e.g., the number of P-value < 0.01 in 1000 times random permutation T-tests) intra-chromosomal interactions, where the orange and blue colors represent positive and negative enrichment of the feature, respectively. This sign of the enrichment is inferred from the expected T-values of random permutation T-test. The rest of red and green coded heatmaps are genomic features in intra-chromosomal community interactions: “Zscore” is the Z-score of intra-chromosomal interactions; “geneExp”, “DNas” and “CTCF” are average of gene expressions, nucleosome densities, and insulator in the interactions, respectively; “H3K4me1” and “H3K27ac” are enhancer markers; “H3K4me3” is a promoter maker; “H3K27me3” and “H3K9me3” are repressor markers. Here, “cn1”, “cn2”, and “cn3” are community number 1, 2, and 3 in a chromosome, respectively, which are the same numbers as the node labels in the network plots.

**SFigure 10.** **Heatmaps of intra-chromosomal community interactions for chromosomes 3, 17 and 20 after selecting significant intra-chromosomal interactions at 500kb resolution by HOMER in untreated MCF7 cells.**

**
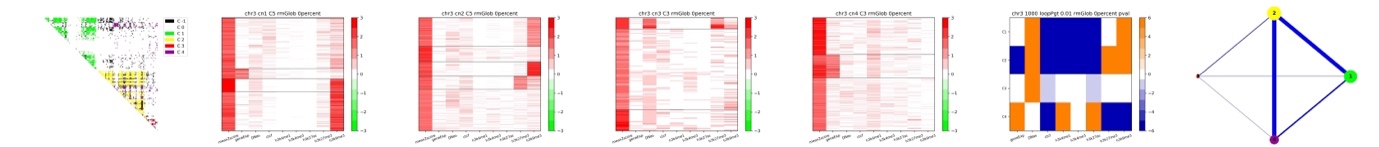
**

**
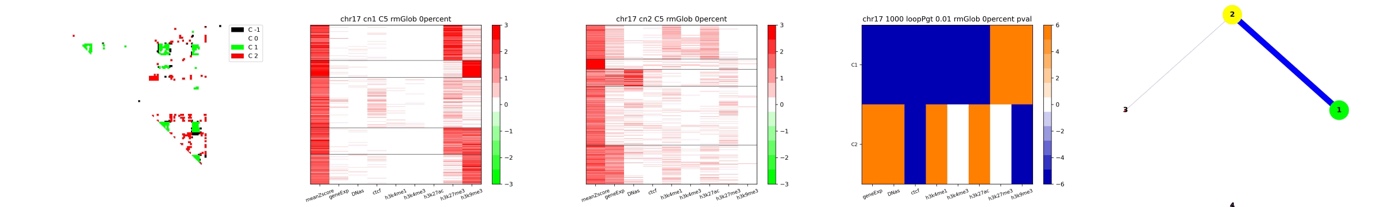
**

**
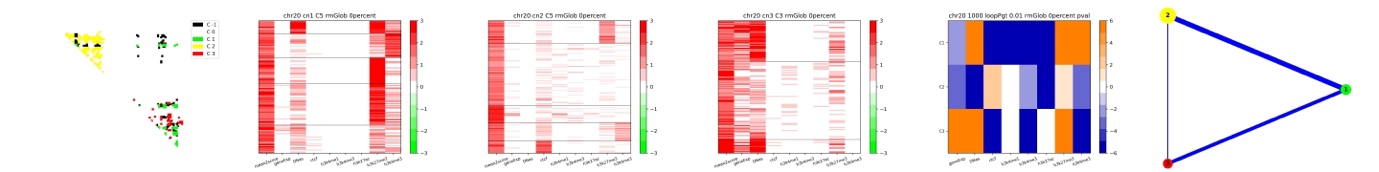
**

In each panel, *the left most heatmaps* are intra-chromosomal interaction matrices where interactions are colored by their community (or network cluster) labels. For example, black indicates two nodes of an edge (or interaction) do not belong to the same community, but the other colors represent the community label of an interaction. *The right most figures* are network plots of predicted intra-chromosomal community interactions (only show communities with more than 20 edges) in a chromosome, where the size of nodes and the width of edges indicates the number of edges (interactions) in the community and the number communications between the communities, respectively. The color of nodes indicates the community label. In a panel, *the second right most figures* are heatmaps of log10 transformed P-values for enrichment of a genomic feature in a community (or network cluster) against that from randomly generated (e.g., the number of P-value < 0.01 in 1000 times random permutation T-tests) intra-chromosomal interactions, where the orange and blue colors represent positive and negative enrichment of the feature, respectively. This sign of the enrichment is inferred from the expected T-values of random permutation T-test. The rest of red and green coded heatmaps are genomic features in intra-chromosomal community interactions: “Zscore” is the Z-score of intra-chromosomal interactions; “geneExp”, “DNas” and “CTCF” are average of gene expressions, nucleosome densities, and insulator in the interactions, respectively; “H3K4me1” and “H3K27ac” are enhancer markers; “H3K4me3” is a promoter maker; “H3K27me3” and “H3K9me3” are repressor markers. Here, “cn1”, “cn2”, “cn3”, and “cn4” are community number 1, 2, and 3 in a chromosome, respectively, which are the same numbers as the node labels in the network plots.

**SFigure 11.** **Heatmaps of intra-chromosomal community interactions for chromosomes 3, 17 and 20 after selecting significant intra-chromosomal interactions at 500kb resolution by HOMER in one hours E2 treated MCF7 cells.**

**
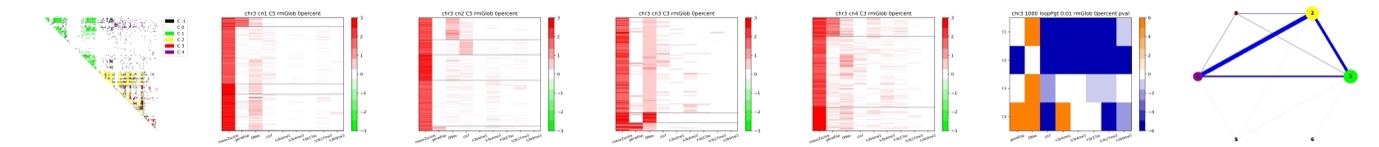
**

**
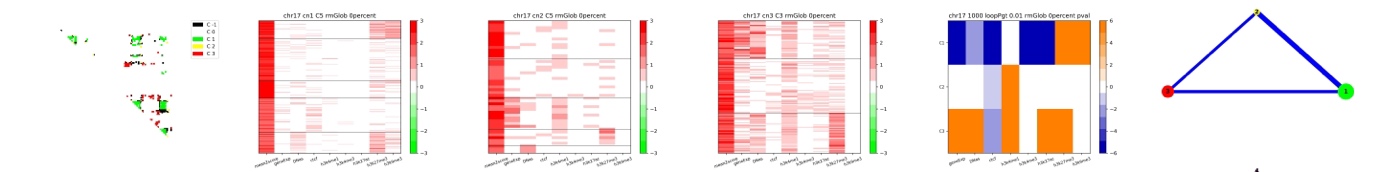
**

**
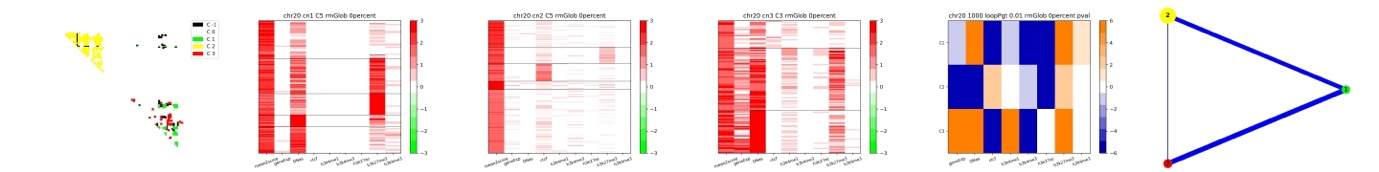
**

In each panel, *the left most heatmaps* are intra-chromosomal interaction matrices where interactions are colored by their community (or network cluster) labels. For example, black indicates two nodes of an edge (or interaction) do not belong to the same community, but the other colors represent the community label of an interaction. *The right most figures* are network plots of predicted intra-chromosomal community interactions (only show communities with more than 20 edges) in a chromosome, where the size of nodes and the width of edges indicates the number of edges (interactions) in the community and the number communications between the communities, respectively. The color of nodes indicates the community label. In a panel, *the second right most figures* are heatmaps of log10 transformed P-values for enrichment of a genomic feature in a community (or network cluster) against that from randomly generated (e.g., the number of P-value < 0.01 in 1000 times random permutation T-tests) intra-chromosomal interactions, where the orange and blue colors represent positive and negative enrichment of the feature, respectively. This sign of the enrichment is inferred from the expected T-values of random permutation T-test. The rest of red and green coded heatmaps are genomic features in intra-chromosomal community interactions: “Zscore” is the Z-score of intra-chromosomal interactions; “geneExp”, “DNas” and “CTCF” are average of gene expressions, nucleosome densities, and insulator in the interactions, respectively; “H3K4me1” and “H3K27ac” are enhancer markers; “H3K4me3” is a promoter maker; “H3K27me3” and “H3K9me3” are repressor markers. Here, “cn1”, “cn2”, “cn3”, and “cn4” are community number 1, 2, and 3 in a chromosome, respectively, which are the same numbers as the node labels in the network plots.

**SFigure 12. Statistical results for determining an optimal super resolution in HOMER with window bin size 50kb, 100kb, and 500kb**

**
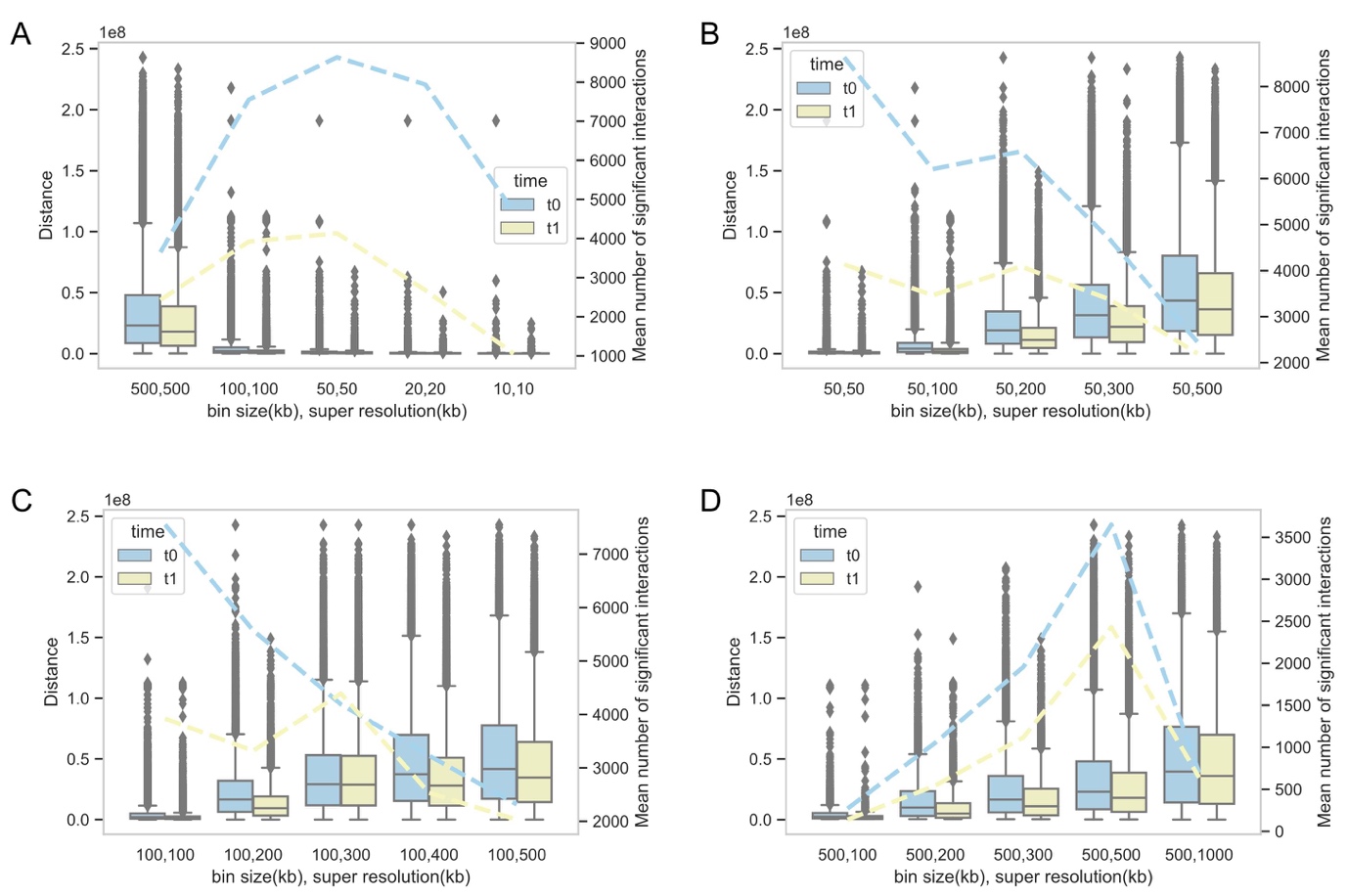
**

In the figure, box plots are the length of identified significant interactions from HOMER in twenty-three chromosomes. The dashed lines are the mean number of significant interactions in twenty-three chromosomes. An optimal choice is selected before both the length of significant interactions and the number of significant interactions are declined significantly. (A) Results of window bin size equals to super resolution, which is suggested by HOMER. (B) Results of window bin size equals to 50kb. (C) Results of window bin size equals to 100kb. (D) Results of window bin size equals to 500kb. Here, t0 and t1 represent untreated and one-hours E2-treated MCF7 cells, respectively.

**SFigure 13. Histograms for the number of edges in a community (or network cluster) with a cutoff value *p* = 0.02**.


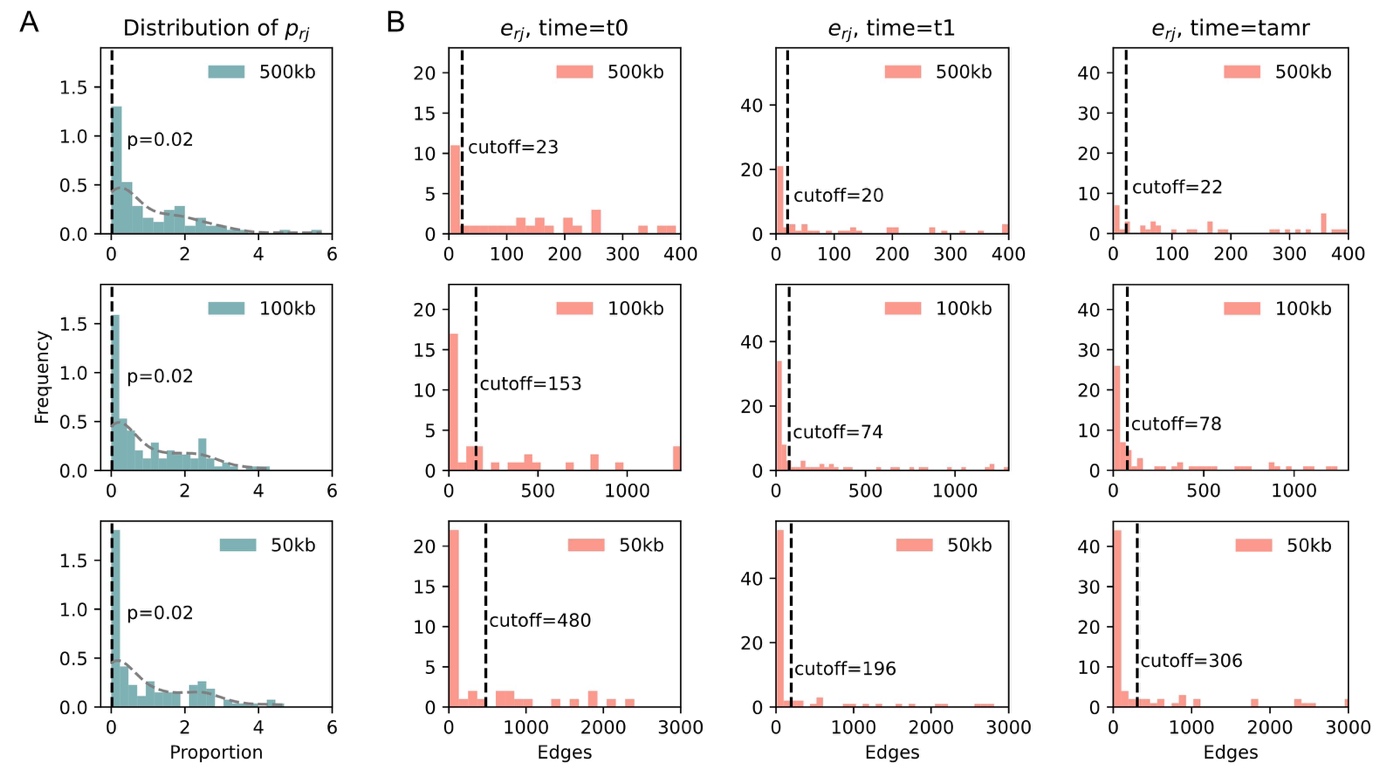


1. Histograms for the normalized proportion of edges in a cluster with a unified cutoff value *p* = 0.02 (black dash lines) under three different interaction resolutions (e.g., 500kb, 100kb, 50kb).
2. Histograms for the number of edges in a cluster with a cutoff value for the minimal number of edges in a valid community. Here, each column is the histogram plots for the number of edges in a community at a specific condition (e.g., t0, t1, and TAMR represents untreated, one-hours E2 treated MCF7 cells, and tamoxifen-resistant MCF7TR cell, respectively).

In the figure, each row of plots has the same interaction resolution (e.g., window bin size = 500kb, 100kb, and 50kb for the first, second, and third row, respectively).

**SFigure 14. Histograms for the number of edges in a community (or network cluster) with a cutoff value *p* = 0.01**.


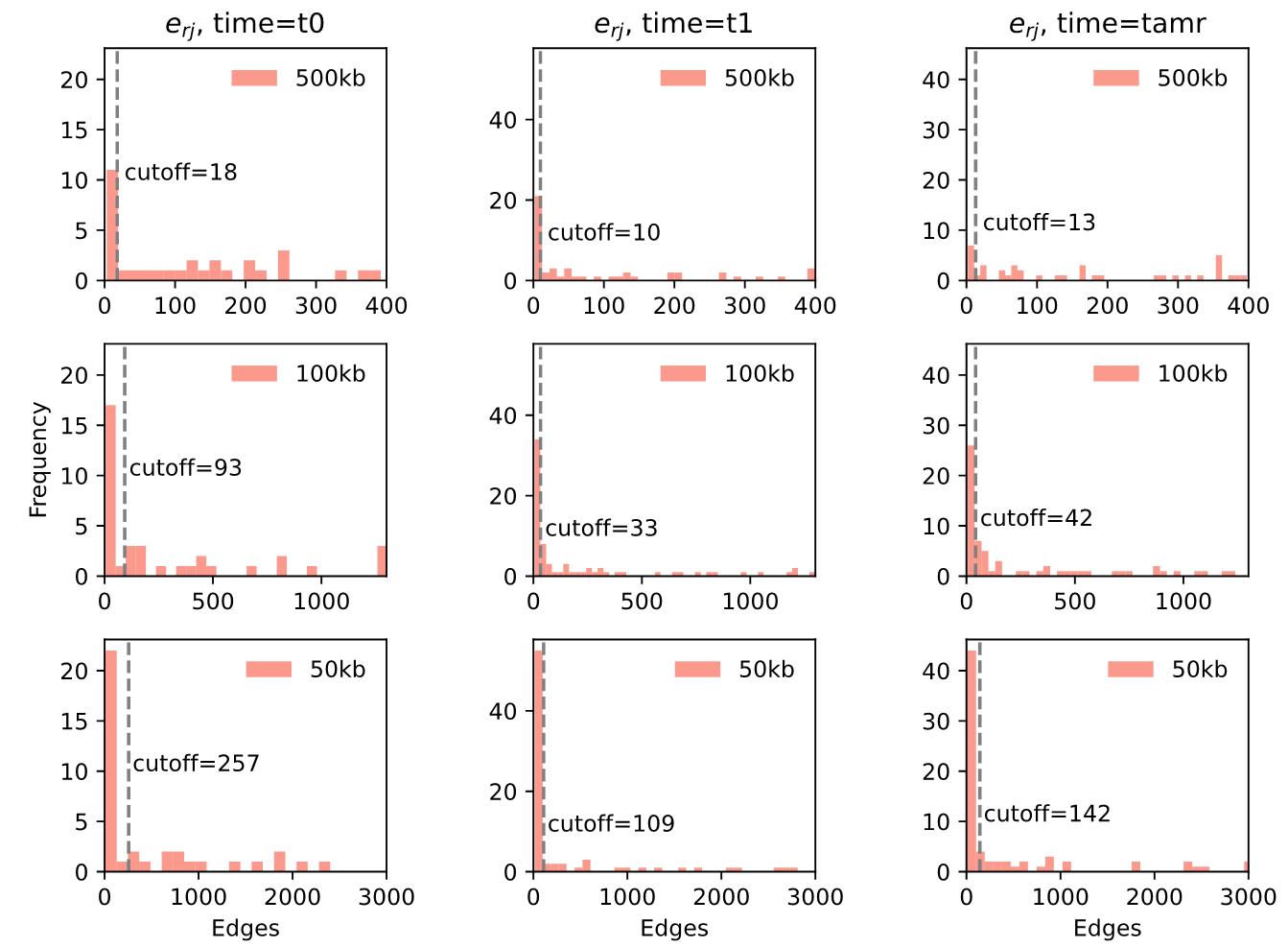


In the figure, the gray dashed line and the corresponding text indicate the cutoff value for minimal number of edges in a valid community (e.g., the corresponding proportion *p* = 0.01). In each column, the plot of light pink bars represents the distribution of the number of edges in a community from a specific condition (e.g., t0, t1, and TAMR for untreated, one-hours E2 treated MCF7 cells, and tamoxifen-resistant MCF7TR cell, respectively). In each row, the histograms are generated from the same interaction resolution (e.g., or window bin size = 500kb, 100kb, and 50kb for the first, second and third row, respectively).

**SFigure 15.** **Histograms for the number of edges in a community (or network cluster) with a cutoff value *p* = 0.05**.


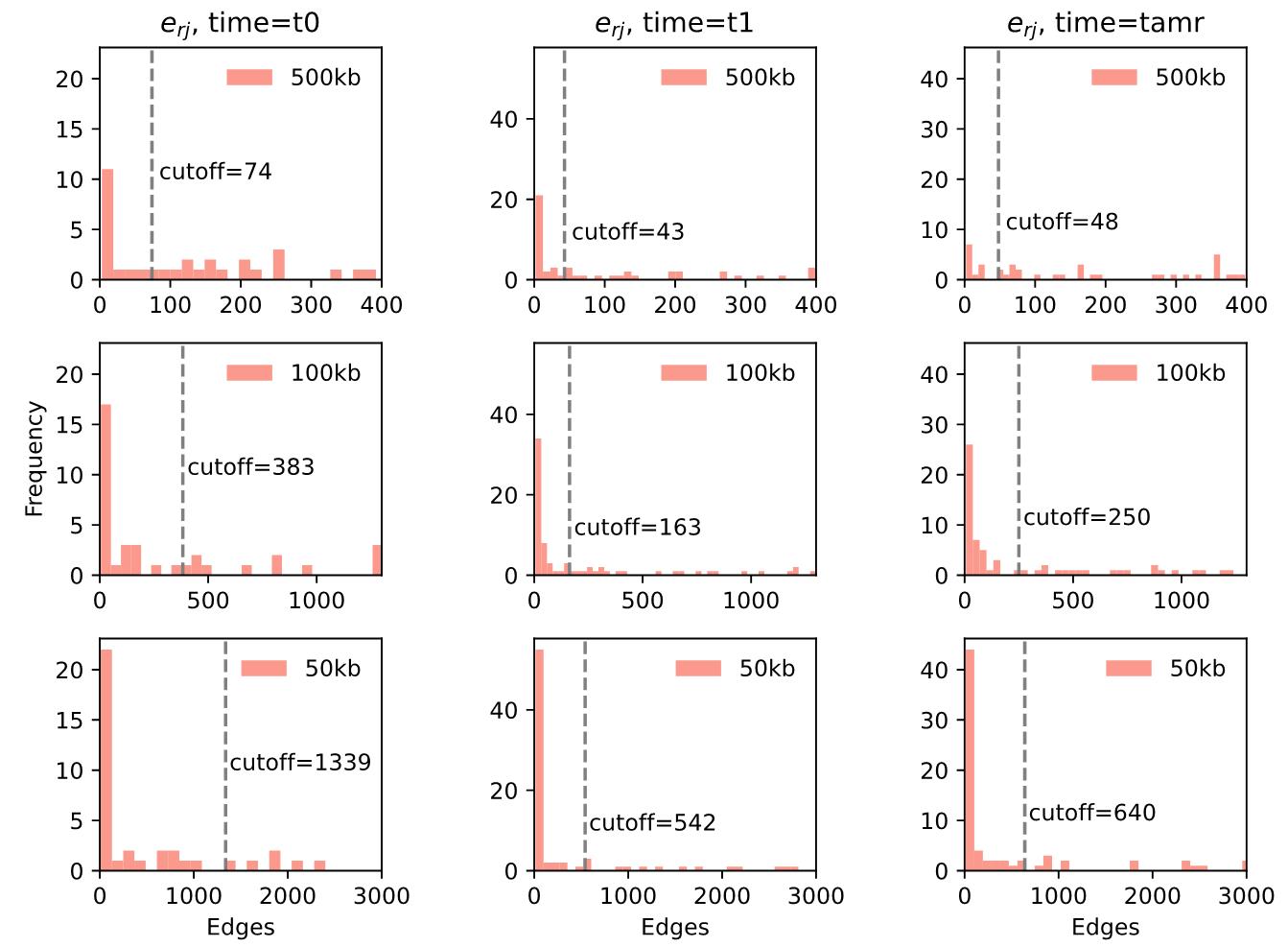


In the figure, the gray dashed line and the corresponding text indicate the cutoff value for minimal number of edges in a valid community (e.g., the corresponding proportion *p* = 0.05). In each column, the plot of light pink bars represents the distribution of the number of edges in a community from a specific condition (e.g., t0, t1, and TAMR for untreated, one-hours E2 treated MCF7 cells, and tamoxifen-resistant MCF7TR cell, respectively). In each row, the histograms are generated from the same interaction resolution (e.g., or window bin size = 500kb, 100kb, and 50kb for the first, second and third row, respectively).

**SFigure 16. Heatmaps of intra-chromosomal community interactions for chromosomes 3 at three different resolutions based on significant intra-chromosomal interactions identified by HOMER in untreated MCF7 cells.**

(A) The heatmaps of significant intra-chromosomal interactions identified by HOMER. (B) The heatmaps of color-coded community labels for intra-chromosomal interactions predicted by SLM algorithm, where the black and the other colors represent a pair of interactions (or two nodes with an edge) classified in the same and the different community, respectively. (C) The heatmaps of genomic feature enrichments in valid communities, where the minimum size of valid communities is 23, 153, and 480 for window bin size equals 500kb, 100kb, and 50kb, respectively. (D) Interactions of valid communities are represent by the super networks.

**SFigure 17. Heatmaps of intra-chromosomal community interactions for chromosomes 20 at three different resolutions based on significant intra-chromosomal interactions identified by HOMER in untreated MCF7 cells.**

(A) The heatmaps of significant intra-chromosomal interactions identified by HOMER. (B) The heatmaps of color-coded community labels for intra-chromosomal interactions predicted by SLM algorithm, where the black and the other colors represent a pair of interactions (or two nodes with an edge) classified in the same and the different community, respectively. (C) The heatmaps of genomic feature enrichments. In valid communities, where the minimum size of valid communities is 23, 153, and 480 for window bin size equals 500kb, 100kb, and 50kb, respectively. (D) Interactions of valid communities are represented by the super networks.

**SFigure 18. Heatmaps of intra-chromosomal community interactions for chromosomes 3 at three different resolutions based on significant intra-chromosomal interactions identified by HOMER in one-hours E2 treated MCF7 cells.**

(A) The heatmaps of significant intra-chromosomal interactions identified by HOMER. (B) The heatmaps of color-coded community labels for intra-chromosomal interactions predicted by SLM algorithm, where the black and the other colors represent a pair of interactions (or the two nodes with an edge) classified in the same and the different community, respectively. (C) The heatmaps of genomic feature enrichments in valid communities, where the minimum size of valid communities is 20, 74, and 196 for window bin size equals 500kb, 100kb, and 50kb, respectively. (D) Interactions of valid communities represented by the super networks.

**SFigure 19. Heatmaps of intra-chromosomal community interactions for chromosomes 17 at three different resolutions based on significant intra-chromosomal interactions identified by HOMER in one-hours E2 treated MCF7 cells.**

(A) The heatmaps of significant intra-chromosomal interactions identified by HOMER. (B) The heatmaps of color-coded community labels for intra-chromosomal interactions redicted by SLM algorithm, where the black and the other colors represent a pair of interactions (or the two nodes with an edge) classified in the same and the different community, respectively. (C) The heatmaps of genomic feature enrichments in valid communities, where the minimum size of valid communities is 20, 74, and 196 for window bin size equals 500kb, 100kb, and 50kb, respectively. (D) Interactions of valid communities are represented by the super networks.

**SFigure 20. Heatmaps of intra-chromosomal community interactions for chromosomes 20 at three different resolutions based on significant intra-chromosomal interactions identified by HOMER in one-hours E2 treated MCF7 cells.**

(A) The heatmaps of significant intra-chromosomal interactions identified by HOMER. (B) The heatmaps of color-coded community labels for intra-chromosomal interactions predicted by SLM algorithm, where the black and the other colors represent a pair of interactions (or the two nodes with an edge) classified in the same and the different community, respectively. (C) The heatmaps of genomic feature enrichments in valid communities, where the minimum size of valid communities is 20, 74, and 196 for window bin size equals 500kb, 100kb, and 50kb, respectively. (D) Interactions of valid communities are represented by the super networks.

**SFigure 21. Heatmaps of intra-chromosomal community interactions for chromosomes 3 at three different resolutions based on significant intra-chromosomal interactions identified by HOMER in tamoxifen-resistant MCF7TR cells.**

(A) The heatmaps of significant intra-chromosomal interactions identified by HOMER. (B) The heatmaps of color-coded community labels for intra-chromosomal interactions predicted by SLM algorithm, where the black and the other colors represent a pair of interactions (or the two nodes with an edge) classified in the same and the different community, respectively. (C) The heatmaps of genomic feature enrichments in valid communities, where the minimum size of valid communities is 22, 78, and 306 for window bin size equals 500kb, 100kb, and 50kb, respectively. (D) Interactions of valid communities are represented by the super networks.

**SFigure 22. Heatmaps of intra-chromosomal community interactions for chromosomes 17 at three different resolutions based on significant intra-chromosomal interactions identified by HOMER in tamoxifen-resistant MCF7TR cells.**

(A) The heatmaps of significant intra-chromosomal interactions identified by HOMER. (B) The heatmaps of color-coded community labels for intra-chromosomal interactions predicted by SLM algorithm, where the black and the other colors represent a pair of interactions (or the two nodes with an edge) classified in the same and the different community, respectively. (C) The heatmaps of genomic feature enrichments in valid communities, where the minimum size of valid communities is 22, 78, and 306 for window bin size equals 500kb, 100kb, and 50kb, respectively. (D) Interactions of valid communities are represented by the super networks.

**SFigure 23. Heatmaps of intra-chromosomal community interactions for chromosomes 20 at three different resolutions based on significant intra-chromosomal interactions identified by HOMER in tamoxifen-resistant MCF7TR cells.**

(A) The heatmaps of significant intra-chromosomal interactions identified by HOMER. (B) The heatmaps of color-coded community labels for intra-chromosomal interactions predicted by SLM algorithm, where the black and the other colors represent a pair of interactions (or the two nodes with an edge) classified in the same and the different community, respectively. (C) The heatmaps of genomic feature enrichments in valid communities, where the minimum size of valid communities is 22, 78, and 306 for window bin size equals 500kb, 100kb, and 50kb, respectively. (D) Interactions of valid communities are represented by the super networks.

**SFigure 24. Significantly interacting nodes selected by evaluating Euclidean distance of topological and genomic features at 500kb resolution (*p* < 0.05)**

**
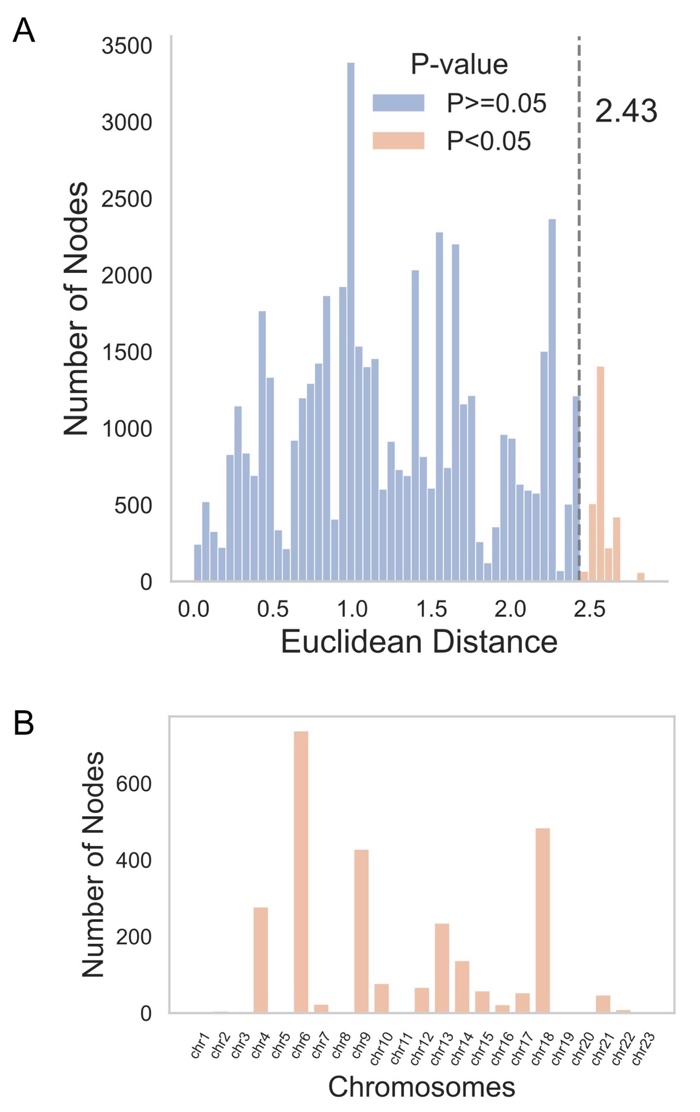
**

Differences in nodes in the network from one-hours E2 treated MCF7 cells and tamoxifen-resistant MCF7TR cells were computed and filtered based on Euclidean distance. (A) The Euclidean distances of all nodes were fitted to a Gaussian distribution. Network nodes with significant differences are screened out through p-value<0.05 (orange bar). (B) The distribution of selected nodes in different chromosomes.

**SFigure 25. Top 15 enriched functional annotations of gene ontology (GO) on 515 DIEGs**

Enrichment analysis of GO molecular function and biological process on 515 DIEGs obtained by DAVID online tool. The pink bar represents the Fold Enrichment (top axis), and the green scatters denotes the -log10 transformed P-value (bottom axis). The top 15 functional annotations are selected under the thresholds of Fold Enrichment >= 2 and p-value =< 0.05, and ranked by Fold Enrichment. The 515 DIEGs were filtered through both differential network analysis and Absolute(relative ratio)>0.66 from intra-chromosomal interaction network between MCF7 cells and tamoxifen-resistant MCF7TR cells.

**SFigure 26. Heatmaps of intra-chromosomal community interactions for chromosome 1 at 50 kb and 500 kb resolution in MCF7TR cells.**


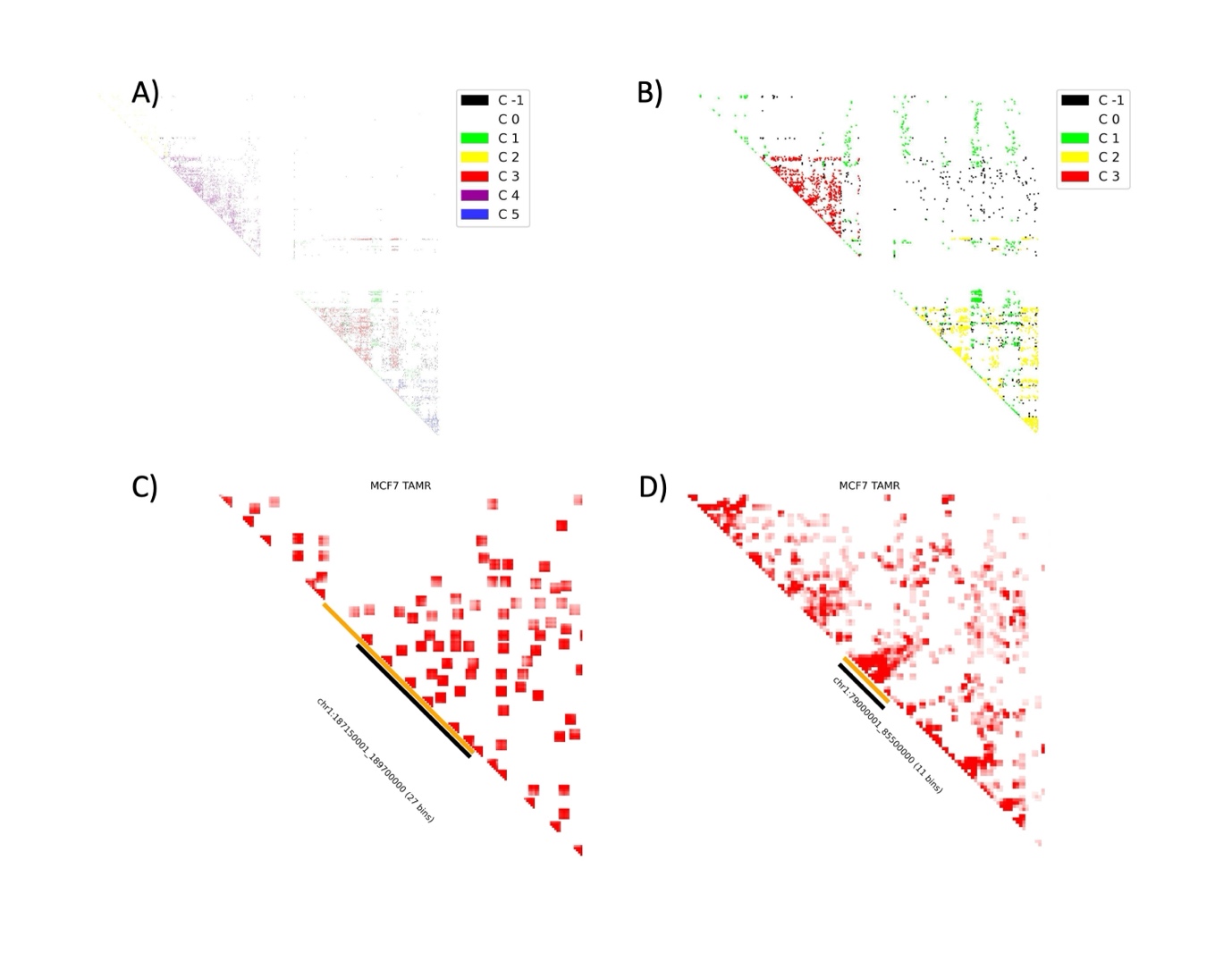


Figure A) and B) show heatmaps of identified valid network communities (or clusters) of intra-chromosomal interactions at chromosome 1 with window bin size 50 kb and 500 kb, respectively. The results obtained by applying DNAICI on Hi-C datasets from tamoxifen-resistant (TR) breast cancer cell line, MCF7TR (or MCF7 TAMR) cells. Figure C) and D) display heatmaps of selected topologically associating domain (TAD) from chromosome 1 with window bin size 50 kb and 500 kb, respectively. The TAD was obtained by applying TopDom on the same intra-chromosomal interaction matrices in Figures A) and B), respectively. In figures C) and D), the orange and black color bars indicate the positions of the selected TAD and the diagonal window bins at valid network communities, respectively.

**SFigure 27. Boxplot of the percentages of TAD or Gap with network clustered diagonal elements (window bins) in valid intra-chromosomal community interactions.**


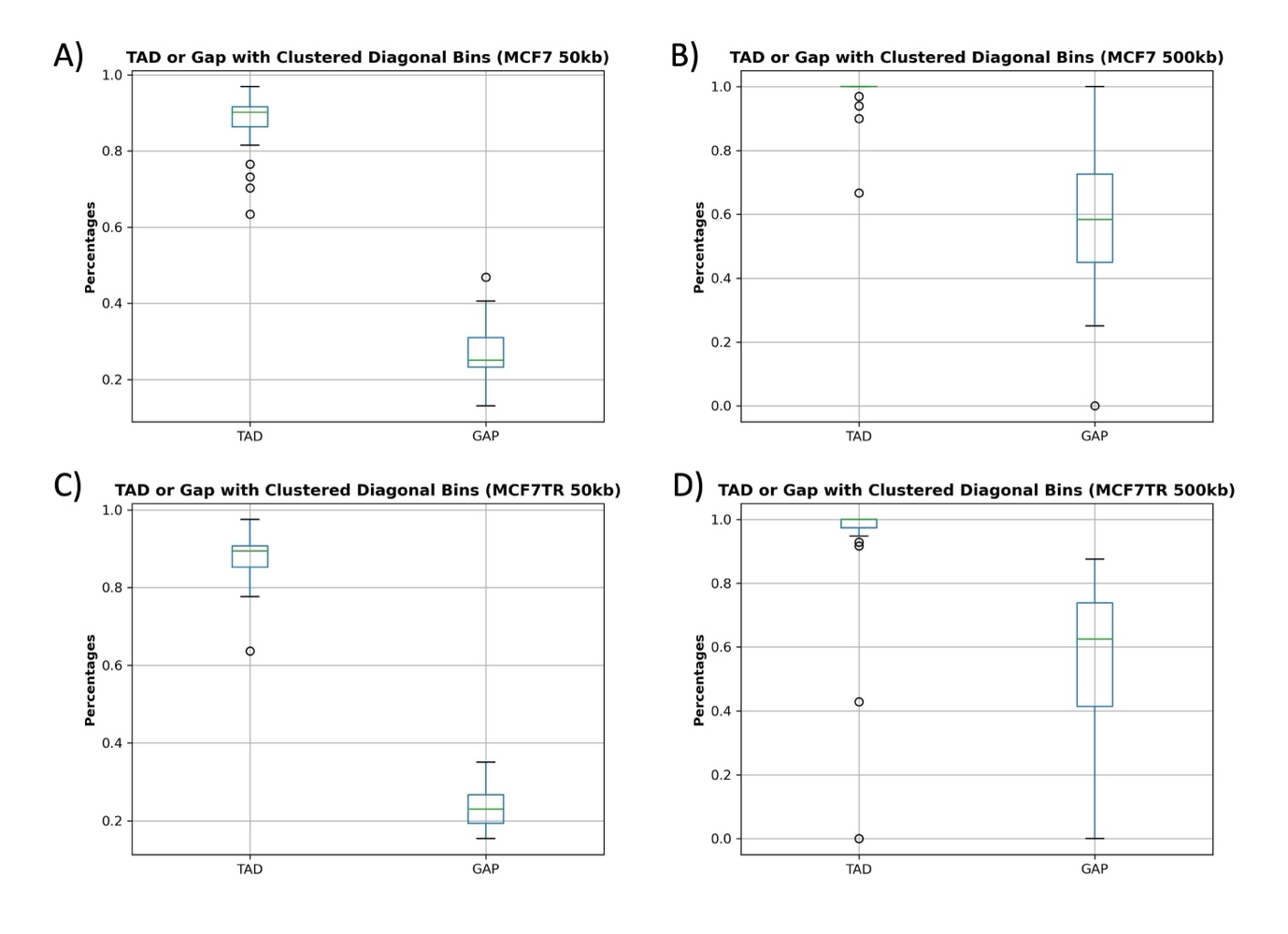


Figures A) and B) show boxplots of percentages of identified topologically associating domain (TAD) or Gap from TopDom in untreated MCF7 cell, which contain the diagonal window bins of predicted valid intra-chromosomal community interactions based on DNAICI, at 50 kb and 500 kb window bin size, respectively. Figures C) and D) illustrate boxplots of percentages of identified topologically associating domain (TAD) or Gap from TopDom in tamoxifen-resistant (TR) breast cancer cell line (MCF7TR or MCF7 TAMR), which contain diagonal window bins of predicted valid intra-chromosomal community interactions based on DNAICI, at 50 kb and 500 kb window bin size, respectively.

**SFigure 28. Percentages of window bins in intra-chromosomal communities are A/B compartments in MCF7TR cells.**

**
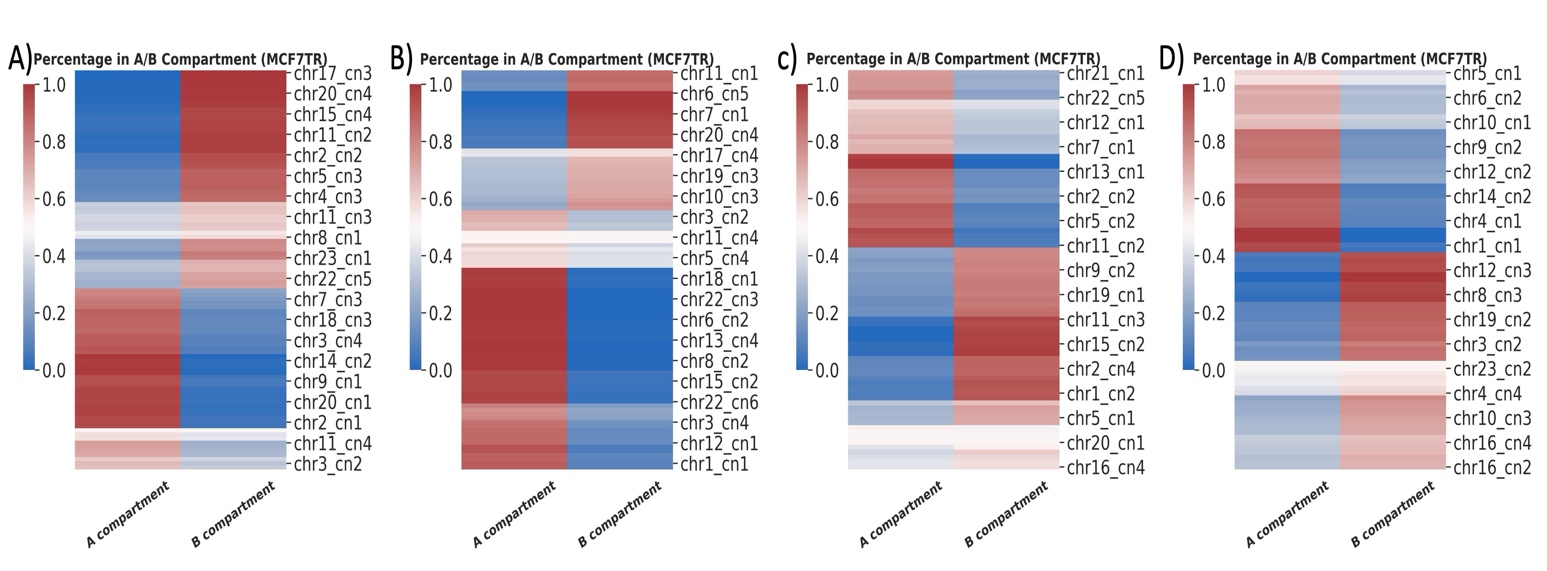
**

Figures A) and B) show heatmaps of percentages of window bins (50 kb resolution) in valid intra-chromosomal communities (MCF7TR cells) belong to predicted A/B compartments by HOMER[5] and FAN-C[6], respectively. Figures C) and D) are heatmaps of percentages of window bins (500 kb resolution) in valid intra-chromosomal communities (MCF7TR cells) belong to predicted A/B compartments by HOMER and FAN-C, respectively. The valid intra-chromosomal community interactions were identified by applying DNAICI on Hi-C data from tamoxifen-resistant (TR) breast cancer cells (MCF7TR or MCF7 TAMR) at 50 kb and 500 kb resolution, respectively.

**Supplementary Tables**

**STable 1. Summary of intra-chromosomal community interactions after selecting significant intra-chromosomal interactions in Hi-C data of untreated MCF7 cells by HOMER**

|  | Edges | Modularity | Edges_VC | Valid clusters | Clusters | %Edges_VC |
| --- | --- | --- | --- | --- | --- | --- |
| chr1 | 9948 | 0.46 | 7992 | 3 | 58 | 0.80 |
| chr2 | 24128 | 0.36 | 17373 | 3 | 16 | 0.72 |
| chr3 | 14524 | 0.35 | 10154 | 4 | 11 | 0.70 |
| chr4 | 13207 | 0.36 | 9439 | 3 | 12 | 0.72 |
| chr5 | 14910 | 0.34 | 10604 | 3 | 11 | 0.71 |
| chr6 | 11431 | 0.30 | 8478 | 4 | 16 | 0.74 |
| chr7 | 9158 | 0.40 | 7064 | 4 | 14 | 0.77 |
| chr8 | 9276 | 0.28 | 5654 | 4 | 30 | 0.61 |
| chr9 | 6709 | 0.29 | 4761 | 3 | 66 | 0.71 |
| chr10 | 7021 | 0.34 | 5208 | 3 | 11 | 0.74 |
| chr11 | 7800 | 0.29 | 5090 | 4 | 12 | 0.65 |
| chr12 | 6849 | 0.31 | 4320 | 4 | 11 | 0.63 |
| chr13 | 4918 | 0.28 | 2943 | 4 | 42 | 0.60 |
| chr14 | 6036 | 0.27 | 4266 | 3 | 43 | 0.71 |
| chr15 | 1933 | 0.43 | 1537 | 3 | 49 | 0.80 |
| chr16 | 2018 | 0.45 | 1472 | 4 | 25 | 0.73 |
| chr17 | 1062 | 0.39 | 931 | 2 | 55 | 0.88 |
| chr18 | 4721 | 0.23 | 3578 | 2 | 10 | 0.76 |
| chr19 | 1187 | 0.32 | 888 | 3 | 24 | 0.75 |
| chr20 | 852 | 0.38 | 655 | 3 | 41 | 0.77 |
| chr21 | 563 | 0.22 | 353 | 2 | 40 | 0.63 |
| chr22 | 252 | 0.56 | 207 | 4 | 43 | 0.82 |
| chr23 | 12935 | 0.31 | 8883 | 3 | 18 | 0.69 |

Here, smart local moving algorithm[7] was applied on an intra-chromosomal interaction matrix for predicting the intra-chromosomal community interactions in each chromosome, respectively. The optimal number of communities is accessed by standard modularity score[8]. “Edges” are the number of edges selected by HOMER. “Modularity” is the standard modularity score of predicted intra-chromosomal community interactions. “Valid clusters” and “Clusters” are valid and total number of network clusters (or intra-chromosomal community), respectively. A “Valid cluster” means a network cluster (intra-chromosomal community) has at least 20 edges. “Edges_VC” and “%Edges_VC” represents the number of and the percentage of “Edges_AF” in valid clusters, respectively.

**STable 2. Summary of intra-chromosomal community interactions after removing the lowest 5 percentages of intra-chromosomal interactions in Hi-C data of untreated MCF7 cells.**

|  | Edges | Edges_AF | Minimum | %Edges_AF | Modularity | Edges_VC | Valid clusters | Clustesrs | %Edges_VC |
| --- | --- | --- | --- | --- | --- | --- | --- | --- | --- |
| chr1 | 70377 | 66471 | -1.06 | 0.94 | 0.11 | 30423 | 3 | 45 | 0.46 |
| chr2 | 93935 | 91589 | -1.06 | 0.98 | 0.07 | 52274 | 2 | 9 | 0.57 |
| chr3 | 64694 | 62837 | -1.06 | 0.97 | 0.05 | 24844 | 3 | 8 | 0.40 |
| chr4 | 58393 | 56706 | -1.06 | 0.97 | 0.06 | 23727 | 3 | 8 | 0.42 |
| chr5 | 55647 | 54516 | -1.06 | 0.98 | 0.04 | 29698 | 2 | 7 | 0.55 |
| chr6 | 42713 | 41265 | -1.06 | 0.97 | 0.06 | 21216 | 3 | 14 | 0.51 |
| chr7 | 39115 | 36692 | -1.06 | 0.94 | 0.07 | 15201 | 3 | 8 | 0.41 |
| chr8 | 33055 | 31246 | -1.06 | 0.95 | 0.08 | 21928 | 2 | 7 | 0.70 |
| chr9 | 22163 | 20565 | -1.06 | 0.93 | 0.08 | 12073 | 2 | 37 | 0.59 |
| chr10 | 31913 | 30151 | -1.06 | 0.95 | 0.04 | 16617 | 2 | 7 | 0.55 |
| chr11 | 28670 | 26233 | -1.06 | 0.92 | 0.10 | 16071 | 2 | 7 | 0.61 |
| chr12 | 31176 | 29852 | -1.06 | 0.96 | 0.04 | 16381 | 2 | 7 | 0.55 |
| chr13 | 17584 | 16785 | -1.06 | 0.96 | 0.04 | 9107 | 2 | 40 | 0.54 |
| chr14 | 15222 | 14690 | -1.06 | 0.97 | 0.03 | 7825 | 2 | 40 | 0.53 |
| chr15 | 12081 | 10805 | -1.06 | 0.89 | 0.07 | 6369 | 2 | 42 | 0.59 |
| chr16 | 10473 | 8979 | -1.06 | 0.86 | 0.10 | 5528 | 2 | 23 | 0.62 |
| chr17 | 10858 | 8883 | -1.06 | 0.82 | 0.09 | 5348 | 2 | 7 | 0.60 |
| chr18 | 10833 | 10180 | -1.06 | 0.94 | 0.04 | 5629 | 2 | 8 | 0.55 |
| chr19 | 5556 | 3851 | -1.06 | 0.69 | 0.11 | 2425 | 2 | 7 | 0.63 |
| chr20 | 6663 | 5749 | -1.06 | 0.86 | 0.08 | 3390 | 2 | 7 | 0.59 |
| chr21 | 2376 | 1953 | -1.06 | 0.82 | 0.10 | 1197 | 2 | 27 | 0.61 |
| chr22 | 2305 | 1586 | -1.06 | 0.69 | 0.09 | 959 | 2 | 35 | 0.61 |
| chrX | 39763 | 38751 | -1.06 | 0.98 | 0.04 | 21234 | 2 | 12 | 0.55 |

Here, smart local moving algorithm[7] was applied on an intra-chromosomal interaction matrix for predicting the intra-chromosomal community interactions in each chromosome, respectively. The optimal number of communities is accessed by standard modularity score[8]. “Edges”, “Edges_AF”, and “%Edges_AF” are the number of edges before and after filtering 5% of the lowest intra-chromosomal interactions, and the percentages of edges passed filtering. “Minimum” is the minimum Z-score of an edge in an intra-chromosomal interaction matrix. “Modularity” is the standard modularity score of predicted intra-chromosomal community interactions. “Valid clusters” and “Clusters” are valid and total number of network clusters (or intra-chromosomal community), respectively. A “Valid cluster” means a network cluster (intra-chromosomal community) has at least 20 edges. “Edges_VC” and “%Edges_VC” represents the number of and the percentage of “Edges_AF” in valid clusters, respectively.

**STable 3. Summary of intra-chromosomal community interactions after removing the lowest 10 percentages of intra-chromosomal interactions in Hi-C data of untreated MCF7 cells.**

|  | Edges | Edges_AF | Minimum | %Edges_AF | Modularity | Edges_VC | Valid clusters | Clustesrs | %Edges_VC |
| --- | --- | --- | --- | --- | --- | --- | --- | --- | --- |
| chr1 | 70377 | 62404 | -0.95 | 0.89 | 0.12 | 29168 | 3 | 45 | 0.47 |
| chr2 | 93935 | 88615 | -0.95 | 0.94 | 0.08 | 51462 | 2 | 9 | 0.58 |
| chr3 | 64694 | 60532 | -0.95 | 0.94 | 0.06 | 34358 | 2 | 7 | 0.57 |
| chr4 | 58393 | 54549 | -0.95 | 0.93 | 0.06 | 21868 | 3 | 8 | 0.40 |
| chr5 | 55647 | 52911 | -0.95 | 0.95 | 0.05 | 29359 | 2 | 7 | 0.56 |
| chr6 | 42713 | 39602 | -0.95 | 0.93 | 0.07 | 20707 | 3 | 15 | 0.52 |
| chr7 | 39115 | 34385 | -0.95 | 0.88 | 0.08 | 14960 | 3 | 8 | 0.44 |
| chr8 | 33055 | 29349 | -0.95 | 0.89 | 0.09 | 21302 | 2 | 7 | 0.73 |
| chr9 | 22163 | 19257 | -0.95 | 0.87 | 0.09 | 11216 | 2 | 42 | 0.58 |
| chr10 | 31913 | 28403 | -0.95 | 0.89 | 0.06 | 15045 | 3 | 8 | 0.53 |
| chr11 | 28670 | 24047 | -0.95 | 0.84 | 0.12 | 15331 | 2 | 7 | 0.64 |
| chr12 | 31176 | 28458 | -0.95 | 0.91 | 0.06 | 16000 | 2 | 7 | 0.56 |
| chr13 | 17584 | 15813 | -0.95 | 0.90 | 0.06 | 8888 | 2 | 40 | 0.56 |
| chr14 | 15222 | 14059 | -0.95 | 0.92 | 0.04 | 7742 | 2 | 40 | 0.55 |
| chr15 | 12081 | 9808 | -0.95 | 0.81 | 0.11 | 6132 | 2 | 42 | 0.63 |
| chr16 | 10473 | 7811 | -0.95 | 0.75 | 0.13 | 5093 | 2 | 23 | 0.65 |
| chr17 | 10858 | 7650 | -0.95 | 0.71 | 0.12 | 4928 | 2 | 7 | 0.64 |
| chr18 | 10833 | 9521 | -0.95 | 0.88 | 0.07 | 5497 | 2 | 8 | 0.58 |
| chr19 | 5556 | 3092 | -0.95 | 0.56 | 0.16 | 1664 | 3 | 8 | 0.54 |
| chr20 | 6663 | 5110 | -0.95 | 0.77 | 0.11 | 3165 | 2 | 7 | 0.62 |
| chr21 | 2376 | 1754 | -0.95 | 0.74 | 0.14 | 1152 | 2 | 27 | 0.66 |
| chr22 | 2305 | 1229 | -0.95 | 0.53 | 0.13 | 700 | 2 | 36 | 0.57 |
| chrX | 39763 | 37170 | -0.95 | 0.94 | 0.05 | 14859 | 3 | 13 | 0.40 |

Here, smart local moving algorithm[7] was applied on an intra-chromosomal interaction matrix for predicting the intra-chromosomal community interactions in each chromosome, respectively. The optimal number of communities is accessed by standard modularity score[8]. “Edges”, “Edges_AF”, and “%Edges_AF” are the number of edges before and after filtering 10% of the lowest intra-chromosomal interactions, and the percentages of edges passed filtering. “Minimum” is the minimum Z-score of an edge in an intra-chromosomal interaction matrix. “Modularity” is the standard modularity score of predicted intra-chromosomal community interactions. “Valid clusters” and “Clusters” are valid and total number of network clusters (or intra-chromosomal community), respectively. A “Valid cluster” means a network cluster (intra-chromosomal community) has at least 20 edges. “Edges_VC” and “%Edges_VC” represents the number of and the percentage of “Edges_AF” in valid clusters, respectively.

**STable 4. Summary of intra-chromosomal community interactions after removing the lowest 20 percentages of intra-chromosomal interactions in Hi-C data of untreated MCF7 cells.**

|  | Edges | Edges_AF | Minimum | %Edges_AF | Modularity | Edges_VC | Valid Clusters | Clustesrs | %Edges_VC |
| --- | --- | --- | --- | --- | --- | --- | --- | --- | --- |
| chr1 | 70377 | 54144 | -0.77 | 0.77 | 0.15 | 34263 | 3 | 45 | 0.63 |
| chr2 | 93935 | 81674 | -0.77 | 0.87 | 0.10 | 49193 | 2 | 9 | 0.60 |
| chr3 | 64694 | 55150 | -0.77 | 0.85 | 0.08 | 32274 | 2 | 7 | 0.59 |
| chr4 | 58393 | 48614 | -0.77 | 0.83 | 0.08 | 27490 | 3 | 8 | 0.57 |
| chr5 | 55647 | 48671 | -0.77 | 0.88 | 0.07 | 28113 | 2 | 8 | 0.58 |
| chr6 | 42713 | 35836 | -0.77 | 0.84 | 0.10 | 19838 | 3 | 15 | 0.55 |
| chr7 | 39115 | 29622 | -0.77 | 0.76 | 0.11 | 14624 | 3 | 9 | 0.49 |
| chr8 | 33055 | 25599 | -0.77 | 0.77 | 0.12 | 13165 | 3 | 8 | 0.51 |
| chr9 | 22163 | 16909 | -0.77 | 0.76 | 0.13 | 10617 | 2 | 47 | 0.63 |
| chr10 | 31913 | 24668 | -0.77 | 0.77 | 0.09 | 14320 | 2 | 8 | 0.58 |
| chr11 | 28670 | 20328 | -0.77 | 0.71 | 0.16 | 13605 | 2 | 7 | 0.67 |
| chr12 | 31176 | 25258 | -0.77 | 0.81 | 0.09 | 15015 | 2 | 7 | 0.59 |
| chr13 | 17584 | 13922 | -0.77 | 0.79 | 0.09 | 8288 | 2 | 40 | 0.60 |
| chr14 | 15222 | 13063 | -0.77 | 0.86 | 0.07 | 7557 | 2 | 40 | 0.58 |
| chr15 | 12081 | 8358 | -0.77 | 0.69 | 0.15 | 5398 | 2 | 43 | 0.65 |
| chr16 | 10473 | 6234 | -0.77 | 0.60 | 0.17 | 3383 | 3 | 24 | 0.54 |
| chr17 | 10858 | 5928 | -0.77 | 0.55 | 0.17 | 4023 | 2 | 7 | 0.68 |
| chr18 | 10833 | 8567 | -0.77 | 0.79 | 0.10 | 5237 | 2 | 8 | 0.61 |
| chr19 | 5556 | 2236 | -0.77 | 0.40 | 0.23 | 1339 | 3 | 8 | 0.60 |
| chr20 | 6663 | 4107 | -0.77 | 0.62 | 0.15 | 2718 | 2 | 7 | 0.66 |
| chr21 | 2376 | 1408 | -0.77 | 0.59 | 0.19 | 1001 | 2 | 29 | 0.71 |
| chr22 | 2305 | 737 | -0.77 | 0.32 | 0.20 | 435 | 3 | 36 | 0.59 |
| chrX | 39763 | 33420 | -0.77 | 0.84 | 0.07 | 13840 | 3 | 13 | 0.41 |

Here, smart local moving algorithm[7] was applied on an intra-chromosomal interaction matrix for predicting the intra-chromosomal community interactions in each chromosome, respectively. The optimal number of communities is accessed by standard modularity score[8]. “Edges”, “Edges_AF”, and “%Edges_AF” are the number of edges before and after filtering 20% of the lowest intra-chromosomal interactions, and the percentages of edges passed filtering. “Minimum” is the minimum Z-score of an edge in an intra-chromosomal interaction matrix. “Modularity” is the standard modularity score of predicted intra-chromosomal community interactions. “Valid clusters” and “Clusters” are valid and total number of network clusters (or intra-chromosomal community), respectively. A “Valid cluster” means a network cluster (intra-chromosomal community) has at least 20 edges. “Edges_VC” and “%Edges_VC” represents the number of and the percentage of “Edges_AF” in valid clusters, respectively.

**STable 5. Summary of intra-chromosomal community interactions after removing the lowest 40 percentages of intra-chromosomal interactions in Hi-C data of untreated MCF7 cells.**

|  | Edges | Edges_AF | Minimum | %Edges_AF | Modularity | Edges_VC | Valid clusters | Clustesrs | %Edges_VC |
| --- | --- | --- | --- | --- | --- | --- | --- | --- | --- |
| hr1 | 70377 | 32856 | -0.47 | 0.47 | 0.24 | 19326 | 3 | 47 | 0.59 |
| chr2 | 93935 | 60612 | -0.47 | 0.65 | 0.17 | 31939 | 3 | 10 | 0.53 |
| chr3 | 64694 | 42000 | -0.47 | 0.65 | 0.14 | 20179 | 3 | 8 | 0.48 |
| chr4 | 58393 | 35767 | -0.47 | 0.61 | 0.15 | 17999 | 3 | 9 | 0.50 |
| chr5 | 55647 | 39034 | -0.47 | 0.70 | 0.12 | 18407 | 3 | 11 | 0.47 |
| chr6 | 42713 | 28758 | -0.47 | 0.67 | 0.14 | 18009 | 3 | 17 | 0.63 |
| chr7 | 39115 | 22222 | -0.47 | 0.57 | 0.17 | 12581 | 3 | 9 | 0.57 |
| chr8 | 33055 | 20121 | -0.47 | 0.61 | 0.18 | 11770 | 3 | 10 | 0.59 |
| chr9 | 22163 | 13234 | -0.47 | 0.60 | 0.18 | 9090 | 2 | 52 | 0.69 |
| chr10 | 31913 | 19008 | -0.47 | 0.60 | 0.15 | 12537 | 2 | 8 | 0.66 |
| chr11 | 28670 | 15133 | -0.47 | 0.53 | 0.20 | 10862 | 2 | 7 | 0.72 |
| chr12 | 31176 | 19963 | -0.47 | 0.64 | 0.13 | 12059 | 3 | 8 | 0.60 |
| chr13 | 17584 | 10625 | -0.47 | 0.60 | 0.15 | 7024 | 2 | 40 | 0.66 |
| chr14 | 15222 | 11303 | -0.47 | 0.74 | 0.12 | 7074 | 2 | 40 | 0.63 |
| chr15 | 12081 | 6188 | -0.47 | 0.51 | 0.21 | 3925 | 3 | 45 | 0.63 |
| chr16 | 10473 | 4353 | -0.47 | 0.42 | 0.24 | 2642 | 3 | 24 | 0.61 |
| chr17 | 10858 | 3925 | -0.47 | 0.36 | 0.23 | 2517 | 3 | 9 | 0.64 |
| chr18 | 10833 | 6712 | -0.47 | 0.62 | 0.17 | 4555 | 2 | 8 | 0.68 |
| chr19 | 5556 | 1406 | -0.47 | 0.25 | 0.31 | 977 | 3 | 9 | 0.70 |
| chr20 | 6663 | 2847 | -0.47 | 0.43 | 0.21 | 1741 | 3 | 8 | 0.61 |
| chr21 | 2376 | 961 | -0.47 | 0.40 | 0.23 | 733 | 2 | 29 | 0.76 |
| chr22 | 2305 | 295 | -0.47 | 0.13 | 0.33 | 192 | 4 | 42 | 0.65 |
| chrX | 39763 | 26145 | -0.47 | 0.66 | 0.12 | 12829 | 3 | 13 | 0.49 |

Here, smart local moving algorithm[7] was applied on an intra-chromosomal interaction matrix for predicting the intra-chromosomal community interactions in each chromosome, respectively. The optimal number of communities is accessed by standard modularity score[8]. “Edges”, “Edges_AF”, and “%Edges_AF” are the number of edges before and after filtering 40% of the lowest intra-chromosomal interactions, and the percentages of edges passed filtering. “Minimum” is the minimum Z-score of an edge in an intra-chromosomal interaction matrix. “Modularity” is the standard modularity score of predicted intra-chromosomal community interactions. “Valid clusters” and “Clusters” are valid and total number of network clusters (or intra-chromosomal community), respectively. A “Valid cluster” means a network cluster (intra-chromosomal community) has at least 20 edges. “Edges_VC” and “%Edges_VC” represents the number of and the percentage of “Edges_AF” in valid clusters, respectively.

**STable 6. Summary of intra-chromosomal community interactions after removing the lowest 60 percentages of intra-chromosomal interactions in Hi-C data of untreated MCF7 cells.**

|  | Edges | Edges_AF | Minimum | %Edges_AF | Modularity | Edges_VC | Valid clusters | Clustesrs | %Edges_VC |
| --- | --- | --- | --- | --- | --- | --- | --- | --- | --- |
| chr1 | 70377 | 20448 | 0.00 | 0.29 | 0.27 | 12724 | 3 | 48 | 0.62 |
| chr2 | 93935 | 44480 | 0.00 | 0.47 | 0.22 | 30860 | 3 | 10 | 0.69 |
| chr3 | 64694 | 29708 | 0.00 | 0.46 | 0.20 | 16013 | 3 | 9 | 0.54 |
| chr4 | 58393 | 22360 | 0.00 | 0.38 | 0.22 | 13390 | 3 | 9 | 0.60 |
| chr5 | 55647 | 27727 | 0.00 | 0.50 | 0.19 | 15224 | 3 | 12 | 0.55 |
| chr6 | 42713 | 19593 | 0.00 | 0.46 | 0.20 | 13319 | 3 | 18 | 0.68 |
| chr7 | 39115 | 14915 | 0.00 | 0.38 | 0.24 | 9680 | 3 | 9 | 0.65 |
| chr8 | 33055 | 14451 | 0.00 | 0.44 | 0.24 | 9421 | 3 | 11 | 0.65 |
| chr9 | 22163 | 8904 | 0.00 | 0.40 | 0.25 | 6672 | 2 | 57 | 0.75 |
| chr10 | 31913 | 11339 | 0.00 | 0.36 | 0.24 | 8305 | 2 | 9 | 0.73 |
| chr11 | 28670 | 9059 | 0.00 | 0.32 | 0.26 | 6133 | 3 | 8 | 0.68 |
| chr12 | 31176 | 12713 | 0.00 | 0.41 | 0.19 | 8898 | 2 | 8 | 0.70 |
| chr13 | 17584 | 6002 | 0.00 | 0.34 | 0.25 | 4364 | 2 | 41 | 0.73 |
| chr14 | 15222 | 8428 | 0.00 | 0.55 | 0.19 | 5906 | 2 | 40 | 0.70 |
| chr15 | 12081 | 3352 | 0.00 | 0.28 | 0.29 | 2197 | 3 | 46 | 0.66 |
| chr16 | 10473 | 2368 | 0.00 | 0.23 | 0.31 | 1601 | 3 | 24 | 0.68 |
| chr17 | 10858 | 2149 | 0.00 | 0.20 | 0.31 | 1419 | 3 | 11 | 0.66 |
| chr18 | 10833 | 4270 | 0.00 | 0.39 | 0.26 | 3282 | 2 | 8 | 0.77 |
| chr19 | 5556 | 768 | 0.00 | 0.14 | 0.30 | 637 | 3 | 24 | 0.83 |
| chr20 | 6663 | 1575 | 0.00 | 0.24 | 0.28 | 1012 | 3 | 8 | 0.64 |
| chr21 | 2376 | 501 | 0.00 | 0.21 | 0.28 | 381 | 3 | 34 | 0.76 |
| chr22 | 2305 | 64 | 0.00 | 0.03 | 0.47 | 20 | 1 | 73 | 0.31 |
| chrX | 39763 | 17210 | 0.00 | 0.43 | 0.18 | 9505 | 3 | 15 | 0.55 |

Here, smart local moving algorithm[7] was applied on an intra-chromosomal interaction matrix for predicting the intra-chromosomal community interactions in each chromosome, respectively. The optimal number of communities is accessed by standard modularity score[8]. “Edges”, “Edges_AF”, and “%Edges_AF” are the number of edges before and after filtering 60% of the lowest intra-chromosomal interactions, and the percentages of edges passed filtering. “Minimum” is the minimum Z-score of an edge in an intra-chromosomal interaction matrix. “Modularity” is the standard modularity score of predicted intra-chromosomal community interactions. “Valid clusters” and “Clusters” are valid and total number of network clusters (or intra-chromosomal community), respectively. A “Valid cluster” means a network cluster (intra-chromosomal community) has at least 20 edges. “Edges_VC” and “%Edges_VC” represents the number of and the percentage of “Edges_AF” in valid clusters, respectively.

**STable 7. Summary of intra-chromosomal community interactions after removing the lowest 5 percentages of intra-chromosomal interactions in Hi-C data of one hours E2 treated MCF7 cells.**

|  | Edges | Edges_AF | Minimum | %Edges_AF | Modularity | Edges_VC | Valid clusters | Clustesrs | %Edges_VC |
| --- | --- | --- | --- | --- | --- | --- | --- | --- | --- |
| chr1 | 61196 | 57517 | -1.11 | 0.94 | 0.14 | 28220 | 3 | 45 | 0.49 |
| chr2 | 86285 | 84382 | -1.11 | 0.98 | 0.08 | 39188 | 3 | 10 | 0.46 |
| chr3 | 60075 | 58487 | -1.11 | 0.97 | 0.07 | 33855 | 2 | 7 | 0.58 |
| chr4 | 53397 | 51895 | -1.11 | 0.97 | 0.09 | 30932 | 2 | 7 | 0.60 |
| chr5 | 52038 | 51030 | -1.11 | 0.98 | 0.06 | 29037 | 2 | 7 | 0.57 |
| chr6 | 40104 | 39007 | -1.11 | 0.97 | 0.07 | 20829 | 3 | 13 | 0.53 |
| chr7 | 35385 | 33188 | -1.11 | 0.94 | 0.09 | 21693 | 2 | 7 | 0.65 |
| chr8 | 30525 | 29019 | -1.11 | 0.95 | 0.10 | 21710 | 2 | 7 | 0.75 |
| chr9 | 20426 | 19052 | -1.11 | 0.93 | 0.08 | 11222 | 2 | 39 | 0.59 |
| chr10 | 29829 | 28363 | -1.11 | 0.95 | 0.05 | 11171 | 3 | 8 | 0.39 |
| chr11 | 26347 | 24234 | -1.11 | 0.92 | 0.10 | 10757 | 3 | 8 | 0.44 |
| chr12 | 29306 | 28089 | -1.11 | 0.96 | 0.05 | 11028 | 3 | 8 | 0.39 |
| chr13 | 16842 | 16145 | -1.11 | 0.96 | 0.04 | 8803 | 2 | 40 | 0.55 |
| chr14 | 14936 | 14454 | -1.11 | 0.97 | 0.03 | 7705 | 2 | 40 | 0.53 |
| chr15 | 11522 | 10488 | -1.11 | 0.91 | 0.07 | 6189 | 2 | 42 | 0.59 |
| chr16 | 9252 | 7717 | -1.11 | 0.83 | 0.10 | 3544 | 3 | 24 | 0.46 |
| chr17 | 9788 | 7637 | -1.11 | 0.78 | 0.09 | 4519 | 2 | 7 | 0.59 |
| chr18 | 10480 | 9880 | -1.11 | 0.94 | 0.05 | 5485 | 2 | 8 | 0.56 |
| chr19 | 4306 | 2342 | -1.11 | 0.54 | 0.16 | 1226 | 3 | 8 | 0.52 |
| chr20 | 6347 | 5458 | -1.11 | 0.86 | 0.07 | 3189 | 2 | 7 | 0.58 |
| chr21 | 2331 | 1934 | -1.11 | 0.83 | 0.09 | 1172 | 2 | 26 | 0.61 |
| chr22 | 2069 | 1116 | -1.11 | 0.54 | 0.09 | 441 | 4 | 37 | 0.40 |
| chrX | 36496 | 35463 | -1.11 | 0.97 | 0.07 | 16004 | 3 | 13 | 0.45 |

Here, smart local moving algorithm[7] was applied on an intra-chromosomal interaction matrix for predicting the intra-chromosomal community interactions in each chromosome, respectively. The optimal number of communities is accessed by standard modularity score[8]. “Edges”, “Edges_AF”, and “%Edges_AF” are the number of edges before and after filtering 5% of the lowest intra-chromosomal interactions, and the percentages of edges passed filtering. “Minimum” is the minimum Z-score of an edge in an intra-chromosomal interaction matrix. “Modularity” is the standard modularity score of predicted intra-chromosomal community interactions. “Valid clusters” and “Clusters” are valid and total number of network clusters (or intra-chromosomal community), respectively. A “Valid cluster” means a network cluster (intra-chromosomal community) has at least 20 edges. “Edges_VC” and “%Edges_VC” represents the number of and the percentage of “Edges_AF” in valid clusters, respectively.

**STable 8. Summary of intra-chromosomal community interactions after removing the lowest 10 percentages of intra-chromosomal interactions in Hi-C data of one hours E2 treated MCF7 cells.**

|  | Edges | Edges_AF | Minimum | %Edges_AF | Modularity | Edges_VC | Valid clusters | Clusters | %Edges_VC |
| --- | --- | --- | --- | --- | --- | --- | --- | --- | --- |
| chr1 | 61196 | 53620 | -0.98 | 0.88 | 0.14 | 25670 | 3 | 45 | 0.48 |
| chr2 | 86285 | 81940 | -0.98 | 0.95 | 0.09 | 39368 | 3 | 10 | 0.48 |
| chr3 | 60075 | 56429 | -0.98 | 0.94 | 0.07 | 23302 | 3 | 8 | 0.41 |
| chr4 | 53397 | 49903 | -0.98 | 0.94 | 0.08 | 22316 | 3 | 8 | 0.45 |
| chr5 | 52038 | 49697 | -0.98 | 0.96 | 0.06 | 20169 | 3 | 8 | 0.41 |
| chr6 | 40104 | 37551 | -0.98 | 0.94 | 0.08 | 20201 | 3 | 13 | 0.54 |
| chr7 | 35385 | 31125 | -0.98 | 0.88 | 0.09 | 13598 | 3 | 8 | 0.44 |
| chr8 | 30525 | 27387 | -0.98 | 0.90 | 0.11 | 20888 | 2 | 7 | 0.76 |
| chr9 | 20426 | 17929 | -0.98 | 0.88 | 0.09 | 10244 | 2 | 42 | 0.57 |
| chr10 | 29829 | 26568 | -0.98 | 0.89 | 0.06 | 10952 | 3 | 8 | 0.41 |
| chr11 | 26347 | 22263 | -0.98 | 0.85 | 0.11 | 14063 | 2 | 7 | 0.63 |
| chr12 | 29306 | 26716 | -0.98 | 0.91 | 0.06 | 11003 | 3 | 8 | 0.41 |
| chr13 | 16842 | 15080 | -0.98 | 0.90 | 0.06 | 8513 | 2 | 40 | 0.57 |
| chr14 | 14936 | 13806 | -0.98 | 0.92 | 0.04 | 7572 | 2 | 40 | 0.55 |
| chr15 | 11522 | 9475 | -0.98 | 0.82 | 0.10 | 5865 | 2 | 42 | 0.62 |
| chr16 | 9252 | 6590 | -0.98 | 0.71 | 0.13 | 3197 | 3 | 24 | 0.49 |
| chr17 | 9788 | 6328 | -0.98 | 0.65 | 0.11 | 3485 | 3 | 8 | 0.55 |
| chr18 | 10480 | 9219 | -0.98 | 0.88 | 0.07 | 5379 | 2 | 8 | 0.58 |
| chr19 | 4306 | 1660 | -0.98 | 0.39 | 0.22 | 1015 | 3 | 8 | 0.61 |
| chr20 | 6347 | 4730 | -0.98 | 0.75 | 0.10 | 2382 | 3 | 8 | 0.50 |
| chr21 | 2331 | 1717 | -0.98 | 0.74 | 0.11 | 1077 | 2 | 28 | 0.63 |
| chr22 | 2069 | 775 | -0.98 | 0.38 | 0.14 | 340 | 4 | 37 | 0.44 |
| chrX | 36496 | 33878 | -0.98 | 0.93 | 0.07 | 14799 | 3 | 13 | 0.44 |

Here, smart local moving algorithm[7] was applied on an intra-chromosomal interaction matrix for predicting the intra-chromosomal community interactions in each chromosome, respectively. The optimal number of communities is accessed by standard modularity score[8]. “Edges”, “Edges_AF”, and “%Edges_AF” are the number of edges before and after filtering 10% of the lowest intra-chromosomal interactions, and the percentages of edges passed filtering. “Minimum” is the minimum Z-score of an edge in an intra-chromosomal interaction matrix. “Modularity” is the standard modularity score of predicted intra-chromosomal community interactions. “Valid clusters” and “Clusters” are valid and total number of network clusters (or intra-chromosomal community), respectively. A “Valid cluster” means a network cluster (intra-chromosomal community) has at least 20 edges. “Edges_VC” and “%Edges_VC” represents the number of and the percentage of “Edges_AF” in valid clusters, respectively.

**STable 9. Summary of intra-chromosomal community interactions after removing the lowest 20 percentages of intra-chromosomal interactions in Hi-C data of one hours E2 treated MCF7 cells.**

|  | Edges | Edges_AF | Minimum | %Edges_AF | Modularity | Edges_VC | Valid clusters | Clusters | %Edges_VC |
| --- | --- | --- | --- | --- | --- | --- | --- | --- | --- |
| chr1 | 61196 | 46561 | -0.79 | 0.76 | 0.15 | 22811 | 3 | 45 | 0.49 |
| chr2 | 86285 | 75804 | -0.79 | 0.88 | 0.10 | 37364 | 3 | 10 | 0.49 |
| chr3 | 60075 | 51697 | -0.79 | 0.86 | 0.08 | 21805 | 3 | 8 | 0.42 |
| chr4 | 53397 | 44925 | -0.79 | 0.84 | 0.09 | 19471 | 3 | 8 | 0.43 |
| chr5 | 52038 | 46022 | -0.79 | 0.88 | 0.08 | 19374 | 3 | 8 | 0.42 |
| chr6 | 40104 | 34227 | -0.79 | 0.85 | 0.09 | 18966 | 3 | 13 | 0.55 |
| chr7 | 35385 | 26938 | -0.79 | 0.76 | 0.11 | 12482 | 3 | 8 | 0.46 |
| chr8 | 30525 | 24248 | -0.79 | 0.79 | 0.12 | 14500 | 3 | 8 | 0.60 |
| chr9 | 20426 | 15724 | -0.79 | 0.77 | 0.11 | 9717 | 2 | 44 | 0.62 |
| chr10 | 29829 | 23015 | -0.79 | 0.77 | 0.09 | 10257 | 3 | 8 | 0.45 |
| chr11 | 26347 | 18689 | -0.79 | 0.71 | 0.14 | 9033 | 3 | 8 | 0.48 |
| chr12 | 29306 | 23693 | -0.79 | 0.81 | 0.08 | 13867 | 2 | 7 | 0.59 |
| chr13 | 16842 | 13063 | -0.79 | 0.78 | 0.09 | 7768 | 2 | 40 | 0.60 |
| chr14 | 14936 | 12625 | -0.79 | 0.85 | 0.07 | 7265 | 2 | 40 | 0.58 |
| chr15 | 11522 | 7919 | -0.79 | 0.69 | 0.14 | 5284 | 2 | 42 | 0.67 |
| chr16 | 9252 | 4899 | -0.79 | 0.53 | 0.18 | 2713 | 3 | 24 | 0.55 |
| chr17 | 9788 | 4732 | -0.79 | 0.48 | 0.15 | 2887 | 3 | 9 | 0.61 |
| chr18 | 10480 | 8115 | -0.79 | 0.77 | 0.10 | 4973 | 2 | 8 | 0.61 |
| chr19 | 4306 | 1153 | -0.79 | 0.27 | 0.26 | 758 | 3 | 11 | 0.66 |
| chr20 | 6347 | 3828 | -0.79 | 0.60 | 0.13 | 2027 | 3 | 8 | 0.53 |
| chr21 | 2331 | 1384 | -0.79 | 0.59 | 0.15 | 934 | 2 | 29 | 0.68 |
| chr22 | 2069 | 420 | -0.79 | 0.20 | 0.20 | 251 | 3 | 43 | 0.60 |
| chrX | 36496 | 30145 | -0.79 | 0.83 | 0.09 | 13100 | 3 | 13 | 0.44 |

Here, smart local moving algorithm[7] was applied on an intra-chromosomal interaction matrix for predicting the intra-chromosomal community interactions in each chromosome, respectively. The optimal number of communities is accessed by standard modularity score[8]. “Edges”, “Edges_AF”, and “%Edges_AF” are the number of edges before and after filtering 20% of the lowest intra-chromosomal interactions, and the percentages of edges passed filtering. “Minimum” is the minimum Z-score of an edge in an intra-chromosomal interaction matrix. “Modularity” is the standard modularity score of predicted intra-chromosomal community interactions. “Valid clusters” and “Clusters” are valid and total number of network clusters (or intra-chromosomal community), respectively. A “Valid cluster” means a network cluster (intra-chromosomal community) has at least 20 edges. “Edges_VC” and “%Edges_VC” represents the number of and the percentage of “Edges_AF” in valid clusters, respectively.

**STable 10. Summary of intra-chromosomal community interactions after removing the lowest 40 percentages of intra-chromosomal interactions in Hi-C data of one hours E2 treated MCF7 cells.**

|  | Edges | Edges_AF | Minimum | %Edges_AF | Modularity | Edges_VC | Valid clusters | Clusters | %Edges_VC |
| --- | --- | --- | --- | --- | --- | --- | --- | --- | --- |
| chr1 | 61196 | 30954 | -0.48 | 0.51 | 0.22 | 17412 | 3 | 47 | 0.56 |
| chr2 | 86285 | 58500 | -0.48 | 0.68 | 0.15 | 28502 | 3 | 10 | 0.49 |
| chr3 | 60075 | 39049 | -0.48 | 0.65 | 0.15 | 19445 | 3 | 9 | 0.50 |
| chr4 | 53397 | 32848 | -0.48 | 0.62 | 0.16 | 16433 | 3 | 9 | 0.50 |
| chr5 | 52038 | 36049 | -0.48 | 0.69 | 0.14 | 17333 | 3 | 11 | 0.48 |
| chr6 | 40104 | 26751 | -0.48 | 0.67 | 0.14 | 16589 | 3 | 16 | 0.62 |
| chr7 | 35385 | 19499 | -0.48 | 0.55 | 0.19 | 10632 | 3 | 9 | 0.55 |
| chr8 | 30525 | 18935 | -0.48 | 0.62 | 0.17 | 10776 | 3 | 10 | 0.57 |
| chr9 | 20426 | 11700 | -0.48 | 0.57 | 0.17 | 7876 | 2 | 55 | 0.67 |
| chr10 | 29829 | 16944 | -0.48 | 0.57 | 0.15 | 8389 | 3 | 9 | 0.50 |
| chr11 | 26347 | 13218 | -0.48 | 0.50 | 0.20 | 7295 | 3 | 8 | 0.55 |
| chr12 | 29306 | 18316 | -0.48 | 0.63 | 0.11 | 8855 | 3 | 8 | 0.48 |
| chr13 | 16842 | 10090 | -0.48 | 0.60 | 0.13 | 6461 | 2 | 40 | 0.64 |
| chr14 | 14936 | 11086 | -0.48 | 0.74 | 0.10 | 6762 | 2 | 40 | 0.61 |
| chr15 | 11522 | 5931 | -0.48 | 0.52 | 0.18 | 3505 | 3 | 43 | 0.59 |
| chr16 | 9252 | 3451 | -0.48 | 0.37 | 0.21 | 2074 | 3 | 25 | 0.60 |
| chr17 | 9788 | 3207 | -0.48 | 0.33 | 0.20 | 2027 | 3 | 10 | 0.63 |
| chr18 | 10480 | 6444 | -0.48 | 0.62 | 0.15 | 4243 | 2 | 8 | 0.66 |
| chr19 | 4306 | 718 | -0.48 | 0.17 | 0.29 | 529 | 2 | 28 | 0.74 |
| chr20 | 6347 | 2733 | -0.48 | 0.43 | 0.18 | 1522 | 3 | 8 | 0.56 |
| chr21 | 2331 | 948 | -0.48 | 0.41 | 0.19 | 687 | 2 | 29 | 0.73 |
| chr22 | 2069 | 194 | -0.48 | 0.09 | 0.25 | 67 | 2 | 57 | 0.35 |
| chrX | 36496 | 22506 | -0.48 | 0.62 | 0.16 | 11453 | 3 | 13 | 0.51 |

Here, smart local moving algorithm[7] was applied on an intra-chromosomal interaction matrix for predicting the intra-chromosomal community interactions in each chromosome, respectively. The optimal number of communities is accessed by standard modularity score[8]. “Edges”, “Edges_AF”, and “%Edges_AF” are the number of edges before and after filtering 40% of the lowest intra-chromosomal interactions, and the percentages of edges passed filtering. “Minimum” is the minimum Z-score of an edge in an intra-chromosomal interaction matrix. “Modularity” is the standard modularity score of predicted intra-chromosomal community interactions. “Valid clusters” and “Clusters” are valid and total number of network clusters (or intra-chromosomal community), respectively. A “Valid cluster” means a network cluster (intra-chromosomal community) has at least 20 edges. “Edges_VC” and “%Edges_VC” represents the number of and the percentage of “Edges_AF” in valid clusters, respectively.

**STable 11. Summary of intra-chromosomal community interactions after removing the lowest 60 percentages of intra-chromosomal interactions in Hi-C data of one hours E2 treated MCF7 cells.**

|  | Edges | Edges_AF | Minimum | %Edges_AF | Modularity | Edges_VC | Valid clusters | Clusters | %Edges_VC |
| --- | --- | --- | --- | --- | --- | --- | --- | --- | --- |
| chr1 | 61196 | 16791 | 0.03 | 0.27 | 0.27 | 10316 | 3 | 50 | 0.61 |
| chr2 | 86285 | 40728 | 0.03 | 0.47 | 0.21 | 22800 | 3 | 10 | 0.56 |
| chr3 | 60075 | 27923 | 0.03 | 0.47 | 0.19 | 14735 | 3 | 9 | 0.53 |
| chr4 | 53397 | 21217 | 0.03 | 0.40 | 0.21 | 12033 | 3 | 9 | 0.57 |
| chr5 | 52038 | 26458 | 0.03 | 0.51 | 0.18 | 14269 | 3 | 12 | 0.54 |
| chr6 | 40104 | 19023 | 0.03 | 0.47 | 0.18 | 12772 | 3 | 17 | 0.67 |
| chr7 | 35385 | 13469 | 0.03 | 0.38 | 0.24 | 7966 | 4 | 10 | 0.59 |
| chr8 | 30525 | 13865 | 0.03 | 0.45 | 0.21 | 8709 | 3 | 10 | 0.63 |
| chr9 | 20426 | 8218 | 0.03 | 0.40 | 0.22 | 5565 | 3 | 60 | 0.68 |
| chr10 | 29829 | 10485 | 0.03 | 0.35 | 0.21 | 7224 | 3 | 10 | 0.69 |
| chr11 | 26347 | 7952 | 0.03 | 0.30 | 0.24 | 4737 | 3 | 8 | 0.60 |
| chr12 | 29306 | 12268 | 0.03 | 0.42 | 0.16 | 7047 | 3 | 8 | 0.57 |
| chr13 | 16842 | 5674 | 0.03 | 0.34 | 0.21 | 3635 | 3 | 41 | 0.64 |
| chr14 | 14936 | 8159 | 0.03 | 0.55 | 0.17 | 5514 | 2 | 40 | 0.68 |
| chr15 | 11522 | 3146 | 0.03 | 0.27 | 0.26 | 1984 | 3 | 47 | 0.63 |
| chr16 | 9252 | 1661 | 0.03 | 0.18 | 0.29 | 1064 | 3 | 29 | 0.64 |
| chr17 | 9788 | 1678 | 0.03 | 0.17 | 0.27 | 1245 | 2 | 22 | 0.74 |
| chr18 | 10480 | 3883 | 0.03 | 0.37 | 0.23 | 2670 | 2 | 10 | 0.69 |
| chr19 | 4306 | 416 | 0.03 | 0.10 | 0.25 | 304 | 2 | 61 | 0.73 |
| chr20 | 6347 | 1557 | 0.03 | 0.25 | 0.25 | 1061 | 3 | 11 | 0.68 |
| chr21 | 2331 | 511 | 0.03 | 0.22 | 0.24 | 332 | 3 | 36 | 0.65 |
| chr22 | 2069 | 43 | 0.03 | 0.02 | 0.37 | 0 | 0 | 80 | 0.00 |
| chr23 | 36496 | 14937 | 0.03 | 0.41 | 0.19 | 8134 | 3 | 14 | 0.55 |

Here, smart local moving algorithm[7] was applied on an intra-chromosomal interaction matrix for predicting the intra-chromosomal community interactions in each chromosome, respectively. The optimal number of communities is accessed by standard modularity score[8]. “Edges”, “Edges_AF”, and “%Edges_AF” are the number of edges before and after filtering 60% of the lowest intra-chromosomal interactions, and the percentages of edges passed filtering. “Minimum” is the minimum Z-score of an edge in an intra-chromosomal interaction matrix. “Modularity” is the standard modularity score of predicted intra-chromosomal community interactions. “Valid clusters” and “Clusters” are valid and total number of network clusters (or intra-chromosomal community), respectively. A “Valid cluster” means a network cluster (intra-chromosomal community) has at least 20 edges. “Edges_VC” and “%Edges_VC” represents the number of and the percentage of “Edges_AF” in valid clusters, respectively.

**STable 12. Summary of intra-chromosomal community interactions after selecting significant intra-chromosomal interactions in Hi-C data of one hours E2 treated MCF7 cells by HOMER**

|  | Edges | Modularity | Edges_VC | Valid clusters | Clusters | %Edges_VC |
| --- | --- | --- | --- | --- | --- | --- |
| chr1 | 6386 | 0.51 | 5554 | 4 | 91 | 0.87 |
| chr2 | 16656 | 0.38 | 12254 | 3 | 16 | 0.74 |
| chr3 | 10880 | 0.38 | 7807 | 4 | 21 | 0.72 |
| chr4 | 9453 | 0.43 | 6620 | 4 | 16 | 0.70 |
| chr5 | 11332 | 0.36 | 8267 | 3 | 14 | 0.73 |
| chr6 | 8541 | 0.31 | 6251 | 4 | 21 | 0.73 |
| chr7 | 7045 | 0.40 | 5481 | 4 | 23 | 0.78 |
| chr8 | 7398 | 0.30 | 4601 | 4 | 33 | 0.62 |
| chr9 | 4977 | 0.31 | 3257 | 3 | 69 | 0.65 |
| chr10 | 4889 | 0.37 | 3670 | 3 | 11 | 0.75 |
| chr11 | 5337 | 0.39 | 3597 | 4 | 24 | 0.67 |
| chr12 | 4885 | 0.33 | 3161 | 4 | 19 | 0.65 |
| chr13 | 3768 | 0.33 | 2316 | 4 | 42 | 0.62 |
| chr14 | 4931 | 0.29 | 3605 | 3 | 44 | 0.73 |
| chr15 | 1517 | 0.43 | 1105 | 5 | 55 | 0.73 |
| chr16 | 1350 | 0.46 | 1119 | 3 | 34 | 0.83 |
| chr17 | 822 | 0.32 | 604 | 3 | 61 | 0.74 |
| chr18 | 3666 | 0.23 | 2433 | 3 | 11 | 0.66 |
| chr19 | 751 | 0.23 | 598 | 2 | 47 | 0.80 |
| chr20 | 800 | 0.42 | 688 | 3 | 42 | 0.86 |
| chr21 | 564 | 0.23 | 368 | 3 | 38 | 0.65 |
| chr22 | 208 | 0.55 | 143 | 4 | 45 | 0.69 |
| chr23 | 9829 | 0.35 | 7079 | 3 | 18 | 0.72 |

Here, smart local moving algorithm[7] was applied on an intra-chromosomal interaction matrix for predicting the intra-chromosomal community interactions in each chromosome, respectively. The optimal number of communities is accessed by standard modularity score[8]. “Edges” are the number of edges selected by HOMER. “Modularity” is the standard modularity score of predicted intra-chromosomal community interactions. “Valid clusters” and “Clusters” are valid and total number of network clusters (or intra-chromosomal community), respectively. A “Valid cluster” means a network cluster (intra-chromosomal community) has at least 20 edges. “Edges_VC” and “%Edges_VC” represents the number of and the percentage of “Edges_AF” in valid clusters, respectively.

**STable 13. Minimal number edge of a valid community in different resolutions and conditions.**

|  | 500kb | 100kb | 50kb | *P* |
| --- | --- | --- | --- | --- |
| t0 / untreated | 74 | 383 | 1339 | 0.05 |
|  | **23** | **153** | **480** | **0.02** |
|  | 18 | 93 | 257 | 0.01 |
| t1 | 43 | 163 | 542 | 0.05 |
|  | **20** | **74** | **196** | **0.02** |
|  | 10 | 33 | 109 | 0.01 |
| TAMR | 48 | 250 | 640 | 0.05 |
|  | **22** | **78** | **306** | **0.02** |
|  | 13 | 42 | 142 | 0.01 |

The minimum number edges in a valid community at different conditions (e.g., t0, t1, and TAMR represent untreated, one-hours E2 treated MCF7 cells and tamoxifen-resistant MCF7TR, respectively) and in different resolutions (e.g., bin size = 500kb, 100kb, 50kb) are calculated automatically by to the proposed method in the manuscript, by using a predefined cutoff value (e.g., *p* = 0.01, 0.02 or 0.05). The first column of the table is the condition of experiments or sample name. The first row of the table is the resolutions (or bin size) of the calculation and the proportion *p*, respectively. The bold rows represent the cutoffs used in this work.

**STable 14. RV-coefficient for genomic feature enrichment heatmaps or matrices between the mean- and the median-mapping of Z-scores to Hi-C interaction matrices.**

|  | RV (500kb, untreated) | RV (50kb, untreated) | RV (500kb, TAMR) | RV (50kb, TAMR) |
| --- | --- | --- | --- | --- |
| chr1 | 0.73 | 0.94 | 0.26 | 0.83 |
| chr2 | 0.59 | 1.00 | 0.36 | 0.87 |
| chr3 | 0.76 | 0.97 | 0.34 | 0.71 |
| chr4 | 0.63 | 0.93 | 0.37 | 0.61 |
| chr5 | 0.80 | 0.98 | 0.37 | 0.80 |
| chr6 | 0.64 | 0.97 | 0.27 | 0.95 |
| chr7 | 0.77 | 0.93 | 0.51 | 0.74 |
| chr8 | 0.62 | 0.88 | 0.58 | 0.85 |
| chr9 | 0.88 | 0.95 | 0.35 | 0.52 |
| chr10 | 0.71 | 0.94 | 0.37 | 0.87 |
| chr11 | 0.81 | 0.88 | 0.70 | 0.83 |
| chr12 | 0.75 | 0.99 | 0.23 | 0.86 |
| chr13 | 0.73 | 0.89 | 0.50 | 0.75 |
| chr14 | 0.88 | 0.86 | 0.46 | 0.69 |
| chr15 | 0.83 | 0.99 | 0.36 | 0.70 |
| chr16 | 0.65 | 0.96 | 0.39 | 0.81 |
| chr17 | 0.83 | 0.74 | 0.17 | 0.52 |
| chr18 | 0.66 | 0.87 | 0.79 | 0.92 |
| chr19 | 0.71 | 0.80 | 0.31 | 0.57 |
| chr20 | 0.47 | 0.95 | 0.36 | 0.81 |
| chr21 | 0.52 | 0.92 | 0.28 | 0.83 |
| chr22 | 0.64 | 0.93 | 0.16 | 0.66 |
| chr23 | 0.93 | 0.64 | 0.50 | 0.72 |

Here, a similarity of genomic feature enrichment heatmaps or matrices between the mean- and the median-mapping of Z-scores to Hi-C interaction matrices is evaluated by RV-coefficient (e.g., $RV\in[0,1]$). In the table, each column represents RV-coefficient obtained in a specific condition (e.g., untreated and TAMR represent untreated MCF7 cells and tamoxifen-resistant MCF7TR, respectively) and interaction resolution (e.g., window bin size equals to 500kb or 50kb).

**Supplementary References**

1. Wang, X.W., et al., *A statistical physics approach for disease module detection.* Genome Res, 2022. **32**(10): p. 1918-1929.

2. Yao, Z., J. Zhang, and X. Zou, *A general index for linear and nonlinear correlations for high dimensional genomic data.* BMC Genomics, 2020. **21**(1): p. 846.

3. Smilde, A.K., et al., *Matrix correlations for high-dimensional data: the modified RV-coefficient.* Bioinformatics, 2009. **25**(3): p. 401-5.

4. Newman, M.E.J. and M. Girvan, *Finding and evaluating community structure in networks.* Physical Review E, 2004. **69**(2).

5. Heinz, S., et al., *Simple Combinations of Lineage-Determining Transcription Factors Prime cis-Regulatory Elements Required for Macrophage and B Cell Identities.* Molecular Cell, 2010. **38**(4): p. 576-589.

6. Kruse, K., C.B. Hug, and J.M. Vaquerizas, *FAN-C: a feature-rich framework for the analysis and visualisation of chromosome conformation capture data.* Genome Biol, 2020. **21**(1): p. 303.

7. Waltman, L. and N.J. van Eck, *A smart local moving algorithm for large-scale modularity-based community detection.* European Physical Journal B, 2013. **86**(11).

8. Girvan, M. and M.E.J. Newman, *Community structure in social and biological networks.* Proceedings of the National Academy of Sciences of the United States of America, 2002. **99**(12): p. 7821-7826.
